# Transcriptomic Landscape of Cisplatin-Resistant Neuroblastoma Cells

**DOI:** 10.3390/cells8030235

**Published:** 2019-03-12

**Authors:** Miguel Angel Merlos Rodrigo, Hana Buchtelova, Ana Maria Jimenez Jimenez, Pavlina Adam, Petr Babula, Zbynek Heger, Vojtech Adam

**Affiliations:** 1 Department of Chemistry and Biochemistry, Mendel University in Brno, Zemedelska 1, CZ-613 00 Brno, Czech Republic; merlos19792003@gmail.com (M.A.M.R.); hanabuchtelova@seznam.cz (H.B.); anuskajj@hotmail.com (A.M.J.J.); Pavlina.adam@email.cz (P.A.); heger@mendelu.cz (Z.H.); 2 Central European Institute of Technology, Brno University of Technology, Technicka 3058/10, CZ-616 00 Brno, Czech Republic; babula@med.muni.cz; 3 Department of Physiology, Faculty of Medicine, Masaryk University, Kamenice 753/5, CZ-625 00 Brno, Czech Republic

**Keywords:** neuroblastoma, cisplatin, chemoresistance, microarray, lysosomes, transport

## Abstract

The efficiency of cisplatin (CDDP) is significantly hindered by the development of resistance during the treatment course. To gain a detailed understanding of the molecular mechanisms underlying the development of cisplatin resistance, we comparatively analyzed established a CDDP-resistant neuroblastoma cell line (UKF-NB-4^CDDP^) and its susceptible parental cells (UKF-NB-4). We verified increased chemoresistance of UKF-NB-4^CDDP^ cells by analyzing the viability, induction of apoptosis and clonal efficiency. To shed more light on this phenomenon, we employed custom cDNA microarray (containing 2234 probes) to perform parallel transcriptomic profiling of RNA and identified that 139 genes were significantly up-regulated due to CDDP chemoresistance. The analyses of molecular pathways indicated that the top up-regulation scoring functions were response to stress, abiotic stimulus, regulation of metabolic process, apoptotic processes, regulation of cell proliferation, DNA repair or regulation of catalytic activity, which was also evidenced by analysis of molecular functions revealing up-regulation of genes encoding several proteins with a wide-spectrum of enzymatic activities. Functional analysis using lysosomotropic agents chloroquine and bafilomycin A1 validated their potential to re-sensitize UKF-NB-4^CDDP^ cells to CDDP. Taken together, the identification of alterations in specific genes and pathways that contribute to CDDP chemoresistance may potentially lead to a renewed interest in the development of novel rational therapeutics and prognostic biomarkers for the management of CDDP-resistant neuroblastoma.

## 1. Introduction

Neuroblastoma (Nbl) arises from early neural crest precursors and is typical for a broad spectrum of symptoms and poor prognosis. Nbl is the third most common childhood cancer after leukemia and cancers of the central nervous system [1,2,3,4]. The majority of the chemotherapeutic protocols used in Nbl management rely on compounds that alter a progression of cell cycle, and synthesis of DNA, resulting in DNA damage followed by activation of programmed cell death pathways [2,5]. Cisplatin or cis-diamminedichloroplatinum (CDDP) belongs to the most prominent anticancer drugs, since it displays great anticancer activity in a wide spectrum of cancer types, such as ovarian cancers, solid tumors of the head and neck and Nbl [6]. CDDP and other coordination compounds based on platinum are known as highly cytotoxic agents with the ability to damage DNA and inhibit DNA synthesis with consequent effects on mitosis and apoptotic cell death [7]. Patients that undergo treatment with CDDP usually respond well to CDDP chemotherapy, but later relapses can occur due to the development of CDDP chemoresistance, which can be either acquired or intrinsic and significantly affects the clinical outcomes of CDDP therapy [8,9,10].

Chemoresistance is an important obstacle in anticancer therapy. Despite a number of distinct chemotherapeutics, and the fact that endocrine regimens and targeted anticancer drugs have been developed, chemoresistance is still a fundamental complication associated with the therapeutic protocols utilizing chemical agents. CDDP resistance could arise from a wide spectrum of causes, these include a decrease in blood flow of tumor mass, extracellular environment, inhibited internalization of CDDP, exocytosis, intracellular sequestration of CDDP by metallothioneins (MTs), altered DNA reparation or apoptosis-driving mechanisms, or a presence of quiescent and non-cycling cells [11,12,13,14,15,16,17,18,19,20].

The delineation of novel molecular targets that participate on the aggressiveness of Nbl, together with the development of agents that will influence the functionality of such targets, is of utmost interest towards improvement of patient survival [21]. Our previous study demonstrated that the transient up-regulation of metallothionein-3 (MT-3) in Nbl cells potently induces chemoresistance to CDDP, revealing a great potential for further investigation of MTs for use as biomarkers of CDDP [22]. CDDP affects several important pathways, which stimulate molecular mechanisms, which are responsible for the development of CDDP chemoresistance by developing a complex self-defense network to escape exogenous cytotoxic substances of distinct origin. Moreover, the detailed understanding of these phenomena is of utmost importance for identification of novel druggable targets that can be altered by small molecule inhibitors or down-regulated by small interfering ribonucleic acids. 

Among the promising agents belong compounds referred to as lysosomotropic agents. As weak bases, these substances have the capability to penetrate inside acidic environment of lysosomes and accumulate as protonated forms. This leads to the elevation of intralysosomal pH and alteration of lysosomal functions, including the fusion of lysosome with the autophagosome [23,24]. It is worth noting that lysosomotropic agents have found their place in a number of clinical applications, such as antimalarial therapy or treatment of rheumatoid arthritis [chloroquine (CQ), hydroxyCQ] [25]. These substances are also highly promising viral entry inhibitors [26]. Moreover, inhibition of lysosomal functions has been shown to be an effective therapeutic strategy in several chemoresistant cancer models [27]. Despite that, to meet the aims of personalized medicine, further investigations are required with a particular focus on the suitable biomarkers and applicability of these compounds in a combination anticancer therapy [28].

Herein, we report profiling of a transcriptomic landscape of CDDP-resistant high-risk Nbl cells (UKF-NB-4^CDDP^) and their respective drug-sensitive parental line (UKF-NB-4). UKF-NB-4^CDDP^ exhibited only negligible susceptibility to CDDP administration as evidenced by viability screenings, quantitation of apoptosis induction and evaluation of clonal efficiency. A cDNA profiling revealed that CDDP chemoresistance has a specific transcriptomic signature involving genes responsible for neurogenesis, metal ion trafficking, exocytosis and DNA repair.

## 2. Materials and Methods

### 2.1. Chemicals

All chemicals and reagents were purchased from Sigma-Aldrich (St. Louis, MO, USA) in ACS purity, unless noted otherwise.

### 2.2. Cell Lines and Culture Conditions

The UKF-NB-4 cell line, which was established from recurrent bone marrow metastases of high-risk Nbl (stage IV, *MYCN* amplification, 7q21 gain), was a kind gift by prof. J. Cinatl, DrSc. from the Goethe University in Frankfurt am Main, Germany. The UKF-NB-4^CDDP^ cell line was established from parental UKF-NB-4 cells in the laboratory of prof. T. Eckschlager by incubating the cells with gradually increasing concentrations of CDDP. The cells were grown at 37 °C and 5% CO_2_ in Iscove’s modified Dulbecco’s medium (IMDM) with 10% bovine serum. UKF-NB-4^CDDP^ cells were cultivated in IMDM with CDDP (100 ng/mL). The cell lines were passaged at regular intervals twice a week. 

### 2.3. Effect of Cisplatin (CDDP) Administration on Viability of Nbl Cells

The suspension of approximately 5000 cells was added to each well of microtiter plates. Cultures were incubated for 2 days at 37 °C to ensure cell growth. The medium was replaced with medium containing annotated concentrations of CDDP dissolved in 0.9% NaCl solution (*w*/*v*). Upon 24 h incubation, the medium was replaced with 200 µL of fresh IMDM containing 50 µL of 3-(4,5-dimethylthiazol-2-yl)-2,5-diphenyltetrazolium bromide [MTT (5 mg/mL)] and the cells were incubated for 4 h at 37 °C. The MTT-containing medium was replaced with 200 µL of 99.9% (*v*/*v*) dimethyl sulfoxide to dissolve MTT-formazan crystals. The absorbance was recorded at 570 nm was determined (Infinite 200PRO, Tecan, Maennedorf, Switzerland). All analyses were carried out in six replicates (*n* = 6). Results are presented as percent of cell viability. The viability was also validated by trypan blue exclusion (0.4%, *w*/*w*) and counted with Countess FL II instrument (Thermo Fisher Scientific, Waltham, MA, USA).

### 2.4. Flow Cytometry

A suspension of 500,000 cells was added to each 25 mL flask. After 6 h incubation with CDDP (20 µg/mL), apoptosis was determined in 10,000 events using the BD Accuri C6 Plus flow cytometer (BD Biosciences, Franklin Lakes, NJ, USA) and PE Annexin V Apoptosis Detection Kit I (BD Biosciences) according to the manufacturer’s instructions.

### 2.5. Clonogenic Assay

Cells were seeded in a 6-well plate at a density of 1 × 10^4^ cells per well in IMDM and incubated for 6 h. Then, the cells were pre-incubated with CDDP for 24 h. After medium renewal, the cells were incubated for further 8 days. Finally, the cells were washed with phosphate buffered saline (PBS) and fixed using 500 µL of 3:1 methanol:acetic acid for 5 min. The cells were finally analyzed using phase contrast micrographs with an EVOS FL Auto Cell Imaging System (Thermo Fisher Scientific). 

### 2.6. Isolation of RNA and Reverse Transcription

A high-purity total-RNA isolation kit (Roche, Basel, Switzerland) was used for the isolation of cellular RNA. The medium was removed and samples were twice washed with 5 mL of ice-cold PBS. Cells were scraped off, transferred to clean tubes and centrifuged at 20,800× *g* for 5 min at 4 °C. After that, lysis buffer was added and RNA isolation was carried out according to the manufacturer’s instructions. RNA (500 ng) was transcribed using Transcriptor First Strand cDNA Synthesis Kit (Roche) according to manufacturer’s instructions. Prepared cDNA (20 µL) was diluted with RNase free water to a total volume of 100 µL. 5 µL of this solution was employed for quantitative reverse transcription polymerase chain reaction (qRT-PCR) and microarrays.

### 2.7. cDNA Microarray

The cDNA obtained was biotinylated on its 3′ end using Biotin 3′ End DNA labeling kit (Thermo Fisher Scientific) following the manufacturer’s instructions. For hybridization, ElectraSense 4 × 2k array slides with 2234 immobilized DNA probes (Custom Array, Bothell, WA, USA) were utilized. The full list of genes present within the microarray chip is shown in Table S1. For customizing the microarrays chips, the genes included in the major hallmarks of cancer were selected with a special emphasis on metabolism, DNA repair, cell death, proliferation, cell cycle control, epigenetic regulation, metal homeostasis, drug efflux and others. The rationale behind this selection was based on the hypothesis that these pathways could be deregulated due to CDDP. Prior to the analyses, the hybridization chamber was filled with fresh pre-hybridization solution (2× hybridization solution stock, 6× saline–sodium phosphate–ethylenediaminetetraacetic acid (EDTA), 0.05% Tween-20, 20 mM EDTA in nuclease-free water, 5× Denhardt’s solution, 100 ng/μL salmon sperm DNA, and 0.05% sodium dodecyl sulfate). Then, the microarray was loaded onto the rotisserie in the hybridization oven and incubated at the desired hybridization temperature for 30 min with gentle rotation. Hybridization solution containing 10 to 40 ng/μL labeled targets was prepared and denatured at 95 °C for 3 min and then cooled for 1 min on ice. Furthermore, the hybridization chamber was filled with the hybridization solution, and the microarray was loaded onto the rotisserie in the hybridization oven and incubated at 50 °C for 16 h with gentle rotation. After the hybridization, the chamber was rinsed using saline-sodium phosphate-EDTA-Tween and PBS-Tween to remove weakly bound DNA. Post-hybridization, blocking buffer was added to the hybridization chamber and the array was incubated at 25 °C for 15 min. Upon the incubation, the biotin labeling solution was added to the chamber and the chips were incubated at 25 °C for 30 min. After rinsing the chambers and subsequent filling with biotin wash solution, the chambers incubated at 25 °C for 5 min. The detection was accomplished using the CombiMatrix ElectraSense^TM^ Detection Kit (CombiMatrix, Mukilteo, WA, USA) using the ElectraSense^TM^ Reader (CombiMatrix) that amperometrically detects current flux for each individual spot through the underlying platinum microelectrode. The cDNA microarray raw data are available and can be provided upon request from the corresponding author. 

### 2.8. qRT-PCR

Gene expression was validated by qRT-PCR using the SYBR Green Quantitative RT-PCR Kit (Sigma-Aldrich, St. Louis, MO, USA) and the Mastercycler pro S instrument (Eppendorf, Hamburg, Germany). The specificity of the qPCR was checked by melting curve analysis and the relative levels of transcription were calculated using the 2^−ΔΔCT^ method [29]. The list of primers used for validation of microarray data is shown in Table S2. The qPCR experiments were performed in conditions described in our previous study [22].

### 2.9. Survival Analysis

Kaplan-Meier plots representing the probability of overall survival of primary Nbl patients stratified according to the expression of top three up- (*SHFM1*, *PSMD14* and *CAV2*) and down-regulated (*CASP8*, *SOCS1* and *DSC2*) genes identified in microarrays were calculated with the R2: Genomics Analysis and Visualization Platform (r2.amc.nl) using Versteeg NB88 neuroblastoma public cohort (MAS5.0–u133p2) [30]. The log-rank test was utilized to assess the significance of the correlation between the expression of analysed genes and survival outcomes.

### 2.10. Western Blotting

Total cellular proteins were extracted with 100 µL of RIPA buffer containing protease inhibitor cocktail. After electrophoresis, the proteins were electrotransferred onto a polyvinylidene fluoride membrane that was blocked with 5% (*w*/*v*) non-fat dry milk for 1 h at 37 °C. The membranes were incubated with primary rabbit anti-LC3-I/II (dilution 1:2000, ABC929, Merck Millipore, Burlington, MA, USA) and rabbit anti-β-Actin (1:700, ab8227, Abcam, Cambridge, UK) overnight at 4 °C. After washing, the membranes were incubated with anti-rabbit horseradish peroxidase-conjugated secondary antibodies (Dako, Glostrup, Denmark) for 1 h at 20 °C. Proteins were detected using Clarity Western ECL substrate (Bio-Rad, Hercules, CA, USA) and visualized using the Azure c600 imager (Azure Biosystems).

### 2.11. Examination of Re-Sensitizing of UKF-NB-4^CDDP^ Cells Using Lysosomotropic Agents

To validate the microarray data, we utilized lysosomotropic agents CQ (25 µM) and bafilomycin A1 (Baf, 10 nM). To evaluate synergistic effects, CDDP (40 µg/mL) was administered alone or with the aforementioned agents. Viability of UKF-NB-4 and UKF-NB-4^CDDP^ was evaluated upon 24 treatments by trypan blue exclusion (0.4%, *w*/*w*) and the amount of death cells was counted with Countess FL II instrument (Thermo Fisher Scientific).

### 2.12. Descriptive Statistics and Exploited Bioinformatic Tools

For the statistical evaluation of the results, the mean was taken as the measurement of the main tendency, while standard deviation was taken as the dispersion measurement. Differences between groups were analyzed using the paired t-test and analysis of variance (ANOVA). Microarrays were performed as three independent biological analyses from three replicate experiments. The raw data from scanned arrays were analyzed by the ElectraSense^TM^ software package (version 6.2.6) independently in comparison with different types of genes assuming that each gene is unique. The averages of all normalized probe intensities within the same probe sets and their standard deviations were calculated for each probe. The ElectraSense™ application software created a microarray image in grey scale for visualization purposes. The microarray results were presented as the median ± standard deviation (SD) of the number of experiments shown in the caption. The list of processes and/or pathways driven by regulated genes was analysed for a whole dataset (2234 probes) and created by Gene Ontology (GO) annotations and Kyoto Encyclopedia of Genes and Genomes (KEGG) 10 software (Known and Predicted Protein-Protein Interactions), respectively. The interactome networks were constructed using Search Tool for the Retrieval of Interacting Genes/Proteins (STRING, version 11.0). The involvement of genes in a cellular process, their molecular functions and cellular component organization was analysed using Blast2GO version 5Pro.

## 3. Results

### 3.1. Induction of CDDP Chemoresistance Results in Altered Morphology and Clonal Efficiency of UKF-NB-4 Cells

**Figure 1:**
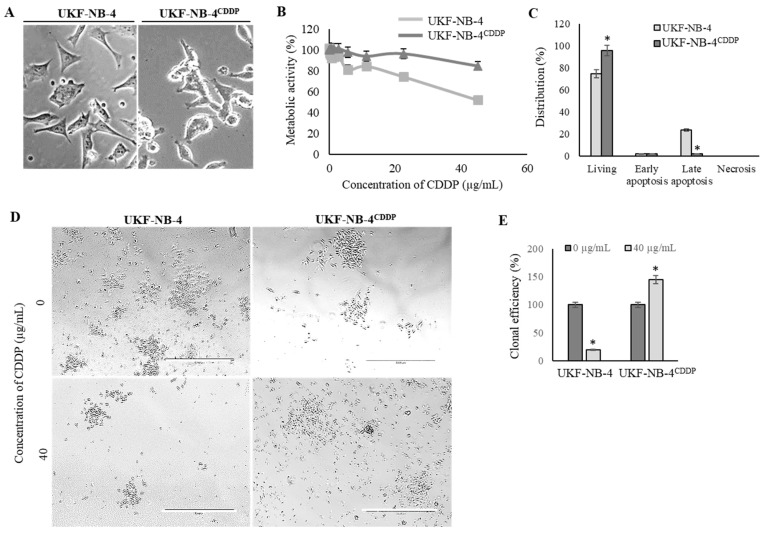
Revalidation of effects of induced cisplatin (CDDP) chemoresistance on phenotypic properties of UKF-NB-4 cells. (**A**) Phase contrast micrographs showing the morphology changes in UKF-NB-4^CDDP^ cells. Scale bar, 100 µm. (**B**) MTT dose-response curves of UKF-NB-4 and UKF-NB-4^CDDP^ cells exposed to CDDP (1–50.0 µg/mL) for 24 h. (**C**) Distribution of CDDP-induced apoptosis and living cell counts in both tested cell lines evaluated by flow cytometry. The data are results from three independent experiments. * *p* < 0.01 related to parental cells. (**D**) Detailed representative phase contrast micrographs of clonogenic assay plates showing significant differences in survival and clonal efficiency. The length of scale bar is 1000 µm. (**E**) Quantitation of clonal efficiency of cells exposed to CDDP. The data are results from three independent experiments. * *p* < 0.05 related to control non-treated cells.

We revalidated the marked phenotypical alterations in UKF-NB-4^CDDP^ cells compared to the parental cells [22,31]. Figure 1A illustrates that induction of CDDP chemoresistance results in pronounced morphological changes from initial polygonal morphology of UKF-NB-4 cells to more globular and less-elongated UKF-NB-4^CDDP^ cells. Moreover, UKF-NB-4^CDDP^ cells exhibit lower susceptibility to CDDP exposure as demonstrated by dose-response curves (Figure 1B). This phenomenon is also confirmed by the evaluation of induction of overall cell death as illustrated in Figure 1C. Large differences were also identified by the analysis of clonogenicity, in which parental cell line exhibited a significant (*p* < 0.05) inhibition of clonal efficiency (Figure 1D). Contrary to that, UKF-NB-4^CDDP^ cells were able to escape the inhibitory effects of CDDP and formed new colonies during 8 days of culturing (Figure 1E). Overall, we confirmed significant disparities in phenotypic behavior in both cell lines; hence, we further analyzed the transcriptomic patterns associated with these properties in the following experiments.

### 3.2. UKF-NB-4^CDDP^ Cells Exhibit Widely Altered Transcriptomic Profile Compared with Parental Cells

In order to investigate the relative quantitative differences in genes’ expression resulting from CDDP resistance of UKF-NB-4 cells, the transcriptomic profiles were obtained and analyzed. Figure S1 depicts the representative microarray heatmaps demonstrating obvious differences in cDNA hybridization efficiency (i.e., gene expression, one spot represents one probe) within the tested cells.

**Figure 2:**
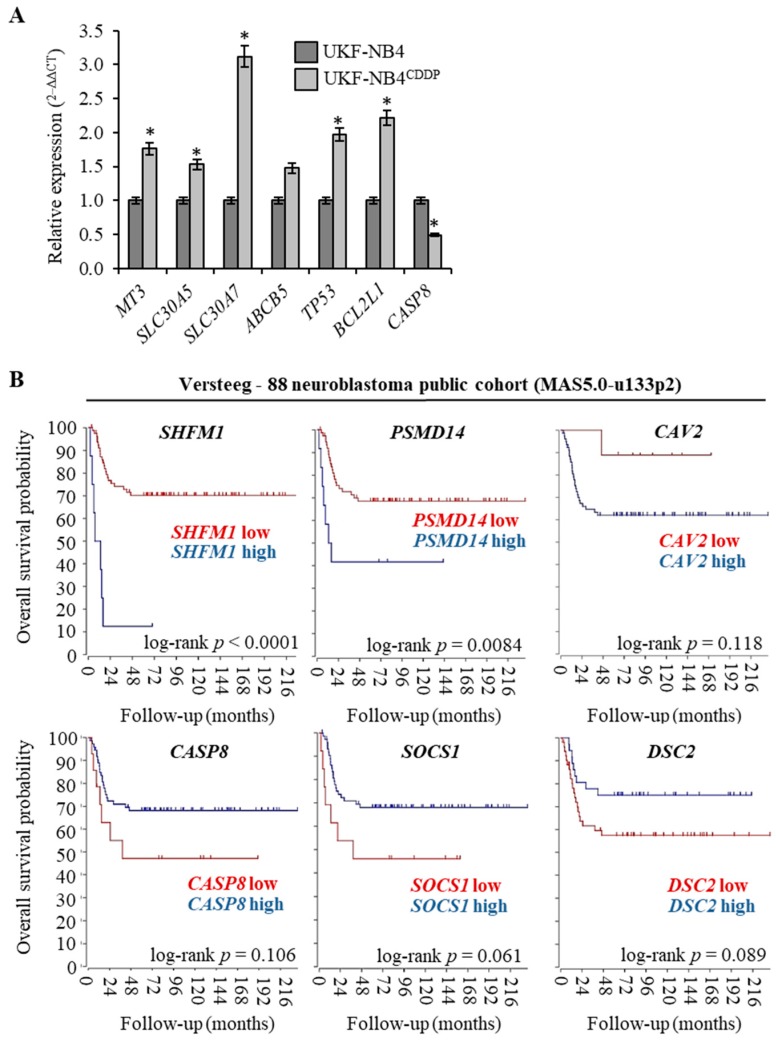
Analysis and validation of cDNA microarray data. (**A**) The quantitative polymerase chain reaction (qPCR) validation of expression of six up-regulated and one down-regulated genes identified by microarray analysis. The data are results from three independent experiments. * *p* < 0.01 related to parental cells. (**B**) Kaplan-Meier curves showing the overall survival of Nbl patients (*n* = 88) with tumors expressing *SHFM1*, *PSMD14* and *CAV2* (genes up-regulated in UKF-NB-4^CDDP^ cells, *top panel*) and *CASP8*, *SOCS1* and *DSC2* (genes down-regulated in UKF-NB-4^CDDP^ cells, *bottom panel*). The *p* values were determined using a log-rank test. The R2: Genomics Analysis and Visualization Platform was used for the calculation. Versteeg NB88 neuroblastoma public cohort (MAS5.0-u133p2) was the source of the data.

The full list of genes (*n* = 139) that were found to be up-regulated in UKF-NB-4^CDDP^ cells in four independent analyses (*n* = 4) is shown in Table 1 along with their accession numbers and fold ratio rating. Medians, whose fold ratio was ≥1.5 compared with parental cell line, were exploited as a threshold for up-regulation. Microarray analyses revealed also genes (*n* = 4) that could be classified as down-regulated; reaching the threshold expression ≤1.5. The complete list of up- and down-regulated genes served as input for further bioinformatic analyses. To validate microarray data, qPCR analyses of selected targets was conducted (Figure 2A). We particularly focused on targets that are known metal ions transporters, and therefore can contribute to trafficking of CDDP (*MT-3*, *SLC30A5*, *SLC30A7*, *ABCB5*) and on genes that are known to be involved in apoptosis (*TP53*, *BCL2L1* and *CASP8*). We anticipate that the analyzed metal ion transporters could be activated due to CDDP chemoresistance to alleviate stress caused by CDDP administration. It is noteworthy that all of the results corroborated the microarray analyses and confirmed the expression altered by acquired CDDP chemoresistance. To test whether top up- and down-regulated genes associated with CDDP chemoresistance could serve as prognostic biomarkers, we performed survival meta-analysis utilizing public cohort dataset of 88 Nbl patients. Interestingly, Figure 2B illustrates that the expression of analysed genes closely associates with Nbl prognosis with the highest significance for *SHFM1* (log-rank *p* < 0.0001) and *PSMD14* (log-rank *p* = 0.0084). Overall, these data suggest that the analysed genes might serve not only as predictors of Nbl progression, but also the efficiency of CDDP-based therapy.

### 3.3. Pathway, Function and Cellular Component Analyses of Genes Up-Regulated in UKF-NB-4^CDDP^ Cells

**Figure 3:**
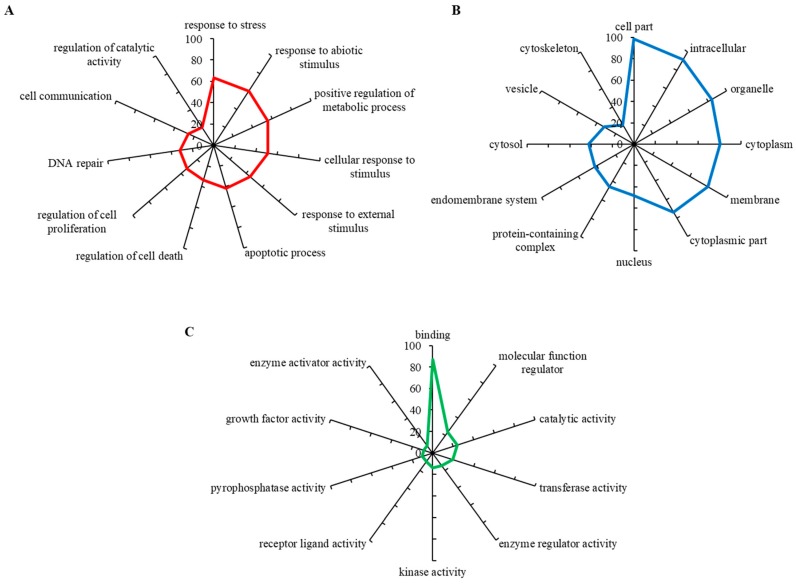
Gene Ontology (GO) analysis of (**A**) biological processes, (**B**) molecular functions and (**C**) cellular components organization of genes up-regulated in UKF-NB-4^CDDP^ cells. For analyses using Blast2GO version 5Pro the outputs with the lowest false discovery rate were employed. The axis describes the gene counts (%).

To evaluate biological relevance of the differentially expressed genes, we carried out a GO annotation analysis, which identifies the involvement of particular genes within biological processes, molecular functions and cellular components. The up-regulated gene set was found to mostly include the genes involved in response to stress, response to abiotic stimulus, positive regulation of metabolic stress and others (Figure 3A). Figure 3B shows that most hits belonged to the intracellular region, particularly organelles and cytoplasm, while some also belonged to vesicles and protein-containing complexes. By means of molecular functions, up-regulated genes encoded proteins having a wide spectrum of enzyme activities (kinase, transferase, pyrophosphatase, catalytic) (Figure 3C). These data indicate that CDDP-chemoresistant Nbl cells are capable of developing a powerful enzymatic machinery that markedly alleviates the cytotoxic activity of CDDP.

**Figure 4:**
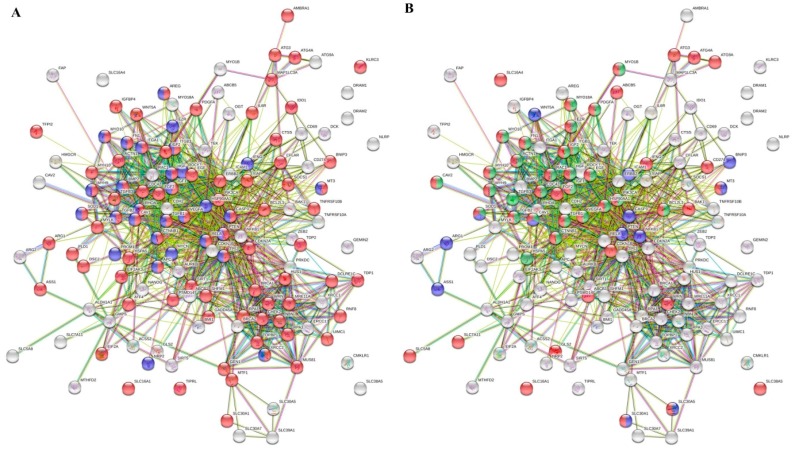
Interactome networks of genes up-regulated in UKF-NB-4^CDDP^ cells. (**A**) The red and blue nodes highlight the genes, which are involved in response to DNA repair and neurogenesis, respectively. (**B**) The red, green and blue nodes highlight the genes, which are involved in metal ion transport, vesicles-mediated transport and response to metal ions, respectively. The color of the line provides evidence of the different interactions among translated proteins. A red line indicates the presence of fusion evidence; a green line, neighborhood evidence; a blue line, concurrence evidence; a purple line, experimental evidence; a light blue line, database evidence; a black line, co-expression evidence.

Furthermore, to prioritize the differentially expressed genes involved in transport and regulation of cellular biosynthetic process, which are the major hallmarks of every type of cancer, we utilized the STRING database of known and predicted interactions. Figure 4A illustrates the interactome network, where the red and blue nodes highlight the genes that are involved in DNA repair (particularly *BRCA1*, *BRCA2*, *CHEK2*, *DCLRE1C*, *GADD45A*, *GEN1*, *HUS1*, *MRE11A*, *MUS81*, *NBN*, *PRKDC*, *PSMD14*, *RNF8*, *RPA1*, *RPA3*, *SHFM1*, *SIRT1*, *TDP1*, *TDP2*, *TOPBP1*, *UIMC1*, *WRN*, *XRCC1* and *XRCC2*) and neurogenesis (particularly *APC*, *AREG*, *BMP2*, *CASP3, CAV1*, *CTNNB1*, *EZR*, *FN1*, *HGF*, *HSP90AA1*, *IFNG*, *IGF1*, *ITGA1*, *ITGB3*, *MT-3*, *MYCN*, *MYH9*, *MYO10*, *NRP2*, *PROM1*, *PTEN*, *RELA*, *RHOA*, *ROCK1*, *SOD1*, *TGFB1*, *TGFB2*, *TP53*, *VEGFA* and *XRCC2*), respectively. Moreover, we also constructed the interactome network (Figure 4B), where the nodes highlight the up-regulated genes involved in metal ion transport, vesicle-mediated transport and exocytosis (particularly *ABCB1*, *ABCB5*, *ACTN1*, *ATG3*, *ATG4A*, *ATG9A*, *BAK1*, *BCL2L1*, *CAV1*, *CAV2*, *CD274*, *CDC42*, *CDKN1B*, *CTNNB1*, *EGF*, *EZR*, *FGF2*, *FN1*, *GLS2*, *HGF*, *HSP90AA1*, *IFNG*, *IGF1*, *IGF2*, *ITGB3*, *MT-3*, *MYH10*, *MYH9*, *MYO10*, *MYO18A*, *MYO1B*, *PDGFA*, *PIK3CA*, *RAC1*, *SLC16A1*, *SLC16A4*, *SLC30A1*, *SLC30A5* and *SLC*). It is noteworthy that both networks display a pronouncedly high level of connectivity and dependence of annotated molecular pathways. This phenomenon highlights an importance of their mutual activity for the CDDP chemoresistance of Nbl cells.

### 3.4. Lysosomotropic Agents Re-Sensitize UKF-NB-4^CDDP^ Cells to CDDP

**Figure 5:**
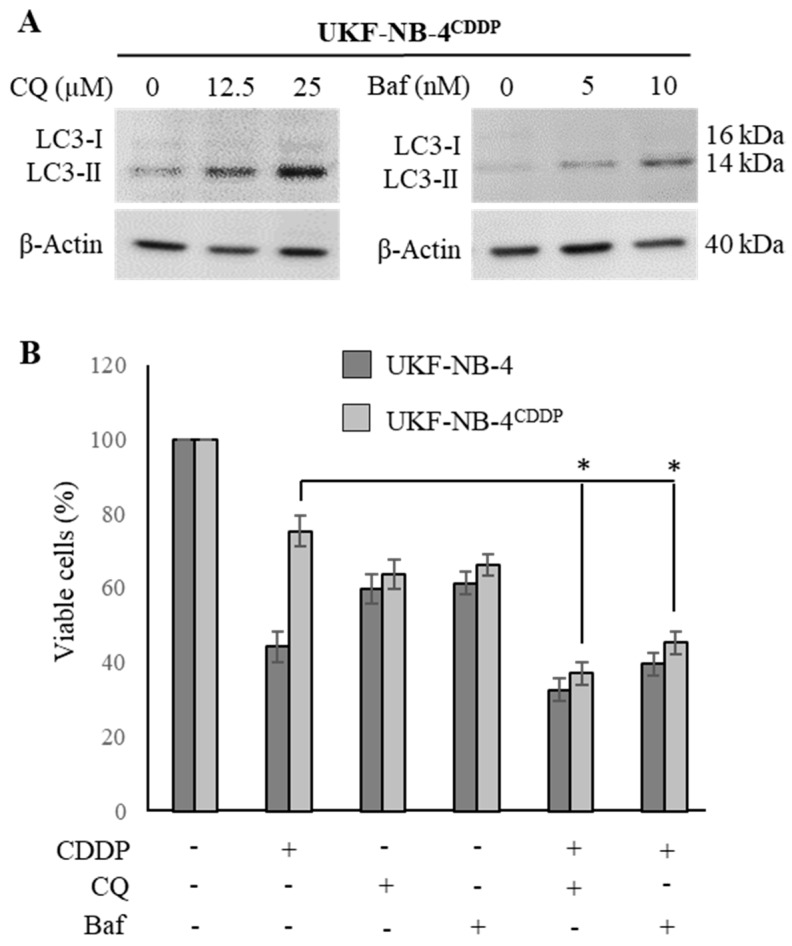
Re-sensitizing of UKF-NB-4^CDDP^ cells by lysosomotropic agents chloroquine (CQ) and Baf. (**A**) Whole-cell lysates immunoblots demonstrating conversion of LC3-I to LC3-II as a result inhibition of autophagy causing intracellular accumulation of autophagosomes. β-Actin–loading control. (**B**) Trypan blue exclusion data showing the re-sensitizing activity of CQ and Baf in UKF-NB-4^CDDP^ cells. The data are results from five independent experiments. * *p* < 0.01 related to CDDP cytotoxicity. +/− indicates the presence of CDDP, CQ or Baf in the treatment.

To validate the microarray data obtained, we finally performed a comparative analysis of cytotoxicity of CDDP and its co-administration with lysosomotropic agents CQ and Baf. First, we re-validated the concentrations of CQ and Baf that have stimulatory activity on conversion of LC3-I to LC3-II (Figure 5A). We found that 25 µM CQ and 10 nM Baf cause a successful inhibition of autophagy, resulting in intracellular accumulation of autophagosomes evidenced by an increased expression of LC3-II. Further, Figure 5B illustrates that both tested agents succeeded in re-sensitizing UKF-NB-4^CDDP^ cells to CDDP, while the highest synergistic effect was found for CQ. It is noteworthy that while applying CQ and Baf, the cytotoxic response of UKF-NB-4^CDDP^ cells is comparable to the effect identified for parental UKF-NB-4 cells. Taken together, these data indicate that the co-administration of CDDP with the above described inhibitors might serve as a novel tool for Nbl combination therapy. However, it must be noted that additional analyses using pre-clinical models might follow.

## 4. Discussion

The main challenge in treating Nbl, a pediatric cancer of the sympathetic nervous system, is to suppress metastasis appearance and resistance to multiple chemotherapeutic drugs formation. Multi-agent chemotherapy, including CDDP, is the conventional therapy for patients having advanced stages of Nbl [1,32]. In the present work, a comparative transcriptomic study was carried out to identify genes associated with CDDP resistance in cells derived from human high-risk Nbl. Such an approach allows us to gain deeper insights into the complex mechanism of the acquired resistance, which is a frequent issue specific for high-risk Nbl. This phenomenon worsens the prognosis of Nbl patients resulting in poor outcomes of therapeutic protocols [33,34,35,36,37]. Thus, the description of molecular pathways responsible for CDDP chemoresistance could result in the development of novel therapeutic strategies. The present study addresses these complex questions, which are the cellular processes that parental, sensitive Nbl cells acquire to achieve tolerance to CDDP. 

**Figure 6:**
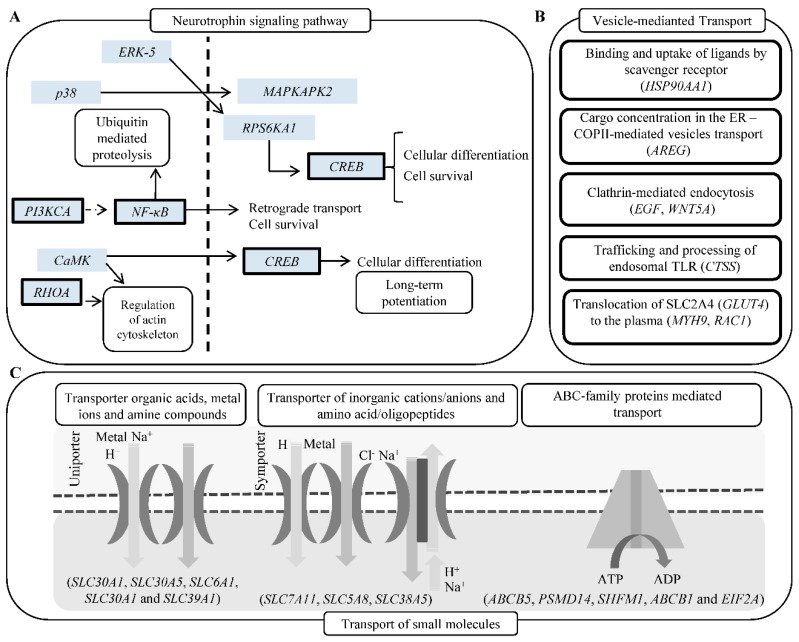
(**A**) Schematic depiction of genes (bold borders) up-regulated in UKF-NB-4^CDDP^ cells and involved in neurogenesis. (**B**) Schematic depiction of vesicles-mediated transport mechanisms with the list of genes up-regulated in UKF-NB-4^CDDP^ cells. (**C**) Schematic depiction of transport of small molecules, SLC-mediated transmembrane transport and ABC-family proteins mediated transport. Listed genes were identified as up-regulated in UKF-NB-4^CDDP^ cells. Data were analyzed by Reactome software.

In particular, our study suggests that CDDP chemoresistance can result from complex changes at molecular and cellular levels including reduced accumulation of the CDDP by either active efflux/sequestration/secretion or impaired influx, detoxification of CDDP by MTs and other antioxidants, increased activity of DNA damage repair (nucleotide excision repair and mismatch repair), alterations of membrane protein trafficking, regulated expression of microRNA, transcription factors and small GTPases and inactivation of the apoptotic pathways [15,22,38], which is schematically depicted in Figure 6A–C.

One of the generally accepted mechanisms of resistance to platinum-based cytostatics is connected with the up-regulation of distinct sub/isoforms of MTs, whose physiological role is to maintain the homeostasis of essential metals and protect against free radicals and toxic metals derivatives [39,40]. It was previously shown that MT has the ability to effectively bind and sequester CDDP [41]. Indeed, in the present study, we found that acquired chemoresistance to CDDP is associated with pronounced up-regulation of MT-3, which has an increasing significance as a diagnostic marker of various cancers and its presence frequently correlates with the poor chemotherapy outcome [22]. 

When free metal ions exceed the buffering capacity of MTs, they are being eliminated by ZnT-1 (encoded by SLC30A1) exporter [42]. It is worth noting that UKF-NB-4^CDDP^ cells exhibited up-regulation of a whole spectrum of metal ion transporters (SLC30A1, SLC30A5, SLC30A7, MT3 and MT1F). Therefore, one of the plausible mechanisms standing behind the acquired chemoresistance to CDDP comprises a stable up-regulation of these transporters to actively bind, sequester and export CDDP from the intracellular region [43]. Moreover, MT-3 has been connected with the regulation of lysosomal enrichment in neurons and astrocytes, [44,45], and it is therefore plausible that it can influence these processes also in MT-3 expressing cancer cells.

In line with aforementioned facts, it should be noted that during tumor formation, autophagy activation inhibits tumor growth. However, in solid tumors, autophagy promotes tumor cell survival by mitigating the consequences of stress [46]. Considering the fact that a large number of genes up-regulated in UKF-NB-4^CDDP^ cells are involved in a vesicle-mediated transport and exocytosis, we provide another piece of puzzle evidencing that lysosomal enrichment and sequestration is responsible for CDDP chemoresistance of UKF-NB-4 cells. This phenomenon was also functionally validated in vitro using two lysosomotropic agents (CQ and Baf) that inhibit autophagic flux by decreasing autophagosome-lysosome fusion [47]. Indeed, modulation of autophagosome-lysosome functions has been identified as a potent way to restore the sensitivity of cancer cells to distinct cytostatic agents [48,49,50], nevertheless, to the best of our knowledge, this is the first report indicating a suitability of CQ/Baf-CDDP combination therapy for the management of high-risk Nbl. 

The role of DNA repair in CDDP-resistant tumors has been extensively studied over the past years. Several genes regulating DNA damage, apoptosis and survival signaling is known to contribute to such chemoresistance [51]. In line with these data, we found that UKF-NB-4^CDDP^ cells up-regulate an array of genes encoding proteins responsible for DNA repair. Generally, cancer cells with acquired CDDP resistance demonstrate an enhanced capability to repair CDDP-induced DNA lesions or to tolerate a high level of unrepaired DNA lesions compared with CDDP-sensitive counterparts [52,53,54]. Interestingly, we found that both *BRCA1* and *BRCA2*, involved in repairing of double-strand DNA breaks via the homologous recombination, but also in nucleotide excision repairing, non-homologous end joining and activation of checkpoint responses [55], were up-regulated due to CDDP chemoresistance. 

Overall, we provide a comprehensive transcriptomic landscape of human high-risk Nbl cells with induced chemoresistance to CDDP. Our data demonstrate that the cells are capable of acquiring an array of molecular mechanisms to avoid cytotoxic effects of CDDP. Moreover, as suggested by the analysis of public cohort dataset, we provide a number of genes that might be further studied as prognostic biomarkers of an outcome of CDDP chemotherapy. Future investigations will be directed at deep proteomic signatures of UKF-NB-4^CDDP^ cells to identify possible druggable targets or prognostic biomarkers at the protein level.

## 5. Conclusions

In conclusion, our data strongly suggest that the major pathways behind the CDDP chemoresistance of Nbl cells are responsible for metal ions transport, vesicle-mediated transport and DNA repair. We provide an exceptional insight into the multifaceted nature of cancer chemoresistance that is a brilliant example of the adaptability of cancer cells to the unfavorable forces of external environment. From the perspective of future utilization of our data, we anticipate that several identified genes could serve as prognostic biomarkers of the outcome of CDDP therapy. Moreover, de-regulated genes could be further studied by functional analyses to reveal their specific role in Nbl pathogenesis. This could bring new druggable targets and novel possibilities for Nbl therapy.

## Figures and Tables

**Table 1:** List of genes up-regulated in UKF-NB-4^CDDP^ cells compared with parental UKF-NB-4 cell line.

UKF-NB-4^CDDP^ vs. UKF-NB-4				
**Up-Regulated**				
**Gene**	**Symbol**	**Fold Ratio**	**SD** **(*n* = 4)**	***p*** **Value**
26S proteasome complex subunit SEM1	*SHFM1*	2.88	0.36	0.009
26S proteasome non-ATPase regulatory subunit 14	*PSMD14*	2.39	0.12	0.007
Caveolin-2	*CAV2*	2.36	0.25	0.036
Transforming growth factor beta-2	*TGFB2*	2.34	0.11	0.003
Unconventional myosin-Ib	*MYO1B*	2.32	0.07	0.003
Retinal dehydrogenase 1	*ALDH1A1*	2.26	0.18	0.008
Zinc transporter 7	*SLC30A7*	2.14	0.43	0.018
Deoxycytidine kinase	*DCK*	2.06	0.17	0.002
DNA-dependent protein kinase catalytic subunit	*PRKDC*	2.05	0.07	0.025
Zinc transporter 1	*SLC30A1*	2.04	0.14	0.049
Autophagy-related protein 9A	*ATG9A*	2.04	0.04	0.013
Werner syndrome ATP-dependent helicase	*WRN*	2.02	0.12	0.018
Insulin-like growth factor I	*IGF1*	2.01	0.06	0.003
Breast cancer type 2 susceptibility protein	*BRCA2*	1.98	0.15	0.003
Monocarboxylate transporter 5	*SLC16A4*	1.98	0.21	0.008
Early activation antigen CD69	*CD69*	1.97	0.28	0.037
Myosin-10	*MYH10*	1.96	0.07	0.004
Bcl-2 homologous antagonist/killer	*BAK1*	1.96	0.09	0.035
E3 ubiquitin-protein ligase XIAP	*XIAP*	1.94	0.07	0.005
ATP-binding cassette sub-family B member 5	*ABCB5*	1.94	0.08	0.021
Checkpoint protein HUS1	*HUS1*	1.92	0.07	0.006
TIP41-like protein	*TIPRL*	1.92	0.10	0.009
Phospholipase D1	*PLD1*	1.92	0.31	0.003
Indoleamine 2,3-dioxygenase 1	*IDO1*	1.91	0.09	0.009
Transforming protein RhoA	*RHOA*	1.91	0.06	0.006
Phosphatidylinositol 3,4,5-trisphosphate 3-phosphatase and dual-specificity protein phosphatase PTEN	*PTEN*	1.91	0.09	0.005
Ezrin	*EZR*	1.90	0.06	0.001
Cadherin-2	*CDH2*	1.89	0.07	0.031
Crossover junction endonuclease MUS81	*MUS81*	1.88	0.15	0.019
Intercellular adhesion molecule 1	*ICAM1*	1.87	0.06	0.025
Phosphatidylinositol 4,5-bisphosphate 3-kinase catalytic subunit alpha isoform	*PIK3CA*	1.85	0.19	0.021
Cyclin-dependent kinase inhibitor 1B	*CDKN1B*	1.84	0.08	0.002
Metal regulatory transcription factor 1	*MTF1*	1.84	0.05	0.004
Fibronectin	*FN1*	1.84	0.08	0.008
Bifunctional methylenetetrahydrofolate dehydrogenase/cyclohydrolase, mitochondrial	*MTHFD2*	1.83	0.04	0.006
Cathepsin S	*CTSS*	1.81	0.06	0.013
Platelet-derived growth factor subunit A	*PDGFA*	1.81	0.07	0.045
Fibroblast growth factor 2	*FGF2*	1.81	0.06	0.012
DNA repair protein XRCC2	*XRCC2*	1.80	0.10	0.003
Flap endonuclease GEN homolog 1	*GEN1*	1.80	0.09	0.004
Pro-epidermal growth factor	*EGF*	1.80	0.07	0.001
NAD-dependent protein deacylase sirtuin-5, mitochondrial	*SIRT5*	1.77	0.04	0.005
Chemokine-like receptor 1	*CMKLR1*	1.77	0.06	0.021
Interferon gamma	*IFNG*	1.77	0.06	0.039
Cysteine protease ATG4A	*ATG4A*	1.77	0.16	0.044
Prolyl endopeptidase FAP	*FAP*	1.77	0.06	0.021
Protein Wnt-5a	*WNT5A*	1.76	0.09	0.004
Myosin-9	*MYH9*	1.75	0.06	0.001
Gem-associated protein 2	*GEMIN2*	1.75	0.07	0.009
Eukaryotic translation initiation factor 2-alpha kinase 3	*EIF2AK3*	1.74	0.08	0.004
NKG2-E type II integral membrane protein	*KLRC3*	1.74	0.07	0.009
3-hydroxy-3-methylglutaryl-coenzyme A reductase	*HMGCR*	1.73	0.05	0.007
Cystine/glutamate transporter	*SLC7A11*	1.73	0.10	0.036
DNA-directed RNA polymerase I subunit RPA1	*RPA1*	1.72	0.07	0.002
Multidrug resistance protein 1	*ABCB1*	1.72	0.13	0.040
Argininosuccinate synthase	*ASS1*	1.72	0.06	0.021
Interleukin-6 receptor subunit alpha	*IL6R*	1.71	0.06	0.006
Replication protein A 14 kDa subunit	*RPA3*	1.71	0.06	0.003
Tyrosyl-DNA phosphodiesterase 2	*TDP2*	1.71	0.10	0.018
Neuropilin-2	*NRP2*	1.70	0.07	0.007
Bcl-2-like protein 1	*BCL2L1*	1.70	0.03	0.005
Breast cancer type 1 susceptibility protein	*BRCA1*	1.70	0.10	0.025
NACHT, LRR and PYD domains-containing protein 2	*NLRP2*	1.69	0.09	0.024
DNA topoisomerase 2-binding protein 1	*TOPBP1*	1.69	0.08	0.047
Tumor necrosis factor receptor superfamily member 10B	*TNFRSF10B*	1.69	0.02	0.003
Activating molecule in BECN1-regulated autophagy protein 1	*AMBRA1*	1.68	0.06	0.005
78 kDa glucose-regulated protein	*HSPA5*	1.67	0.07	0.004
Tissue factor pathway inhibitor 2	*TFPI2*	1.67	0.09	0.047
Arginase-1	*ARG1*	1.66	0.06	0.025
Myosin light chain kinase, smooth muscle	*MYLK*	1.66	0.09	0.003
Bone morphogenetic protein 2	*BMP2*	1.66	0.05	0.007
E3 ubiquitin-protein ligase RNF8	*RNF8*	1.66	0.07	0.001
Alpha-actinin-3	*ACTN3*	1.65	0.05	0.003
Protein artemis	*DCLRE1C*	1.65	0.12	0.047
Monocarboxylate transporter 1	*SLC16A1*	1.65	0.06	0.014
CASP8 and FADD-like apoptosis regulator	*CFLAR*	1.65	0.06	0.003
Nibrin	*NBN*	1.65	0.13	0.051
Angiopoietin-1 receptor	*TEK*	1.65	0.06	0.017
Ras-related C3 botulinum toxin substrate 1	*RAC1*	1.64	0.03	0.021
BCL2/adenovirus E1B 19 kDa protein-interacting protein 3	*BNIP3*	1.64	0.10	0.008
BRCA1-A complex subunit RAP80	*UIMC1*	1.63	0.08	0.003
DNA damage-regulated autophagy modulator protein 2	*DRAM2*	1.63	0.07	0.041
Caveolin-1	*CAV1*	1.62	0.12	0.025
Ubiquitin-like-conjugating enzyme ATG3	*ATG3*	1.62	0.04	0.041
Cellular tumor antigen p53	*TP53*	1.62	0.05	0.006
Zinc finger E-box-binding homeobox 2	*ZEB2*	1.62	0.06	0.023
N-myc proto-oncogene protein	*MYCN*	1.61	0.12	0.001
Acetyl-coenzyme A synthetase, cytoplasmic	*ACSS2*	1.61	0.04	0.003
Superoxide dismutase [Cu-Zn]	*SOD1*	1.60	0.09	0.004
Sodium-coupled monocarboxylate transporter 1	*SLC5A8*	1.60	0.03	0.012
Nuclear factor NF-kappa-B p105 subunit	*NFKB1*	1.59	0.07	0.016
Zinc transporter ZIP1	*SLC39A1*	1.59	0.06	0.018
Transcription factor p65	*RELA*	1.59	0.06	0.002
Polycomb complex protein BMI-1	*BMI1*	1.59	0.10	0.003
Arginase-2, mitochondrial	*ARG2*	1.58	0.04	0.032
Cell division control protein 42 homolog	*CDC42*	1.58	0.05	0.061
Cyclic AMP-dependent transcription factor ATF-4	*ATF4*	1.58	0.06	0.014
Transforming growth factor beta-3	*TGFB3*	1.58	0.04	0.024
Glutaminase liver isoform, mitochondrial	*GLS2*	1.57	0.03	0.002
Cyclin-dependent kinase inhibitor 2A	*CDKN2A*	1.57	0.05	0.023
Tyrosyl-DNA phosphodiesterase 1	*TDP1*	1.57	0.09	0.041
Aurora kinase B	*AURKB*	1.57	0.07	0.025
Heat shock protein HSP 90-alpha	*HSP90AA1*	1.57	0.07	0.003
Fumarate Hydratase	*FH*	1.57	0.02	0.009
Eukaryotic translation initiation factor 2 subunit 1	*EIF2A*	1.57	0.07	0.021
Tumor necrosis factor receptor superfamily member 10A	*TNFRSF10A*	1.57	0.06	0.014
Prominin-1	*PROM1*	1.56	0.08	0.009
Insulin-like growth factor-binding protein 4	*IGFBP4*	1.56	0.06	0.006
Unconventional myosin-X	*MYO10*	1.56	0.05	0.005
Rho-associated protein kinase 1	*ROCK1*	1.56	0.02	0.035
Microtubule-associated proteins 1A/1B light chain 3A	*MAP1LC3A*	1.56	0.06	0.004
DNA excision repair protein ERCC-1	*ERCC1*	1.56	0.07	0.003
Double-strand break repair protein MRE11	*MRE11A*	1.55	10.82	0.003
UDP-N-acetylglucosamine--peptide N-acetylglucosaminyltransferase 110 kDa subunit	*OGT*	1.55	0.03	0.020
GMP synthase [glutamine-hydrolyzing]	*GMPS*	1.55	0.06	0.002
Catenin beta-1	*CTNNB1*	1.55	0.05	0.001
Zinc transporter 5	*SLC30A5*	1.55	0.06	0.049
Homeobox protein NANOG	*NANOG*	1.55	0.13	0.027
BCL2 Associated X, Apoptosis Regulator	*BAX*	1.55	0.11	0.004
Integrin beta-3	*ITGB3*	1.55	0.08	0.009
Amphiregulin	*AREG*	1.54	0.06	0.005
DNA repair protein XRCC1	*XRCC1*	1.54	0.07	0.034
Metallothionein-3	*MT3*	1.54	0.04	0.008
Caspase-3	*CASP3*	1.54	0.03	0.020
Insulin-like growth factor II	*IGF2*	1.54	0.05	0.003
Integrin alpha-1	*ITGA1*	1.54	0.08	0.007
Hepatocyte growth factor	*HGF*	1.54	0.06	0.009
DNA damage-regulated autophagy modulator protein 1	*DRAM1*	1.53	0.04	0.020
Serine/threonine-protein kinase Chk2	*CHEK2*	1.53	0.10	0.018
Transforming growth factor beta-1	*TGFB1*	1.53	0.04	0.008
Programmed cell death 1 ligand 1	*PDL1*	1.53	0.07	0.006
Unconventional myosin-XVIIIa	*MYO18A*	1.53	0.07	0.004
Alpha-actinin-1	*ACTN1*	1.52	0.04	0.035
Growth arrest and DNA damage-inducible protein GADD45 alpha	*GADD45A*	1.52	0.05	0.004
Sodium-coupled neutral amino acid transporter 5	*SLC38A5*	1.51	0.05	0.037
Adenomatous polyposis coli protein	*APC*	1.51	0.08	0.049
Vascular endothelial growth factor A	*VEGFA*	1.51	0.06	0.008
Receptor tyrosine-protein kinase erbB-2	*ERBB2*	1.50	0.05	0.003
NAD-dependent protein deacetylase sirtuin-1	*SIRT1*	1.50	0.05	0.003
**Down-Regulated**				
**Gene**	**Symbol**	**Fold Ratio**	**SD** **(*n* = 6)**	***p*** **Value**
Caspase 8	*CASP8*	2.55	0.09	0.018
Suppressor of cytokine signaling 1	*SOCS1*	1.52	0.01	0.025
Desmocollin-2	*DSC2*	2.20	0.08	0.047
AHNAK Nucleoprotein	*AHNAK*	1.95	0.05	0.040

## Supplementary Materials

The following are available online at https://www.mdpi.com/2073-4409/8/3/235/s1, Figure S1: Representative microarray heatmaps showing gene expression in UKF-NB-4 and UKF-NB-4^CDDP^ cells (one spot per one gene), Table S1: The full list of genes within microarray chips utilized in this study, Table S2: List of primers employed for validation of selected microarray results using qPCR.
