# Supplementary material for: Transcriptomic Landscape of Cisplatin-Resistant Neuroblastoma Cells
Source: Cells. 2019 Mar 12;8(3):235. doi: 10.3390/cells8030235 (PMC6469049; doi:10.3390/cells8030235)
Supplement: Supplementary file 1 [file cells-08-00235-s001.zip › Supplementary Figure 1_R1.docx]

**Supplementary Figure 1.** Representative microarray heatmaps showing gene expression in UKF-NB-4 and UKF-NB-4CDDP cells (one spot per one gene). Gray scale intensity represents the rate of individual mRNA expression.

**UKF-NB-4**


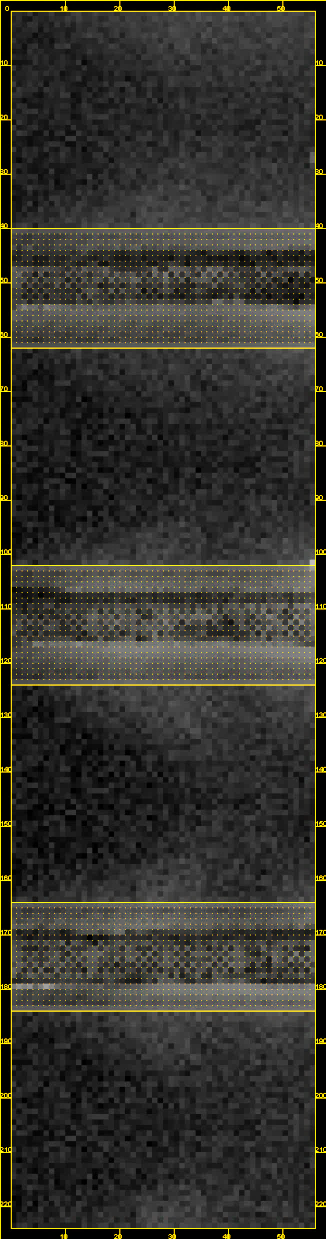


Inter-place zone of no hybridization

Inter-place zone of no hybridization

Inter-place zone of no hybridization

**UKF-NB-4**

**UKF-NB-4^CDDP^**

**UKF-NB-4^CDDP^**
