# Supplementary material for: Transcriptomic Landscape of Cisplatin-Resistant Neuroblastoma Cells
Source: Cells. 2019 Mar 12;8(3):235. doi: 10.3390/cells8030235 (PMC6469049; doi:10.3390/cells8030235)
Supplement: Supplementary file 1 [file cells-08-00235-s001.zip › Supplementary Table 1_R1.docx]

**Summplementary table S1:** The full list of genes within microarray chips utilized in this study.

| # | Name | Length | Origin |
| --- | --- | --- | --- |
| 1 | NC-bacteria-9-11 | 35 | factory |
| 2 | NC-bacteria-9-9 | 35 | factory |
| 3 | NC-bacteria-5-11 | 35 | factory |
| 4 | NC-bacteria-10-7 | 35 | factory |
| 5 | NC-bacteria-10-5 | 35 | factory |
| 6 | NC-bacteria-9-4 | 35 | factory |
| 7 | NC-bacteria-5-4 | 35 | factory |
| 8 | NC-bacteria-5-12 | 35 | factory |
| 9 | QC-ES-oligo-BioB4-AS2 | 35 | factory |
| 10 | QC-ES-WT1-15 | 35 | factory |
| 11 | QC-ES-WT2-16 | 35 | factory |
| 12 | QC-ES-WT5-20 | 35 | factory |
| 13 | QC-ES-WT8-23 | 35 | factory |
| 14 | QC-ES-WT10-25 | 35 | factory |
| 15 | QC-ES-WT13-28 | 35 | factory |
| 16 | QC-ES-WT17-32 | 35 | factory |
| 17 | QC-ES-WT20-35 | 35 | factory |
| 18 | QC-ES-1SNP | 35 | factory |
| 19 | QC-ES-2SNP | 35 | factory |
| 20 | QC-ES-3SNP | 35 | factory |
| 21 | Gene.ABCB1\|NM_000927\|4718\|1406-1366 | 41 | imported |
| 22 | Gene.ABCB1\|NM_000927\|4718\|1366-1406 | 41 | imported |
| 23 | Gene.ABCB1\|NM_000927\|4718\|2924-2887 | 38 | imported |
| 24 | Gene.ABCB1\|NM_000927\|4718\|2887-2924 | 38 | imported |
| 25 | Gene.ABCB1\|NM_000927\|4718\|3199-3160 | 40 | imported |
| 26 | Gene.ABCB1\|NM_000927\|4718\|3160-3199 | 40 | imported |
| 27 | Gene.ABCB5\|NM_001163941\|5401\|1393-1351 | 43 | imported |
| 28 | Gene.ABCB5\|NM_001163941\|5401\|1351-1393 | 43 | imported |
| 29 | Gene.ABCB5\|NM_001163942\|2247\|242-210 | 33 | imported |
| 30 | Gene.ABCB5\|NM_001163942\|2247\|210-242 | 33 | imported |
| 31 | Gene.ABCB5\|NM_178559\|4375\|811-778 | 34 | imported |
| 32 | Gene.ABCB5\|NM_178559\|4375\|778-811 | 34 | imported |
| 33 | Gene.ACLY\|NM_001096\|4450\|65-38 | 28 | imported |
| 34 | Gene.ACLY\|NM_001096\|4450\|38-65 | 28 | imported |
| 35 | Gene.ACLY\|NM_001096\|4450\|2027-1999 | 29 | imported |
| 36 | Gene.ACLY\|NM_001096\|4450\|1999-2027 | 29 | imported |
| 37 | Gene.ACLY\|NM_001096\|4450\|2102-2073 | 30 | imported |
| 38 | Gene.ACLY\|NM_001096\|4450\|2073-2102 | 30 | imported |
| 39 | Gene.ACSS1\|NM_001252676\|3519\|3450-3423 | 28 | imported |
| 40 | Gene.ACSS1\|NM_001252676\|3519\|3423-3450 | 28 | imported |
| 41 | Gene.ACSS1\|NM_001252676\|3519\|3496-3452 | 45 | imported |
| 42 | Gene.ACSS1\|NM_001252676\|3519\|3452-3496 | 45 | imported |
| 43 | Gene.ACSS1\|NM_001252675\|3686\|3070-3039 | 32 | imported |
| 44 | Gene.ACSS1\|NM_001252675\|3686\|3039-3070 | 32 | imported |
| 45 | Gene.ACSS2\|NM_001076552\|3027\|1257-1226 | 32 | imported |
| 46 | Gene.ACSS2\|NM_001076552\|3027\|1226-1257 | 32 | imported |
| 47 | Gene.ACSS2\|NM_001076552\|3027\|1953-1926 | 28 | imported |
| 48 | Gene.ACSS2\|NM_001076552\|3027\|1926-1953 | 28 | imported |
| 49 | Gene.ACSS2\|NM_001076552\|3027\|2031-2001 | 31 | imported |
| 50 | Gene.ACSS2\|NM_001076552\|3027\|2001-2031 | 31 | imported |
| 51 | Gene.ACTA2\|NM_001141945\|1805\|663-631 | 33 | imported |
| 52 | Gene.ACTA2\|NM_001141945\|1805\|631-663 | 33 | imported |
| 53 | Gene.ACTA2\|NM_001141945\|1805\|675-646 | 30 | imported |
| 54 | Gene.ACTA2\|NM_001141945\|1805\|646-675 | 30 | imported |
| 55 | Gene.ACTA2\|NM_001141945\|1805\|481-451 | 31 | imported |
| 56 | Gene.ACTA2\|NM_001141945\|1805\|451-481 | 31 | imported |
| 57 | Gene.ACTB\|NM_001101\|1852\|779-753 | 27 | imported |
| 58 | Gene.ACTB\|NM_001101\|1852\|753-779 | 27 | imported |
| 59 | Gene.ACTB\|NM_001101\|1852\|681-657 | 25 | imported |
| 60 | Gene.ACTB\|NM_001101\|1852\|657-681 | 25 | imported |
| 61 | Gene.ACTB\|NM_001101\|1852\|764-737 | 28 | imported |
| 62 | Gene.ACTB\|NM_001101\|1852\|737-764 | 28 | imported |
| 63 | Gene.ACTB\|NM_001101\|1852\|513-481 | 33 | imported |
| 64 | Gene.ACTB\|NM_001101\|1852\|481-513 | 33 | imported |
| 65 | Gene.ACTB\|NM_001101\|1852\|793-769 | 25 | imported |
| 66 | Gene.ACTB\|NM_001101\|1852\|769-793 | 25 | imported |
| 67 | Gene.ACTN1\|NM_001102\|3743\|1861-1830 | 32 | imported |
| 68 | Gene.ACTN1\|NM_001102\|3743\|1830-1861 | 32 | imported |
| 69 | Gene.ACTN1\|NM_001102\|3743\|2536-2512 | 25 | imported |
| 70 | Gene.ACTN1\|NM_001102\|3743\|2512-2536 | 25 | imported |
| 71 | Gene.ACTN1\|NM_001130004\|3809\|2171-2145 | 27 | imported |
| 72 | Gene.ACTN1\|NM_001130004\|3809\|2145-2171 | 27 | imported |
| 73 | Gene.ACTN3\|NM_001104\|2952\|604-576 | 29 | imported |
| 74 | Gene.ACTN3\|NM_001104\|2952\|576-604 | 29 | imported |
| 75 | Gene.ACTN3\|NM_001104\|2952\|1978-1951 | 28 | imported |
| 76 | Gene.ACTN3\|NM_001104\|2952\|1951-1978 | 28 | imported |
| 77 | Gene.ACTN3\|NM_001104\|2952\|2935-2901 | 35 | imported |
| 78 | Gene.ACTN3\|NM_001104\|2952\|2901-2935 | 35 | imported |
| 79 | Gene.ACTR2\|NM_001005386\|3944\|500-463 | 38 | imported |
| 80 | Gene.ACTR2\|NM_001005386\|3944\|463-500 | 38 | imported |
| 81 | Gene.ACTR2\|NM_001005386\|3944\|1388-1354 | 35 | imported |
| 82 | Gene.ACTR2\|NM_001005386\|3944\|1354-1388 | 35 | imported |
| 83 | Gene.ACTR2\|NM_005722\|3929\|1422-1387 | 36 | imported |
| 84 | Gene.ACTR2\|NM_005722\|3929\|1387-1422 | 36 | imported |
| 85 | Gene.ACTR3\|NM_001277140\|5499\|727-691 | 37 | imported |
| 86 | Gene.ACTR3\|NM_001277140\|5499\|691-727 | 37 | imported |
| 87 | Gene.ACTR3\|NM_001277140\|5499\|1137-1105 | 33 | imported |
| 88 | Gene.ACTR3\|NM_001277140\|5499\|1105-1137 | 33 | imported |
| 89 | Gene.ACTR3\|NM_005721\|5708\|516-481 | 36 | imported |
| 90 | Gene.ACTR3\|NM_005721\|5708\|481-516 | 36 | imported |
| 91 | Gene.ADAM17\|NM_003183\|4395\|584-556 | 29 | imported |
| 92 | Gene.ADAM17\|NM_003183\|4395\|556-584 | 29 | imported |
| 93 | Gene.ADAM17\|NM_003183\|4395\|1846-1814 | 33 | imported |
| 94 | Gene.ADAM17\|NM_003183\|4395\|1814-1846 | 33 | imported |
| 95 | Gene.ADAM17\|NM_003183\|4395\|2581-2554 | 28 | imported |
| 96 | Gene.ADAM17\|NM_003183\|4395\|2554-2581 | 28 | imported |
| 97 | Gene.ADGRG1\|NM_001198894\|3605\|1503-1471 | 33 | imported |
| 98 | Gene.ADGRG1\|NM_001198894\|3605\|1471-1503 | 33 | imported |
| 99 | Gene.ADGRG1\|NM_001198894\|3605\|1977-1951 | 27 | imported |
| 100 | Gene.ADGRG1\|NM_001198894\|3605\|1951-1977 | 27 | imported |
| 101 | Gene.ADGRG1\|NM_018882\|3555\|1347-1321 | 27 | imported |
| 102 | Gene.ADGRG1\|NM_018882\|3555\|1321-1347 | 27 | imported |
| 103 | Gene.AHNAK\|NM_001620\|18836\|502-472 | 31 | imported |
| 104 | Gene.AHNAK\|NM_001620\|18836\|472-502 | 31 | imported |
| 105 | Gene.AHNAK\|NM_024060\|1096\|286-261 | 26 | imported |
| 106 | Gene.AHNAK\|NM_024060\|1096\|261-286 | 26 | imported |
| 107 | Gene.AHNAK\|NM_024060\|1096\|620-591 | 30 | imported |
| 108 | Gene.AHNAK\|NM_024060\|1096\|591-620 | 30 | imported |
| 109 | Gene.AKT1\|NM_001014431\|2794\|465-438 | 28 | imported |
| 110 | Gene.AKT1\|NM_001014431\|2794\|438-465 | 28 | imported |
| 111 | Gene.AKT1\|NM_001014431\|2794\|671-645 | 27 | imported |
| 112 | Gene.AKT1\|NM_001014431\|2794\|645-671 | 27 | imported |
| 113 | Gene.AKT1\|NM_001014431\|2794\|1911-1887 | 25 | imported |
| 114 | Gene.AKT1\|NM_001014431\|2794\|1887-1911 | 25 | imported |
| 115 | Gene.ALCAM\|NM_001243280\|4884\|943-903 | 41 | imported |
| 116 | Gene.ALCAM\|NM_001243280\|4884\|903-943 | 41 | imported |
| 117 | Gene.ALCAM\|NM_001243281\|2961\|303-276 | 28 | imported |
| 118 | Gene.ALCAM\|NM_001243281\|2961\|276-303 | 28 | imported |
| 119 | Gene.ALCAM\|NM_001243283\|2075\|1058-1021 | 38 | imported |
| 120 | Gene.ALCAM\|NM_001243283\|2075\|1021-1058 | 38 | imported |
| 121 | Gene.ALDH1A1\|NM_000689\|2378\|716-681 | 36 | imported |
| 122 | Gene.ALDH1A1\|NM_000689\|2378\|681-716 | 36 | imported |
| 123 | Gene.ALDH1A1\|NM_000689\|2378\|1032-1001 | 32 | imported |
| 124 | Gene.ALDH1A1\|NM_000689\|2378\|1001-1032 | 32 | imported |
| 125 | Gene.ALDH1A1\|NM_000689\|2378\|1625-1581 | 45 | imported |
| 126 | Gene.ALDH1A1\|NM_000689\|2378\|1581-1625 | 45 | imported |
| 127 | Gene.ALDH1A3\|NM_000693\|3622\|747-721 | 27 | imported |
| 128 | Gene.ALDH1A3\|NM_000693\|3622\|721-747 | 27 | imported |
| 129 | Gene.ALDH1A3\|NM_000693\|3622\|3345-3301 | 45 | imported |
| 130 | Gene.ALDH1A3\|NM_000693\|3622\|3301-3345 | 45 | imported |
| 131 | Gene.ALDH1A3\|NM_000693\|3622\|3569-3541 | 29 | imported |
| 132 | Gene.ALDH1A3\|NM_000693\|3622\|3541-3569 | 29 | imported |
| 133 | Gene.AMBRA1\|NM_001267782\|5315\|912-881 | 32 | imported |
| 134 | Gene.AMBRA1\|NM_001267782\|5315\|881-912 | 32 | imported |
| 135 | Gene.AMBRA1\|NM_001267782\|5315\|2979-2949 | 31 | imported |
| 136 | Gene.AMBRA1\|NM_001267782\|5315\|2949-2979 | 31 | imported |
| 137 | Gene.AMBRA1\|NM_001267782\|5315\|4652-4621 | 32 | imported |
| 138 | Gene.AMBRA1\|NM_001267782\|5315\|4621-4652 | 32 | imported |
| 139 | Gene.ANGPTL4\|NM_001039667\|1791\|80-46 | 35 | imported |
| 140 | Gene.ANGPTL4\|NM_001039667\|1791\|46-80 | 35 | imported |
| 141 | Gene.ANGPTL4\|NM_001039667\|1791\|956-931 | 26 | imported |
| 142 | Gene.ANGPTL4\|NM_001039667\|1791\|931-956 | 26 | imported |
| 143 | Gene.ANGPTL4\|NM_001039667\|1791\|1137-1111 | 27 | imported |
| 144 | Gene.ANGPTL4\|NM_001039667\|1791\|1111-1137 | 27 | imported |
| 145 | Gene.ANPEP\|NM_001150\|3740\|2140-2109 | 32 | imported |
| 146 | Gene.ANPEP\|NM_001150\|3740\|2109-2140 | 32 | imported |
| 147 | Gene.ANPEP\|NM_001150\|3740\|2421-2388 | 34 | imported |
| 148 | Gene.ANPEP\|NM_001150\|3740\|2388-2421 | 34 | imported |
| 149 | Gene.ANPEP\|NM_001150\|3740\|2522-2481 | 42 | imported |
| 150 | Gene.ANPEP\|NM_001150\|3740\|2481-2522 | 42 | imported |
| 151 | Gene.APC\|NM_000038\|10740\|9937-9901 | 37 | imported |
| 152 | Gene.APC\|NM_000038\|10740\|9901-9937 | 37 | imported |
| 153 | Gene.APC\|NM_001127510\|10848\|1217-1184 | 34 | imported |
| 154 | Gene.APC\|NM_001127510\|10848\|1184-1217 | 34 | imported |
| 155 | Gene.APC\|NM_001127510\|10848\|4490-4460 | 31 | imported |
| 156 | Gene.APC\|NM_001127510\|10848\|4460-4490 | 31 | imported |
| 157 | Gene.AREG\|NM_001657\|1290\|171-144 | 28 | imported |
| 158 | Gene.AREG\|NM_001657\|1290\|144-171 | 28 | imported |
| 159 | Gene.AREG\|NM_001657\|1290\|257-232 | 26 | imported |
| 160 | Gene.AREG\|NM_001657\|1290\|232-257 | 26 | imported |
| 161 | Gene.AREG\|NM_001657\|1290\|384-353 | 32 | imported |
| 162 | Gene.AREG\|NM_001657\|1290\|353-384 | 32 | imported |
| 163 | Gene.ARG1\|NM_000045\|1475\|517-481 | 37 | imported |
| 164 | Gene.ARG1\|NM_000045\|1475\|481-517 | 37 | imported |
| 165 | Gene.ARG1\|NM_000045\|1475\|811-781 | 31 | imported |
| 166 | Gene.ARG1\|NM_000045\|1475\|781-811 | 31 | imported |
| 167 | Gene.ARG1\|NM_001244438\|1499\|1067-1028 | 40 | imported |
| 168 | Gene.ARG1\|NM_001244438\|1499\|1028-1067 | 40 | imported |
| 169 | Gene.ARG2\|NM_001172\|1981\|115-86 | 30 | imported |
| 170 | Gene.ARG2\|NM_001172\|1981\|86-115 | 30 | imported |
| 171 | Gene.ARG2\|NM_001172\|1981\|266-239 | 28 | imported |
| 172 | Gene.ARG2\|NM_001172\|1981\|239-266 | 28 | imported |
| 173 | Gene.ARG2\|NM_001172\|1981\|304-273 | 32 | imported |
| 174 | Gene.ARG2\|NM_001172\|1981\|273-304 | 32 | imported |
| 175 | Gene.ASL\|NM_000048\|1937\|322-289 | 34 | imported |
| 176 | Gene.ASL\|NM_000048\|1937\|289-322 | 34 | imported |
| 177 | Gene.ASL\|NM_000048\|1937\|1184-1153 | 32 | imported |
| 178 | Gene.ASL\|NM_000048\|1937\|1153-1184 | 32 | imported |
| 179 | Gene.ASL\|NM_000048\|1937\|1372-1345 | 28 | imported |
| 180 | Gene.ASL\|NM_000048\|1937\|1345-1372 | 28 | imported |
| 181 | Gene.ASNS\|NM_001178075\|2010\|1182-1140 | 43 | imported |
| 182 | Gene.ASNS\|NM_001178075\|2010\|1140-1182 | 43 | imported |
| 183 | Gene.ASNS\|NM_001178075\|2010\|1427-1395 | 33 | imported |
| 184 | Gene.ASNS\|NM_001178075\|2010\|1395-1427 | 33 | imported |
| 185 | Gene.ASNS\|NM_001178076\|1776\|1460-1426 | 35 | imported |
| 186 | Gene.ASNS\|NM_001178076\|1776\|1426-1460 | 35 | imported |
| 187 | Gene.ASS1\|NM_000050\|1863\|448-417 | 32 | imported |
| 188 | Gene.ASS1\|NM_000050\|1863\|417-448 | 32 | imported |
| 189 | Gene.ASS1\|NM_000050\|1863\|577-545 | 33 | imported |
| 190 | Gene.ASS1\|NM_000050\|1863\|545-577 | 33 | imported |
| 191 | Gene.ASS1\|NM_000050\|1863\|665-641 | 25 | imported |
| 192 | Gene.ASS1\|NM_000050\|1863\|641-665 | 25 | imported |
| 193 | Gene.ATF4\|NM_001675\|2041\|46-18 | 29 | imported |
| 194 | Gene.ATF4\|NM_001675\|2041\|18-46 | 29 | imported |
| 195 | Gene.ATF4\|NM_001675\|2041\|30-1 | 30 | imported |
| 196 | Gene.ATF4\|NM_001675\|2041\|1-30 | 30 | imported |
| 197 | Gene.ATF4\|NM_001675\|2041\|1866-1837 | 30 | imported |
| 198 | Gene.ATF4\|NM_001675\|2041\|1837-1866 | 30 | imported |
| 199 | Gene.ATG10\|NM_001131028\|2432\|558-521 | 38 | imported |
| 200 | Gene.ATG10\|NM_001131028\|2432\|521-558 | 38 | imported |
| 201 | Gene.ATG10\|NM_001131028\|2432\|1056-1021 | 36 | imported |
| 202 | Gene.ATG10\|NM_001131028\|2432\|1021-1056 | 36 | imported |
| 203 | Gene.ATG10\|NM_001131028\|2432\|1865-1821 | 45 | imported |
| 204 | Gene.ATG10\|NM_001131028\|2432\|1821-1865 | 45 | imported |
| 205 | Gene.ATG16L1\|NM_001190266\|3409\|1458-1422 | 37 | imported |
| 206 | Gene.ATG16L1\|NM_001190266\|3409\|1422-1458 | 37 | imported |
| 207 | Gene.ATG16L1\|NM_001190266\|3409\|2208-2176 | 33 | imported |
| 208 | Gene.ATG16L1\|NM_001190266\|3409\|2176-2208 | 33 | imported |
| 209 | Gene.ATG16L1\|NM_198890\|2922\|1859-1825 | 35 | imported |
| 210 | Gene.ATG16L1\|NM_198890\|2922\|1825-1859 | 35 | imported |
| 211 | Gene.ATG16L2\|NM_001318766\|2280\|1280-1255 | 26 | imported |
| 212 | Gene.ATG16L2\|NM_001318766\|2280\|1255-1280 | 26 | imported |
| 213 | Gene.ATG16L2\|NM_033388\|2160\|730-703 | 28 | imported |
| 214 | Gene.ATG16L2\|NM_033388\|2160\|703-730 | 28 | imported |
| 215 | Gene.ATG16L2\|NM_033388\|2160\|1686-1657 | 30 | imported |
| 216 | Gene.ATG16L2\|NM_033388\|2160\|1657-1686 | 30 | imported |
| 217 | Gene.ATG3\|NM_001278712\|3060\|106-79 | 28 | imported |
| 218 | Gene.ATG3\|NM_001278712\|3060\|79-106 | 28 | imported |
| 219 | Gene.ATG3\|NM_001278712\|3060\|497-469 | 29 | imported |
| 220 | Gene.ATG3\|NM_001278712\|3060\|469-497 | 29 | imported |
| 221 | Gene.ATG3\|NM_022488\|1572\|404-378 | 27 | imported |
| 222 | Gene.ATG3\|NM_022488\|1572\|378-404 | 27 | imported |
| 223 | Gene.ATG4A\|NM_001321287\|3038\|2004-1976 | 29 | imported |
| 224 | Gene.ATG4A\|NM_001321287\|3038\|1976-2004 | 29 | imported |
| 225 | Gene.ATG4A\|NM_001321287\|3038\|2384-2351 | 34 | imported |
| 226 | Gene.ATG4A\|NM_001321287\|3038\|2351-2384 | 34 | imported |
| 227 | Gene.ATG4A\|NM_001321288\|2359\|2014-1981 | 34 | imported |
| 228 | Gene.ATG4A\|NM_001321288\|2359\|1981-2014 | 34 | imported |
| 229 | Gene.ATG5\|NM_001286106\|3125\|1811-1769 | 43 | imported |
| 230 | Gene.ATG5\|NM_001286106\|3125\|1769-1811 | 43 | imported |
| 231 | Gene.ATG5\|NM_001286107\|3132\|1776-1743 | 34 | imported |
| 232 | Gene.ATG5\|NM_001286107\|3132\|1743-1776 | 34 | imported |
| 233 | Gene.ATG5\|NM_001286107\|3132\|2151-2107 | 45 | imported |
| 234 | Gene.ATG5\|NM_001286107\|3132\|2107-2151 | 45 | imported |
| 235 | Gene.ATG7\|NM_001136031\|4978\|292-253 | 40 | imported |
| 236 | Gene.ATG7\|NM_001136031\|4978\|253-292 | 40 | imported |
| 237 | Gene.ATG7\|NM_001136031\|4978\|2716-2689 | 28 | imported |
| 238 | Gene.ATG7\|NM_001136031\|4978\|2689-2716 | 28 | imported |
| 239 | Gene.ATG7\|NM_001136031\|4978\|3017-2983 | 35 | imported |
| 240 | Gene.ATG7\|NM_001136031\|4978\|2983-3017 | 35 | imported |
| 241 | Gene.ATG9A\|NM_001077198\|3910\|430-397 | 34 | imported |
| 242 | Gene.ATG9A\|NM_001077198\|3910\|397-430 | 34 | imported |
| 243 | Gene.ATG9A\|NM_001077198\|3910\|2699-2674 | 26 | imported |
| 244 | Gene.ATG9A\|NM_001077198\|3910\|2674-2699 | 26 | imported |
| 245 | Gene.ATG9A\|NM_001077198\|3910\|3130-3103 | 28 | imported |
| 246 | Gene.ATG9A\|NM_001077198\|3910\|3103-3130 | 28 | imported |
| 247 | Gene.ATM\|NM_000051\|13147\|5419-5391 | 29 | imported |
| 248 | Gene.ATM\|NM_000051\|13147\|5391-5419 | 29 | imported |
| 249 | Gene.ATM\|NM_000051\|13147\|5543-5501 | 43 | imported |
| 250 | Gene.ATM\|NM_000051\|13147\|5501-5543 | 43 | imported |
| 251 | Gene.ATM\|NM_000051\|13147\|9497-9461 | 37 | imported |
| 252 | Gene.ATM\|NM_000051\|13147\|9461-9497 | 37 | imported |
| 253 | Gene.ATR\|NM_001184\|8258\|4382-4348 | 35 | imported |
| 254 | Gene.ATR\|NM_001184\|8258\|4348-4382 | 35 | imported |
| 255 | Gene.ATR\|NM_001184\|8258\|5828-5797 | 32 | imported |
| 256 | Gene.ATR\|NM_001184\|8258\|5797-5828 | 32 | imported |
| 257 | Gene.ATR\|NM_001184\|8258\|6033-6004 | 30 | imported |
| 258 | Gene.ATR\|NM_001184\|8258\|6004-6033 | 30 | imported |
| 259 | Gene.ATRIP\|NM_001271022\|2419\|831-801 | 31 | imported |
| 260 | Gene.ATRIP\|NM_001271022\|2419\|801-831 | 31 | imported |
| 261 | Gene.ATRIP\|NM_001271023\|2676\|2075-2047 | 29 | imported |
| 262 | Gene.ATRIP\|NM_001271023\|2676\|2047-2075 | 29 | imported |
| 263 | Gene.ATRIP\|NM_130384\|2629\|1655-1629 | 27 | imported |
| 264 | Gene.ATRIP\|NM_130384\|2629\|1629-1655 | 27 | imported |
| 265 | Gene.ATXN1\|NM_000332\|10636\|6701-6676 | 26 | imported |
| 266 | Gene.ATXN1\|NM_000332\|10636\|6676-6701 | 26 | imported |
| 267 | Gene.ATXN1\|NM_001128164\|10587\|7608-7569 | 40 | imported |
| 268 | Gene.ATXN1\|NM_001128164\|10587\|7569-7608 | 40 | imported |
| 269 | Gene.ATXN1\|NM_001128164\|10587\|9633-9593 | 41 | imported |
| 270 | Gene.ATXN1\|NM_001128164\|10587\|9593-9633 | 41 | imported |
| 271 | Gene.AURKB\|NM_001256834\|1241\|197-171 | 27 | imported |
| 272 | Gene.AURKB\|NM_001256834\|1241\|171-197 | 27 | imported |
| 273 | Gene.AURKB\|NM_001256834\|1241\|227-201 | 27 | imported |
| 274 | Gene.AURKB\|NM_001256834\|1241\|201-227 | 27 | imported |
| 275 | Gene.AURKB\|NM_001256834\|1241\|297-261 | 37 | imported |
| 276 | Gene.AURKB\|NM_001256834\|1241\|261-297 | 37 | imported |
| 277 | Gene.BAD\|NM_004322\|1240\|739-711 | 29 | imported |
| 278 | Gene.BAD\|NM_004322\|1240\|711-739 | 29 | imported |
| 279 | Gene.BAD\|NM_004322\|1240\|775-751 | 25 | imported |
| 280 | Gene.BAD\|NM_004322\|1240\|751-775 | 25 | imported |
| 281 | Gene.BAD\|NM_032989\|986\|307-281 | 27 | imported |
| 282 | Gene.BAD\|NM_032989\|986\|281-307 | 27 | imported |
| 283 | Gene.BAI1\|NM_001702\|5535\|1407-1381 | 27 | imported |
| 284 | Gene.BAI1\|NM_001702\|5535\|1381-1407 | 27 | imported |
| 285 | Gene.BAI1\|NM_001702\|5535\|2695-2669 | 27 | imported |
| 286 | Gene.BAI1\|NM_001702\|5535\|2669-2695 | 27 | imported |
| 287 | Gene.BAI1\|NM_001702\|5535\|4307-4279 | 29 | imported |
| 288 | Gene.BAI1\|NM_001702\|5535\|4279-4307 | 29 | imported |
| 289 | Gene.BAK1\|NM_001188\|2203\|1407-1369 | 39 | imported |
| 290 | Gene.BAK1\|NM_001188\|2203\|1369-1407 | 39 | imported |
| 291 | Gene.BAK1\|NM_001188\|2203\|1436-1405 | 32 | imported |
| 292 | Gene.BAK1\|NM_001188\|2203\|1405-1436 | 32 | imported |
| 293 | Gene.BAK1\|NM_001188\|2203\|1651-1621 | 31 | imported |
| 294 | Gene.BAK1\|NM_001188\|2203\|1621-1651 | 31 | imported |
| 295 | Gene.BAX\|NM_004324\|891\|328-301 | 28 | imported |
| 296 | Gene.BAX\|NM_004324\|891\|301-328 | 28 | imported |
| 297 | Gene.BAX\|NM_004324\|891\|396-371 | 26 | imported |
| 298 | Gene.BAX\|NM_004324\|891\|371-396 | 26 | imported |
| 299 | Gene.BAX\|NM_004324\|891\|432-401 | 32 | imported |
| 300 | Gene.BAX\|NM_004324\|891\|401-432 | 32 | imported |
| 301 | Gene.BCL2\|NM_000633\|6492\|997-973 | 25 | imported |
| 302 | Gene.BCL2\|NM_000633\|6492\|973-997 | 25 | imported |
| 303 | Gene.BCL2\|NM_000657\|1207\|865-841 | 25 | imported |
| 304 | Gene.BCL2\|NM_000657\|1207\|841-865 | 25 | imported |
| 305 | Gene.BCL2\|NM_000657\|1207\|961-931 | 31 | imported |
| 306 | Gene.BCL2\|NM_000657\|1207\|931-961 | 31 | imported |
| 307 | Gene.BCL2L1\|NM_001191\|2627\|450-419 | 32 | imported |
| 308 | Gene.BCL2L1\|NM_001191\|2627\|419-450 | 32 | imported |
| 309 | Gene.BCL2L1\|NM_001191\|2627\|584-551 | 34 | imported |
| 310 | Gene.BCL2L1\|NM_001191\|2627\|551-584 | 34 | imported |
| 311 | Gene.BCL2L1\|NM_001191\|2627\|1436-1409 | 28 | imported |
| 312 | Gene.BCL2L1\|NM_001191\|2627\|1409-1436 | 28 | imported |
| 313 | Gene.BECN1\|NM_001313998\|2145\|1107-1081 | 27 | imported |
| 314 | Gene.BECN1\|NM_001313998\|2145\|1081-1107 | 27 | imported |
| 315 | Gene.BECN1\|NM_001313998\|2145\|1193-1153 | 41 | imported |
| 316 | Gene.BECN1\|NM_001313998\|2145\|1153-1193 | 41 | imported |
| 317 | Gene.BECN1\|NM_001314000\|1774\|769-736 | 34 | imported |
| 318 | Gene.BECN1\|NM_001314000\|1774\|736-769 | 34 | imported |
| 319 | Gene.BID\|NM_001196\|2217\|437-400 | 38 | imported |
| 320 | Gene.BID\|NM_001196\|2217\|400-437 | 38 | imported |
| 321 | Gene.BID\|NM_001196\|2217\|483-457 | 27 | imported |
| 322 | Gene.BID\|NM_001196\|2217\|457-483 | 27 | imported |
| 323 | Gene.BID\|NM_001196\|2217\|740-704 | 37 | imported |
| 324 | Gene.BID\|NM_001196\|2217\|704-740 | 37 | imported |
| 325 | Gene.BIRC5\|NM_001012270\|2537\|279-253 | 27 | imported |
| 326 | Gene.BIRC5\|NM_001012270\|2537\|253-279 | 27 | imported |
| 327 | Gene.BIRC5\|NM_001012270\|2537\|1889-1849 | 41 | imported |
| 328 | Gene.BIRC5\|NM_001012270\|2537\|1849-1889 | 41 | imported |
| 329 | Gene.BIRC5\|NM_001168\|2655\|1807-1783 | 25 | imported |
| 330 | Gene.BIRC5\|NM_001168\|2655\|1783-1807 | 25 | imported |
| 331 | Gene.BLM\|NM_000057\|4555\|460-419 | 42 | imported |
| 332 | Gene.BLM\|NM_000057\|4555\|419-460 | 42 | imported |
| 333 | Gene.BLM\|NM_000057\|4555\|2319-2281 | 39 | imported |
| 334 | Gene.BLM\|NM_000057\|4555\|2281-2319 | 39 | imported |
| 335 | Gene.BLM\|NM_000057\|4555\|2657-2623 | 35 | imported |
| 336 | Gene.BLM\|NM_000057\|4555\|2623-2657 | 35 | imported |
| 337 | Gene.BMI1\|NM_005180\|3435\|560-523 | 38 | imported |
| 338 | Gene.BMI1\|NM_005180\|3435\|523-560 | 38 | imported |
| 339 | Gene.BMI1\|NM_005180\|3435\|711-668 | 44 | imported |
| 340 | Gene.BMI1\|NM_005180\|3435\|668-711 | 44 | imported |
| 341 | Gene.BMI1\|NM_005180\|3435\|2329-2292 | 38 | imported |
| 342 | Gene.BMI1\|NM_005180\|3435\|2292-2329 | 38 | imported |
| 343 | Gene.BMP1\|NM_001199\|2747\|831-806 | 26 | imported |
| 344 | Gene.BMP1\|NM_001199\|2747\|806-831 | 26 | imported |
| 345 | Gene.BMP1\|NM_001199\|2747\|1548-1519 | 30 | imported |
| 346 | Gene.BMP1\|NM_001199\|2747\|1519-1548 | 30 | imported |
| 347 | Gene.BMP1\|NM_001199\|2747\|2287-2255 | 33 | imported |
| 348 | Gene.BMP1\|NM_001199\|2747\|2255-2287 | 33 | imported |
| 349 | Gene.BMP2\|NM_001200\|3191\|1354-1324 | 31 | imported |
| 350 | Gene.BMP2\|NM_001200\|3191\|1324-1354 | 31 | imported |
| 351 | Gene.BMP2\|NM_001200\|3191\|1680-1648 | 33 | imported |
| 352 | Gene.BMP2\|NM_001200\|3191\|1648-1680 | 33 | imported |
| 353 | Gene.BMP2\|NM_001200\|3191\|2812-2782 | 31 | imported |
| 354 | Gene.BMP2\|NM_001200\|3191\|2782-2812 | 31 | imported |
| 355 | Gene.BMP7\|NM_001719\|4049\|1045-1021 | 25 | imported |
| 356 | Gene.BMP7\|NM_001719\|4049\|1021-1045 | 25 | imported |
| 357 | Gene.BMP7\|NM_001719\|4049\|1218-1191 | 28 | imported |
| 358 | Gene.BMP7\|NM_001719\|4049\|1191-1218 | 28 | imported |
| 359 | Gene.BMP7\|NM_001719\|4049\|1250-1225 | 26 | imported |
| 360 | Gene.BMP7\|NM_001719\|4049\|1225-1250 | 26 | imported |
| 361 | Gene.BNIP3\|NM_004052\|1661\|837-799 | 39 | imported |
| 362 | Gene.BNIP3\|NM_004052\|1661\|799-837 | 39 | imported |
| 363 | Gene.BNIP3\|NM_004052\|1661\|876-841 | 36 | imported |
| 364 | Gene.BNIP3\|NM_004052\|1661\|841-876 | 36 | imported |
| 365 | Gene.BNIP3\|NM_004052\|1661\|1454-1415 | 40 | imported |
| 366 | Gene.BNIP3\|NM_004052\|1661\|1415-1454 | 40 | imported |
| 367 | Gene.BPI\|NM_001725\|1901\|609-577 | 33 | imported |
| 368 | Gene.BPI\|NM_001725\|1901\|577-609 | 33 | imported |
| 369 | Gene.BPI\|NM_001725\|1901\|672-641 | 32 | imported |
| 370 | Gene.BPI\|NM_001725\|1901\|641-672 | 32 | imported |
| 371 | Gene.BPI\|NM_001725\|1901\|909-881 | 29 | imported |
| 372 | Gene.BPI\|NM_001725\|1901\|881-909 | 29 | imported |
| 373 | Gene.BRCA1\|NM_007297\|7132\|705-661 | 45 | imported |
| 374 | Gene.BRCA1\|NM_007297\|7132\|661-705 | 45 | imported |
| 375 | Gene.BRCA1\|NM_007298\|3699\|1212-1179 | 34 | imported |
| 376 | Gene.BRCA1\|NM_007298\|3699\|1179-1212 | 34 | imported |
| 377 | Gene.BRCA1\|NM_007298\|3699\|2200-2171 | 30 | imported |
| 378 | Gene.BRCA1\|NM_007298\|3699\|2171-2200 | 30 | imported |
| 379 | Gene.BRCA2\|NM_000059\|11386\|1463-1426 | 38 | imported |
| 380 | Gene.BRCA2\|NM_000059\|11386\|1426-1463 | 38 | imported |
| 381 | Gene.BRCA2\|NM_000059\|11386\|6410-6366 | 45 | imported |
| 382 | Gene.BRCA2\|NM_000059\|11386\|6366-6410 | 45 | imported |
| 383 | Gene.BRCA2\|NM_000059\|11386\|11159-11116 | 44 | imported |
| 384 | Gene.BRCA2\|NM_000059\|11386\|11116-11159 | 44 | imported |
| 385 | Gene.CAMK2N1\|NM_018584\|2371\|1094-1061 | 34 | imported |
| 386 | Gene.CAMK2N1\|NM_018584\|2371\|1061-1094 | 34 | imported |
| 387 | Gene.CAMK2N1\|NM_018584\|2371\|1117-1081 | 37 | imported |
| 388 | Gene.CAMK2N1\|NM_018584\|2371\|1081-1117 | 37 | imported |
| 389 | Gene.CAMK2N1\|NM_018584\|2371\|1950-1921 | 30 | imported |
| 390 | Gene.CAMK2N1\|NM_018584\|2371\|1921-1950 | 30 | imported |
| 391 | Gene.CARMA3\|NM_014550\|3912\|1083-1057 | 27 | imported |
| 392 | Gene.CARMA3\|NM_014550\|3912\|1057-1083 | 27 | imported |
| 393 | Gene.CARMA3\|NM_014550\|3912\|2666-2641 | 26 | imported |
| 394 | Gene.CARMA3\|NM_014550\|3912\|2641-2666 | 26 | imported |
| 395 | Gene.CARMA3\|NM_014550\|3912\|3363-3334 | 30 | imported |
| 396 | Gene.CARMA3\|NM_014550\|3912\|3334-3363 | 30 | imported |
| 397 | Gene.CASP3\|NM_004346\|2689\|472-438 | 35 | imported |
| 398 | Gene.CASP3\|NM_004346\|2689\|438-472 | 35 | imported |
| 399 | Gene.CASP3\|NM_004346\|2689\|2080-2048 | 33 | imported |
| 400 | Gene.CASP3\|NM_004346\|2689\|2048-2080 | 33 | imported |
| 401 | Gene.CASP3\|NM_004346\|2689\|2376-2347 | 30 | imported |
| 402 | Gene.CASP3\|NM_004346\|2689\|2347-2376 | 30 | imported |
| 403 | Gene.CASP8\|NM_001080124\|2750\|332-300 | 33 | imported |
| 404 | Gene.CASP8\|NM_001080124\|2750\|300-332 | 33 | imported |
| 405 | Gene.CASP8\|NM_033356\|2655\|254-221 | 34 | imported |
| 406 | Gene.CASP8\|NM_033356\|2655\|221-254 | 34 | imported |
| 407 | Gene.CASP8\|NM_033356\|2655\|448-419 | 30 | imported |
| 408 | Gene.CASP8\|NM_033356\|2655\|419-448 | 30 | imported |
| 409 | Gene.CAV1\|NM_001172895\|3212\|1061-1027 | 35 | imported |
| 410 | Gene.CAV1\|NM_001172895\|3212\|1027-1061 | 35 | imported |
| 411 | Gene.CAV1\|NM_001172895\|3212\|1852-1810 | 43 | imported |
| 412 | Gene.CAV1\|NM_001172895\|3212\|1810-1852 | 43 | imported |
| 413 | Gene.CAV1\|NM_001172896\|2647\|356-331 | 26 | imported |
| 414 | Gene.CAV1\|NM_001172896\|2647\|331-356 | 26 | imported |
| 415 | Gene.CAV2\|NM_001206747\|3121\|584-547 | 38 | imported |
| 416 | Gene.CAV2\|NM_001206747\|3121\|547-584 | 38 | imported |
| 417 | Gene.CAV2\|NM_001206747\|3121\|683-651 | 33 | imported |
| 418 | Gene.CAV2\|NM_001206747\|3121\|651-683 | 33 | imported |
| 419 | Gene.CAV2\|NM_001206747\|3121\|2603-2575 | 29 | imported |
| 420 | Gene.CAV2\|NM_001206747\|3121\|2575-2603 | 29 | imported |
| 421 | Gene.CCL2\|NM_002982\|760\|89-61 | 29 | imported |
| 422 | Gene.CCL2\|NM_002982\|760\|61-89 | 29 | imported |
| 423 | Gene.CCL2\|NM_002982\|760\|130-101 | 30 | imported |
| 424 | Gene.CCL2\|NM_002982\|760\|101-130 | 30 | imported |
| 425 | Gene.CCL2\|NM_002982\|760\|147-121 | 27 | imported |
| 426 | Gene.CCL2\|NM_002982\|760\|121-147 | 27 | imported |
| 427 | Gene.CCL5\|NM_001278736\|1319\|233-199 | 35 | imported |
| 428 | Gene.CCL5\|NM_001278736\|1319\|199-233 | 35 | imported |
| 429 | Gene.CCL5\|NM_001278736\|1319\|468-430 | 39 | imported |
| 430 | Gene.CCL5\|NM_001278736\|1319\|430-468 | 39 | imported |
| 431 | Gene.CCL5\|NM_001278736\|1319\|559-529 | 31 | imported |
| 432 | Gene.CCL5\|NM_001278736\|1319\|529-559 | 31 | imported |
| 433 | Gene.CD24\|NM_013230\|2513\|281-253 | 29 | imported |
| 434 | Gene.CD24\|NM_013230\|2513\|253-281 | 29 | imported |
| 435 | Gene.CD24\|NM_013230\|2513\|1356-1324 | 33 | imported |
| 436 | Gene.CD24\|NM_013230\|2513\|1324-1356 | 33 | imported |
| 437 | Gene.CD24\|NM_013230\|2513\|1608-1576 | 33 | imported |
| 438 | Gene.CD24\|NM_013230\|2513\|1576-1608 | 33 | imported |
| 439 | Gene.CD34\|NM_001025109\|2621\|694-661 | 34 | imported |
| 440 | Gene.CD34\|NM_001025109\|2621\|661-694 | 34 | imported |
| 441 | Gene.CD34\|NM_001025109\|2621\|827-793 | 35 | imported |
| 442 | Gene.CD34\|NM_001025109\|2621\|793-827 | 35 | imported |
| 443 | Gene.CD34\|NM_001773\|2816\|1591-1561 | 31 | imported |
| 444 | Gene.CD34\|NM_001773\|2816\|1561-1591 | 31 | imported |
| 445 | Gene.CD38\|NM_001775\|5694\|1138-1105 | 34 | imported |
| 446 | Gene.CD38\|NM_001775\|5694\|1105-1138 | 34 | imported |
| 447 | Gene.CD38\|NM_001775\|5694\|1185-1153 | 33 | imported |
| 448 | Gene.CD38\|NM_001775\|5694\|1153-1185 | 33 | imported |
| 449 | Gene.CD38\|NM_001775\|5694\|853-817 | 37 | imported |
| 450 | Gene.CD38\|NM_001775\|5694\|817-853 | 37 | imported |
| 451 | Gene.CD44\|NM_000610\|5748\|3683-3649 | 35 | imported |
| 452 | Gene.CD44\|NM_000610\|5748\|3649-3683 | 35 | imported |
| 453 | Gene.CD44\|NM_000610\|5748\|4117-4081 | 37 | imported |
| 454 | Gene.CD44\|NM_000610\|5748\|4081-4117 | 37 | imported |
| 455 | Gene.CD44\|NM_001001390\|5001\|2891-2857 | 35 | imported |
| 456 | Gene.CD44\|NM_001001390\|5001\|2857-2891 | 35 | imported |
| 457 | Gene.CD69\|NM_001781\|1676\|145-113 | 33 | imported |
| 458 | Gene.CD69\|NM_001781\|1676\|113-145 | 33 | imported |
| 459 | Gene.CD69\|NM_001781\|1676\|536-505 | 32 | imported |
| 460 | Gene.CD69\|NM_001781\|1676\|505-536 | 32 | imported |
| 461 | Gene.CD69\|NM_001781\|1676\|745-701 | 45 | imported |
| 462 | Gene.CD69\|NM_001781\|1676\|701-745 | 45 | imported |
| 463 | Gene.CDC42\|NM_001039802\|2308\|665-628 | 38 | imported |
| 464 | Gene.CDC42\|NM_001039802\|2308\|628-665 | 38 | imported |
| 465 | Gene.CDC42\|NM_001039802\|2308\|777-742 | 36 | imported |
| 466 | Gene.CDC42\|NM_001039802\|2308\|742-777 | 36 | imported |
| 467 | Gene.CDC42\|NM_001791\|2182\|331-289 | 43 | imported |
| 468 | Gene.CDC42\|NM_001791\|2182\|289-331 | 43 | imported |
| 469 | Gene.CDH1\|NM_004360\|4845\|27-1 | 27 | imported |
| 470 | Gene.CDH1\|NM_004360\|4845\|1-27 | 27 | imported |
| 471 | Gene.CDH1\|NM_004360\|4845\|1153-1121 | 33 | imported |
| 472 | Gene.CDH1\|NM_004360\|4845\|1121-1153 | 33 | imported |
| 473 | Gene.CDH1\|NM_004360\|4845\|2030-2001 | 30 | imported |
| 474 | Gene.CDH1\|NM_004360\|4845\|2001-2030 | 30 | imported |
| 475 | Gene.CDH2\|NM_001792\|4339\|579-541 | 39 | imported |
| 476 | Gene.CDH2\|NM_001792\|4339\|541-579 | 39 | imported |
| 477 | Gene.CDH2\|NM_001792\|4339\|1013-973 | 41 | imported |
| 478 | Gene.CDH2\|NM_001792\|4339\|973-1013 | 41 | imported |
| 479 | Gene.CDH2\|NM_001792\|4339\|4321-4285 | 37 | imported |
| 480 | Gene.CDH2\|NM_001792\|4339\|4285-4321 | 37 | imported |
| 481 | Gene.CDH5\|NM_001795\|4149\|834-806 | 29 | imported |
| 482 | Gene.CDH5\|NM_001795\|4149\|806-834 | 29 | imported |
| 483 | Gene.CDH5\|NM_001795\|4149\|1507-1471 | 37 | imported |
| 484 | Gene.CDH5\|NM_001795\|4149\|1471-1507 | 37 | imported |
| 485 | Gene.CDH5\|NM_001795\|4149\|3457-3431 | 27 | imported |
| 486 | Gene.CDH5\|NM_001795\|4149\|3431-3457 | 27 | imported |
| 487 | Gene.CDKN1B\|NM_004064\|2535\|770-736 | 35 | imported |
| 488 | Gene.CDKN1B\|NM_004064\|2535\|736-770 | 35 | imported |
| 489 | Gene.CDKN1B\|NM_004064\|2535\|794-757 | 38 | imported |
| 490 | Gene.CDKN1B\|NM_004064\|2535\|757-794 | 38 | imported |
| 491 | Gene.CDKN1B\|NM_004064\|2535\|1013-988 | 26 | imported |
| 492 | Gene.CDKN1B\|NM_004064\|2535\|988-1013 | 26 | imported |
| 493 | Gene.CDKN2A\|NM_058195\|1164\|455-431 | 25 | imported |
| 494 | Gene.CDKN2A\|NM_058195\|1164\|431-455 | 25 | imported |
| 495 | Gene.CDKN2A\|NM_000077\|1267\|779-749 | 31 | imported |
| 496 | Gene.CDKN2A\|NM_000077\|1267\|749-779 | 31 | imported |
| 497 | Gene.CDKN2A\|NM_000077\|1267\|839-804 | 36 | imported |
| 498 | Gene.CDKN2A\|NM_000077\|1267\|804-839 | 36 | imported |
| 499 | Gene.CFLAR\|NM_001127184\|1299\|832-793 | 40 | imported |
| 500 | Gene.CFLAR\|NM_001127184\|1299\|793-832 | 40 | imported |
| 501 | Gene.CFLAR\|NM_001127184\|1299\|905-870 | 36 | imported |
| 502 | Gene.CFLAR\|NM_001127184\|1299\|870-905 | 36 | imported |
| 503 | Gene.CFLAR\|NM_001127184\|1299\|931-892 | 40 | imported |
| 504 | Gene.CFLAR\|NM_001127184\|1299\|892-931 | 40 | imported |
| 505 | Gene.CLN3\|NM_000086\|1879\|1037-1009 | 29 | imported |
| 506 | Gene.CLN3\|NM_000086\|1879\|1009-1037 | 29 | imported |
| 507 | Gene.CLN3\|NM_000086\|1879\|1693-1665 | 29 | imported |
| 508 | Gene.CLN3\|NM_000086\|1879\|1665-1693 | 29 | imported |
| 509 | Gene.CLN3\|NM_001042432\|1915\|1055-1025 | 31 | imported |
| 510 | Gene.CLN3\|NM_001042432\|1915\|1025-1055 | 31 | imported |
| 511 | Gene.CMKLR1\|NM_001142343\|5496\|1131-1105 | 27 | imported |
| 512 | Gene.CMKLR1\|NM_001142343\|5496\|1105-1131 | 27 | imported |
| 513 | Gene.CMKLR1\|NM_001142343\|5496\|4037-4003 | 35 | imported |
| 514 | Gene.CMKLR1\|NM_001142343\|5496\|4003-4037 | 35 | imported |
| 515 | Gene.CMKLR1\|NM_001142344\|5283\|1040-1013 | 28 | imported |
| 516 | Gene.CMKLR1\|NM_001142344\|5283\|1013-1040 | 28 | imported |
| 517 | Gene.COL18A1\|NM_030582\|5910\|2918-2892 | 27 | imported |
| 518 | Gene.COL18A1\|NM_030582\|5910\|2892-2918 | 27 | imported |
| 519 | Gene.COL18A1\|NM_030582\|5910\|3162-3137 | 26 | imported |
| 520 | Gene.COL18A1\|NM_030582\|5910\|3137-3162 | 26 | imported |
| 521 | Gene.COL18A1\|NM_130445\|5443\|476-451 | 26 | imported |
| 522 | Gene.COL18A1\|NM_130445\|5443\|451-476 | 26 | imported |
| 523 | Gene.COL4A3\|NM_000091\|8114\|1595-1565 | 31 | imported |
| 524 | Gene.COL4A3\|NM_000091\|8114\|1565-1595 | 31 | imported |
| 525 | Gene.COL4A3\|NM_000091\|8114\|2409-2381 | 29 | imported |
| 526 | Gene.COL4A3\|NM_000091\|8114\|2381-2409 | 29 | imported |
| 527 | Gene.COL4A3\|NM_000091\|8114\|7316-7277 | 40 | imported |
| 528 | Gene.COL4A3\|NM_000091\|8114\|7277-7316 | 40 | imported |
| 529 | Gene.CSF2\|NM_000758\|800\|181-151 | 31 | imported |
| 530 | Gene.CSF2\|NM_000758\|800\|151-181 | 31 | imported |
| 531 | Gene.CSF2\|NM_000758\|800\|238-201 | 38 | imported |
| 532 | Gene.CSF2\|NM_000758\|800\|201-238 | 38 | imported |
| 533 | Gene.CSF2\|NM_000758\|800\|550-511 | 40 | imported |
| 534 | Gene.CSF2\|NM_000758\|800\|511-550 | 40 | imported |
| 535 | Gene.CSF2RA\|NM_001161529\|1983\|371-341 | 31 | imported |
| 536 | Gene.CSF2RA\|NM_001161529\|1983\|341-371 | 31 | imported |
| 537 | Gene.CSF2RA\|NM_001161529\|1983\|585-545 | 41 | imported |
| 538 | Gene.CSF2RA\|NM_001161529\|1983\|545-585 | 41 | imported |
| 539 | Gene.CSF2RA\|NM_001161529\|1983\|738-698 | 41 | imported |
| 540 | Gene.CSF2RA\|NM_001161529\|1983\|698-738 | 41 | imported |
| 541 | Gene.CTGF\|NM_001901\|2358\|529-501 | 29 | imported |
| 542 | Gene.CTGF\|NM_001901\|2358\|501-529 | 29 | imported |
| 543 | Gene.CTGF\|NM_001901\|2358\|865-841 | 25 | imported |
| 544 | Gene.CTGF\|NM_001901\|2358\|841-865 | 25 | imported |
| 545 | Gene.CTGF\|NM_001901\|2358\|1055-1021 | 35 | imported |
| 546 | Gene.CTGF\|NM_001901\|2358\|1021-1055 | 35 | imported |
| 547 | Gene.CTNNB1\|NM_001098209\|3415\|320-291 | 30 | imported |
| 548 | Gene.CTNNB1\|NM_001098209\|3415\|291-320 | 30 | imported |
| 549 | Gene.CTNNB1\|NM_001098209\|3415\|2267-2234 | 34 | imported |
| 550 | Gene.CTNNB1\|NM_001098209\|3415\|2234-2267 | 34 | imported |
| 551 | Gene.CTNNB1\|NM_001098209\|3415\|2352-2321 | 32 | imported |
| 552 | Gene.CTNNB1\|NM_001098209\|3415\|2321-2352 | 32 | imported |
| 553 | Gene.CTSB\|NM_001317237\|3773\|577-545 | 33 | imported |
| 554 | Gene.CTSB\|NM_001317237\|3773\|545-577 | 33 | imported |
| 555 | Gene.CTSB\|NM_147780\|3966\|1886-1849 | 38 | imported |
| 556 | Gene.CTSB\|NM_147780\|3966\|1849-1886 | 38 | imported |
| 557 | Gene.CTSB\|NM_147781\|3923\|396-364 | 33 | imported |
| 558 | Gene.CTSB\|NM_147781\|3923\|364-396 | 33 | imported |
| 559 | Gene.CTSS\|NM_004079\|4107\|852-817 | 36 | imported |
| 560 | Gene.CTSS\|NM_004079\|4107\|817-852 | 36 | imported |
| 561 | Gene.CTSS\|NM_004079\|4107\|1019-987 | 33 | imported |
| 562 | Gene.CTSS\|NM_004079\|4107\|987-1019 | 33 | imported |
| 563 | Gene.CTSS\|NM_004079\|4107\|3309-3265 | 45 | imported |
| 564 | Gene.CTSS\|NM_004079\|4107\|3265-3309 | 45 | imported |
| 565 | Gene.CX3CL1\|NM_002996\|3338\|1295-1261 | 35 | imported |
| 566 | Gene.CX3CL1\|NM_002996\|3338\|1261-1295 | 35 | imported |
| 567 | Gene.CX3CL1\|NM_002996\|3338\|2216-2185 | 32 | imported |
| 568 | Gene.CX3CL1\|NM_002996\|3338\|2185-2216 | 32 | imported |
| 569 | Gene.CX3CL1\|NM_002996\|3338\|2414-2381 | 34 | imported |
| 570 | Gene.CX3CL1\|NM_002996\|3338\|2381-2414 | 34 | imported |
| 571 | Gene.CXCL1\|NM_001511\|1184\|145-121 | 25 | imported |
| 572 | Gene.CXCL1\|NM_001511\|1184\|121-145 | 25 | imported |
| 573 | Gene.CXCL1\|NM_001511\|1184\|349-321 | 29 | imported |
| 574 | Gene.CXCL1\|NM_001511\|1184\|321-349 | 29 | imported |
| 575 | Gene.CXCL1\|NM_001511\|1184\|415-381 | 35 | imported |
| 576 | Gene.CXCL1\|NM_001511\|1184\|381-415 | 35 | imported |
| 577 | Gene.CXCL10\|NM_001565\|1227\|38-1 | 38 | imported |
| 578 | Gene.CXCL10\|NM_001565\|1227\|1-38 | 38 | imported |
| 579 | Gene.CXCL10\|NM_001565\|1227\|56-21 | 36 | imported |
| 580 | Gene.CXCL10\|NM_001565\|1227\|21-56 | 36 | imported |
| 581 | Gene.CXCL10\|NM_001565\|1227\|81-41 | 41 | imported |
| 582 | Gene.CXCL10\|NM_001565\|1227\|41-81 | 41 | imported |
| 583 | Gene.CXCR4\|NM_001008540\|1912\|588-561 | 28 | imported |
| 584 | Gene.CXCR4\|NM_001008540\|1912\|561-588 | 28 | imported |
| 585 | Gene.CXCR4\|NM_001008540\|1912\|782-753 | 30 | imported |
| 586 | Gene.CXCR4\|NM_001008540\|1912\|753-782 | 30 | imported |
| 587 | Gene.CXCR4\|NM_001008540\|1912\|1292-1265 | 28 | imported |
| 588 | Gene.CXCR4\|NM_001008540\|1912\|1265-1292 | 28 | imported |
| 589 | Gene.DCK\|NM_000788\|2618\|362-331 | 32 | imported |
| 590 | Gene.DCK\|NM_000788\|2618\|331-362 | 32 | imported |
| 591 | Gene.DCK\|NM_000788\|2618\|833-793 | 41 | imported |
| 592 | Gene.DCK\|NM_000788\|2618\|793-833 | 41 | imported |
| 593 | Gene.DCK\|NM_000788\|2618\|1695-1651 | 45 | imported |
| 594 | Gene.DCK\|NM_000788\|2618\|1651-1695 | 45 | imported |
| 595 | Gene.DCLRE1C\|NM_001033855\|6298\|2106-2068 | 39 | imported |
| 596 | Gene.DCLRE1C\|NM_001033855\|6298\|2068-2106 | 39 | imported |
| 597 | Gene.DCLRE1C\|NM_001033855\|6298\|2427-2386 | 42 | imported |
| 598 | Gene.DCLRE1C\|NM_001033855\|6298\|2386-2427 | 42 | imported |
| 599 | Gene.DCLRE1C\|NM_001033855\|6298\|3534-3499 | 36 | imported |
| 600 | Gene.DCLRE1C\|NM_001033855\|6298\|3499-3534 | 36 | imported |
| 601 | Gene.DCTN4\|NM_001135643\|3958\|1519-1486 | 34 | imported |
| 602 | Gene.DCTN4\|NM_001135643\|3958\|1486-1519 | 34 | imported |
| 603 | Gene.DCTN4\|NM_001135643\|3958\|1746-1717 | 30 | imported |
| 604 | Gene.DCTN4\|NM_001135643\|3958\|1717-1746 | 30 | imported |
| 605 | Gene.DCTN4\|NM_001135644\|4105\|1832-1803 | 30 | imported |
| 606 | Gene.DCTN4\|NM_001135644\|4105\|1803-1832 | 30 | imported |
| 607 | Gene.DDIT3\|NM_001195053\|1081\|913-881 | 33 | imported |
| 608 | Gene.DDIT3\|NM_001195053\|1081\|881-913 | 33 | imported |
| 609 | Gene.DDIT3\|NM_001195053\|1081\|944-911 | 34 | imported |
| 610 | Gene.DDIT3\|NM_001195053\|1081\|911-944 | 34 | imported |
| 611 | Gene.DDIT3\|NM_001195053\|1081\|963-931 | 33 | imported |
| 612 | Gene.DDIT3\|NM_001195053\|1081\|931-963 | 33 | imported |
| 613 | Gene.DEFA3\|NM_005217\|501\|179-151 | 29 | imported |
| 614 | Gene.DEFA3\|NM_005217\|501\|151-179 | 29 | imported |
| 615 | Gene.DEFA3\|NM_005217\|501\|206-181 | 26 | imported |
| 616 | Gene.DEFA3\|NM_005217\|501\|181-206 | 26 | imported |
| 617 | Gene.DEFA3\|NM_005217\|501\|222-191 | 32 | imported |
| 618 | Gene.DEFA3\|NM_005217\|501\|191-222 | 32 | imported |
| 619 | Gene.DEFA3\|NM_005217\|501\|260-231 | 30 | imported |
| 620 | Gene.DEFA3\|NM_005217\|501\|231-260 | 30 | imported |
| 621 | Gene.DEFA3\|NM_005217\|501\|343-311 | 33 | imported |
| 622 | Gene.DEFA3\|NM_005217\|501\|311-343 | 33 | imported |
| 623 | Gene.DMC1\|NM_001278208\|2082\|619-579 | 41 | imported |
| 624 | Gene.DMC1\|NM_001278208\|2082\|579-619 | 41 | imported |
| 625 | Gene.DMC1\|NM_001278208\|2082\|969-936 | 34 | imported |
| 626 | Gene.DMC1\|NM_001278208\|2082\|936-969 | 34 | imported |
| 627 | Gene.DMC1\|NM_007068\|2281\|292-248 | 45 | imported |
| 628 | Gene.DMC1\|NM_007068\|2281\|248-292 | 45 | imported |
| 629 | Gene.DNMT1\|NM_001130823\|5470\|770-737 | 34 | imported |
| 630 | Gene.DNMT1\|NM_001130823\|5470\|737-770 | 34 | imported |
| 631 | Gene.DNMT1\|NM_001130823\|5470\|3891-3865 | 27 | imported |
| 632 | Gene.DNMT1\|NM_001130823\|5470\|3865-3891 | 27 | imported |
| 633 | Gene.DNMT1\|NM_001379\|5422\|3090-3061 | 30 | imported |
| 634 | Gene.DNMT1\|NM_001379\|5422\|3061-3090 | 30 | imported |
| 635 | Gene.DRAM1\|NM_018370\|3553\|1153-1111 | 43 | imported |
| 636 | Gene.DRAM1\|NM_018370\|3553\|1111-1153 | 43 | imported |
| 637 | Gene.DRAM1\|NM_018370\|3553\|1237-1201 | 37 | imported |
| 638 | Gene.DRAM1\|NM_018370\|3553\|1201-1237 | 37 | imported |
| 639 | Gene.DRAM1\|NM_018370\|3553\|3090-3061 | 30 | imported |
| 640 | Gene.DRAM1\|NM_018370\|3553\|3061-3090 | 30 | imported |
| 641 | Gene.DRAM2\|NM_178454\|1901\|741-705 | 37 | imported |
| 642 | Gene.DRAM2\|NM_178454\|1901\|705-741 | 37 | imported |
| 643 | Gene.DRAM2\|NM_178454\|1901\|899-865 | 35 | imported |
| 644 | Gene.DRAM2\|NM_178454\|1901\|865-899 | 35 | imported |
| 645 | Gene.DRAM2\|NM_178454\|1901\|1031-993 | 39 | imported |
| 646 | Gene.DRAM2\|NM_178454\|1901\|993-1031 | 39 | imported |
| 647 | Gene.DSC2\|NM_004949\|5260\|699-661 | 39 | imported |
| 648 | Gene.DSC2\|NM_004949\|5260\|661-699 | 39 | imported |
| 649 | Gene.DSC2\|NM_004949\|5260\|1539-1497 | 43 | imported |
| 650 | Gene.DSC2\|NM_004949\|5260\|1497-1539 | 43 | imported |
| 651 | Gene.DSC2\|NM_004949\|5260\|2374-2333 | 42 | imported |
| 652 | Gene.DSC2\|NM_004949\|5260\|2333-2374 | 42 | imported |
| 653 | Gene.EDN1\|NM_001168319\|2109\|209-181 | 29 | imported |
| 654 | Gene.EDN1\|NM_001168319\|2109\|181-209 | 29 | imported |
| 655 | Gene.EDN1\|NM_001168319\|2109\|880-847 | 34 | imported |
| 656 | Gene.EDN1\|NM_001168319\|2109\|847-880 | 34 | imported |
| 657 | Gene.EDN1\|NM_001168319\|2109\|1572-1531 | 42 | imported |
| 658 | Gene.EDN1\|NM_001168319\|2109\|1531-1572 | 42 | imported |
| 659 | Gene.EGF\|NM_001178130\|5577\|2720-2680 | 41 | imported |
| 660 | Gene.EGF\|NM_001178130\|5577\|2680-2720 | 41 | imported |
| 661 | Gene.EGF\|NM_001178131\|5574\|1585-1552 | 34 | imported |
| 662 | Gene.EGF\|NM_001178131\|5574\|1552-1585 | 34 | imported |
| 663 | Gene.EGF\|NM_001178131\|5574\|3229-3197 | 33 | imported |
| 664 | Gene.EGF\|NM_001178131\|5574\|3197-3229 | 33 | imported |
| 665 | Gene.EGFR\|NM_005228\|6369\|561-531 | 31 | imported |
| 666 | Gene.EGFR\|NM_005228\|6369\|531-561 | 31 | imported |
| 667 | Gene.EGFR\|NM_005228\|6369\|1413-1379 | 35 | imported |
| 668 | Gene.EGFR\|NM_005228\|6369\|1379-1413 | 35 | imported |
| 669 | Gene.EGFR\|NM_201282\|2239\|524-495 | 30 | imported |
| 670 | Gene.EGFR\|NM_201282\|2239\|495-524 | 30 | imported |
| 671 | Gene.EIF2A\|NM_032025\|4003\|360-331 | 30 | imported |
| 672 | Gene.EIF2A\|NM_032025\|4003\|331-360 | 30 | imported |
| 673 | Gene.EIF2A\|NM_032025\|4003\|758-727 | 32 | imported |
| 674 | Gene.EIF2A\|NM_032025\|4003\|727-758 | 32 | imported |
| 675 | Gene.EIF2A\|NM_032025\|4003\|3706-3664 | 43 | imported |
| 676 | Gene.EIF2A\|NM_032025\|4003\|3664-3706 | 43 | imported |
| 677 | Gene.EIF2AK3\|NM_001313915\|4233\|532-491 | 42 | imported |
| 678 | Gene.EIF2AK3\|NM_001313915\|4233\|491-532 | 42 | imported |
| 679 | Gene.EIF2AK3\|NM_001313915\|4233\|3150-3116 | 35 | imported |
| 680 | Gene.EIF2AK3\|NM_001313915\|4233\|3116-3150 | 35 | imported |
| 681 | Gene.EIF2AK3\|NM_004836\|4659\|1722-1678 | 45 | imported |
| 682 | Gene.EIF2AK3\|NM_004836\|4659\|1678-1722 | 45 | imported |
| 683 | Gene.EIF4G1\|NM_001194946\|5559\|2292-2255 | 38 | imported |
| 684 | Gene.EIF4G1\|NM_001194946\|5559\|2255-2292 | 38 | imported |
| 685 | Gene.EIF4G1\|NM_001291157\|5581\|3315-3291 | 25 | imported |
| 686 | Gene.EIF4G1\|NM_001291157\|5581\|3291-3315 | 25 | imported |
| 687 | Gene.EIF4G1\|NM_004953\|5050\|2757-2731 | 27 | imported |
| 688 | Gene.EIF4G1\|NM_004953\|5050\|2731-2757 | 27 | imported |
| 689 | Gene.EMC7\|NM_020154\|1075\|323-281 | 43 | imported |
| 690 | Gene.EMC7\|NM_020154\|1075\|281-323 | 43 | imported |
| 691 | Gene.EMC7\|NM_020154\|1075\|347-311 | 37 | imported |
| 692 | Gene.EMC7\|NM_020154\|1075\|311-347 | 37 | imported |
| 693 | Gene.EMC7\|NM_020154\|1075\|422-381 | 42 | imported |
| 694 | Gene.EMC7\|NM_020154\|1075\|381-422 | 42 | imported |
| 695 | Gene.EME1\|NM_001166131\|2371\|1195-1161 | 35 | imported |
| 696 | Gene.EME1\|NM_001166131\|2371\|1161-1195 | 35 | imported |
| 697 | Gene.EME1\|NM_001166131\|2371\|1352-1321 | 32 | imported |
| 698 | Gene.EME1\|NM_001166131\|2371\|1321-1352 | 32 | imported |
| 699 | Gene.EME1\|NM_001166131\|2371\|1750-1721 | 30 | imported |
| 700 | Gene.EME1\|NM_001166131\|2371\|1721-1750 | 30 | imported |
| 701 | Gene.EPAS1\|NM_001430\|5184\|117-87 | 31 | imported |
| 702 | Gene.EPAS1\|NM_001430\|5184\|87-117 | 31 | imported |
| 703 | Gene.EPAS1\|NM_001430\|5184\|1584-1549 | 36 | imported |
| 704 | Gene.EPAS1\|NM_001430\|5184\|1549-1584 | 36 | imported |
| 705 | Gene.EPAS1\|NM_001430\|5184\|1795-1764 | 32 | imported |
| 706 | Gene.EPAS1\|NM_001430\|5184\|1764-1795 | 32 | imported |
| 707 | Gene.EPHB4\|NM_004444\|4369\|2332-2295 | 38 | imported |
| 708 | Gene.EPHB4\|NM_004444\|4369\|2295-2332 | 38 | imported |
| 709 | Gene.EPHB4\|NM_004444\|4369\|2842-2813 | 30 | imported |
| 710 | Gene.EPHB4\|NM_004444\|4369\|2813-2842 | 30 | imported |
| 711 | Gene.EPHB4\|NM_004444\|4369\|3689-3664 | 26 | imported |
| 712 | Gene.EPHB4\|NM_004444\|4369\|3664-3689 | 26 | imported |
| 713 | Gene.ERBB2\|NM_001005862\|4889\|1056-1026 | 31 | imported |
| 714 | Gene.ERBB2\|NM_001005862\|4889\|1026-1056 | 31 | imported |
| 715 | Gene.ERBB2\|NM_001005862\|4889\|3595-3568 | 28 | imported |
| 716 | Gene.ERBB2\|NM_001005862\|4889\|3568-3595 | 28 | imported |
| 717 | Gene.ERBB2\|NM_004448\|4664\|2762-2731 | 32 | imported |
| 718 | Gene.ERBB2\|NM_004448\|4664\|2731-2762 | 32 | imported |
| 719 | Gene.ERBB3\|NM_001005915\|1050\|298-271 | 28 | imported |
| 720 | Gene.ERBB3\|NM_001005915\|1050\|271-298 | 28 | imported |
| 721 | Gene.ERBB3\|NM_001005915\|1050\|400-371 | 30 | imported |
| 722 | Gene.ERBB3\|NM_001005915\|1050\|371-400 | 30 | imported |
| 723 | Gene.ERBB3\|NM_001005915\|1050\|462-431 | 32 | imported |
| 724 | Gene.ERBB3\|NM_001005915\|1050\|431-462 | 32 | imported |
| 725 | Gene.ERCC1\|NM_202001\|1291\|204-177 | 28 | imported |
| 726 | Gene.ERCC1\|NM_202001\|1291\|177-204 | 28 | imported |
| 727 | Gene.ERCC1\|NM_202001\|1291\|633-606 | 28 | imported |
| 728 | Gene.ERCC1\|NM_202001\|1291\|606-633 | 28 | imported |
| 729 | Gene.ERCC1\|NM_202001\|1291\|785-760 | 26 | imported |
| 730 | Gene.ERCC1\|NM_202001\|1291\|760-785 | 26 | imported |
| 731 | Gene.EZR\|NM_001111077\|3138\|161-131 | 31 | imported |
| 732 | Gene.EZR\|NM_001111077\|3138\|131-161 | 31 | imported |
| 733 | Gene.EZR\|NM_001111077\|3138\|507-469 | 39 | imported |
| 734 | Gene.EZR\|NM_001111077\|3138\|469-507 | 39 | imported |
| 735 | Gene.EZR\|NM_003379\|3172\|1057-1027 | 31 | imported |
| 736 | Gene.EZR\|NM_003379\|3172\|1027-1057 | 31 | imported |
| 737 | Gene.FABP4\|NM_001442\|838\|161-121 | 41 | imported |
| 738 | Gene.FABP4\|NM_001442\|838\|121-161 | 41 | imported |
| 739 | Gene.FABP4\|NM_001442\|838\|29-1 | 29 | imported |
| 740 | Gene.FABP4\|NM_001442\|838\|1-29 | 29 | imported |
| 741 | Gene.FABP4\|NM_001442\|838\|120-81 | 40 | imported |
| 742 | Gene.FABP4\|NM_001442\|838\|81-120 | 40 | imported |
| 743 | Gene.FAP\|NM_004460\|2837\|1639-1609 | 31 | imported |
| 744 | Gene.FAP\|NM_004460\|2837\|1609-1639 | 31 | imported |
| 745 | Gene.FAP\|NM_004460\|2837\|1976-1945 | 32 | imported |
| 746 | Gene.FAP\|NM_004460\|2837\|1945-1976 | 32 | imported |
| 747 | Gene.FAP\|NM_004460\|2837\|2173-2137 | 37 | imported |
| 748 | Gene.FAP\|NM_004460\|2837\|2137-2173 | 37 | imported |
| 749 | Gene.FAS\|NM_000043\|3951\|530-496 | 35 | imported |
| 750 | Gene.FAS\|NM_000043\|3951\|496-530 | 35 | imported |
| 751 | Gene.FAS\|NM_001320619\|3868\|571-545 | 27 | imported |
| 752 | Gene.FAS\|NM_001320619\|3868\|545-571 | 27 | imported |
| 753 | Gene.FAS\|NM_001320619\|3868\|614-577 | 38 | imported |
| 754 | Gene.FAS\|NM_001320619\|3868\|577-614 | 38 | imported |
| 755 | Gene.FASN\|NM_004104\|8481\|240-214 | 27 | imported |
| 756 | Gene.FASN\|NM_004104\|8481\|214-240 | 27 | imported |
| 757 | Gene.FASN\|NM_004104\|8481\|522-498 | 25 | imported |
| 758 | Gene.FASN\|NM_004104\|8481\|498-522 | 25 | imported |
| 759 | Gene.FASN\|NM_004104\|8481\|5279-5255 | 25 | imported |
| 760 | Gene.FASN\|NM_004104\|8481\|5255-5279 | 25 | imported |
| 761 | Gene.FFAR2\|NM_005306\|2069\|365-341 | 25 | imported |
| 762 | Gene.FFAR2\|NM_005306\|2069\|341-365 | 25 | imported |
| 763 | Gene.FFAR2\|NM_005306\|2069\|811-783 | 29 | imported |
| 764 | Gene.FFAR2\|NM_005306\|2069\|783-811 | 29 | imported |
| 765 | Gene.FFAR2\|NM_005306\|2069\|1238-1208 | 31 | imported |
| 766 | Gene.FFAR2\|NM_005306\|2069\|1208-1238 | 31 | imported |
| 767 | Gene.FGF1\|NM_000800\|4162\|1016-981 | 36 | imported |
| 768 | Gene.FGF1\|NM_000800\|4162\|981-1016 | 36 | imported |
| 769 | Gene.FGF1\|NM_001144892\|3682\|340-311 | 30 | imported |
| 770 | Gene.FGF1\|NM_001144892\|3682\|311-340 | 30 | imported |
| 771 | Gene.FGF1\|NM_001144934\|3875\|1469-1441 | 29 | imported |
| 772 | Gene.FGF1\|NM_001144934\|3875\|1441-1469 | 29 | imported |
| 773 | Gene.FGF2\|NM_002006\|6774\|26-1 | 26 | imported |
| 774 | Gene.FGF2\|NM_002006\|6774\|1-26 | 26 | imported |
| 775 | Gene.FGF2\|NM_002006\|6774\|3513-3478 | 36 | imported |
| 776 | Gene.FGF2\|NM_002006\|6774\|3478-3513 | 36 | imported |
| 777 | Gene.FGF2\|NM_002006\|6774\|5798-5758 | 41 | imported |
| 778 | Gene.FGF2\|NM_002006\|6774\|5758-5798 | 41 | imported |
| 779 | Gene.FH\|NM_000143\|1877\|250-209 | 42 | imported |
| 780 | Gene.FH\|NM_000143\|1877\|209-250 | 42 | imported |
| 781 | Gene.FH\|NM_000143\|1877\|791-753 | 39 | imported |
| 782 | Gene.FH\|NM_000143\|1877\|753-791 | 39 | imported |
| 783 | Gene.FH\|NM_000143\|1877\|1247-1217 | 31 | imported |
| 784 | Gene.FH\|NM_000143\|1877\|1217-1247 | 31 | imported |
| 785 | Gene.FLT1\|NM_001160030\|2968\|1351-1326 | 26 | imported |
| 786 | Gene.FLT1\|NM_001160030\|2968\|1326-1351 | 26 | imported |
| 787 | Gene.FLT1\|NM_001160031\|1911\|444-417 | 28 | imported |
| 788 | Gene.FLT1\|NM_001160031\|1911\|417-444 | 28 | imported |
| 789 | Gene.FLT1\|NM_001160031\|1911\|1610-1585 | 26 | imported |
| 790 | Gene.FLT1\|NM_001160031\|1911\|1585-1610 | 26 | imported |
| 791 | Gene.FN1\|NM_054034\|2402\|636-601 | 36 | imported |
| 792 | Gene.FN1\|NM_054034\|2402\|601-636 | 36 | imported |
| 793 | Gene.FN1\|NM_054034\|2402\|1692-1661 | 32 | imported |
| 794 | Gene.FN1\|NM_054034\|2402\|1661-1692 | 32 | imported |
| 795 | Gene.FN1\|NM_212476\|8290\|1004-967 | 38 | imported |
| 796 | Gene.FN1\|NM_212476\|8290\|967-1004 | 38 | imported |
| 797 | Gene.FOXC2\|NM_005251\|1683\|305-281 | 25 | imported |
| 798 | Gene.FOXC2\|NM_005251\|1683\|281-305 | 25 | imported |
| 799 | Gene.FOXC2\|NM_005251\|1683\|783-757 | 27 | imported |
| 800 | Gene.FOXC2\|NM_005251\|1683\|757-783 | 27 | imported |
| 801 | Gene.FOXC2\|NM_005251\|1683\|1600-1569 | 32 | imported |
| 802 | Gene.FOXC2\|NM_005251\|1683\|1569-1600 | 32 | imported |
| 803 | Gene.GABARAP\|NM_007278\|924\|279-241 | 39 | imported |
| 804 | Gene.GABARAP\|NM_007278\|924\|241-279 | 39 | imported |
| 805 | Gene.GABARAP\|NM_007278\|924\|223-191 | 33 | imported |
| 806 | Gene.GABARAP\|NM_007278\|924\|191-223 | 33 | imported |
| 807 | Gene.GABARAP\|NM_007278\|924\|572-541 | 32 | imported |
| 808 | Gene.GABARAP\|NM_007278\|924\|541-572 | 32 | imported |
| 809 | Gene.GABARAPL1\|NM_031412\|1885\|254-225 | 30 | imported |
| 810 | Gene.GABARAPL1\|NM_031412\|1885\|225-254 | 30 | imported |
| 811 | Gene.GABARAPL1\|NM_031412\|1885\|1317-1281 | 37 | imported |
| 812 | Gene.GABARAPL1\|NM_031412\|1885\|1281-1317 | 37 | imported |
| 813 | Gene.GABARAPL1\|NM_031412\|1885\|665-641 | 25 | imported |
| 814 | Gene.GABARAPL1\|NM_031412\|1885\|641-665 | 25 | imported |
| 815 | Gene.GABARAPL2\|NM_007285\|1031\|515-491 | 25 | imported |
| 816 | Gene.GABARAPL2\|NM_007285\|1031\|491-515 | 25 | imported |
| 817 | Gene.GABARAPL2\|NM_007285\|1031\|547-511 | 37 | imported |
| 818 | Gene.GABARAPL2\|NM_007285\|1031\|511-547 | 37 | imported |
| 819 | Gene.GABARAPL2\|NM_007285\|1031\|821-781 | 41 | imported |
| 820 | Gene.GABARAPL2\|NM_007285\|1031\|781-821 | 41 | imported |
| 821 | Gene.GADD45A\|NM_001199741\|1296\|614-584 | 31 | imported |
| 822 | Gene.GADD45A\|NM_001199741\|1296\|584-614 | 31 | imported |
| 823 | Gene.GADD45A\|NM_001199741\|1296\|717-683 | 35 | imported |
| 824 | Gene.GADD45A\|NM_001199741\|1296\|683-717 | 35 | imported |
| 825 | Gene.GADD45A\|NM_001199741\|1296\|804-760 | 45 | imported |
| 826 | Gene.GADD45A\|NM_001199741\|1296\|760-804 | 45 | imported |
| 827 | Gene.GADD45G\|NM_006705\|1087\|132-101 | 32 | imported |
| 828 | Gene.GADD45G\|NM_006705\|1087\|101-132 | 32 | imported |
| 829 | Gene.GADD45G\|NM_006705\|1087\|195-171 | 25 | imported |
| 830 | Gene.GADD45G\|NM_006705\|1087\|171-195 | 25 | imported |
| 831 | Gene.GADD45G\|NM_006705\|1087\|257-231 | 27 | imported |
| 832 | Gene.GADD45G\|NM_006705\|1087\|231-257 | 27 | imported |
| 833 | Gene.GATA3\|NM_001002295\|3070\|133-105 | 29 | imported |
| 834 | Gene.GATA3\|NM_001002295\|3070\|105-133 | 29 | imported |
| 835 | Gene.GATA3\|NM_001002295\|3070\|936-911 | 26 | imported |
| 836 | Gene.GATA3\|NM_001002295\|3070\|911-936 | 26 | imported |
| 837 | Gene.GATA3\|NM_001002295\|3070\|2016-1977 | 40 | imported |
| 838 | Gene.GATA3\|NM_001002295\|3070\|1977-2016 | 40 | imported |
| 839 | Gene.GEMIN2\|NM_001009182\|1323\|47-23 | 25 | imported |
| 840 | Gene.GEMIN2\|NM_001009182\|1323\|23-47 | 25 | imported |
| 841 | Gene.GEMIN2\|NM_001009182\|1323\|151-122 | 30 | imported |
| 842 | Gene.GEMIN2\|NM_001009182\|1323\|122-151 | 30 | imported |
| 843 | Gene.GEMIN2\|NM_001009182\|1323\|1239-1200 | 40 | imported |
| 844 | Gene.GEMIN2\|NM_001009182\|1323\|1200-1239 | 40 | imported |
| 845 | Gene.GEN1\|NM_001130009\|6931\|266-233 | 34 | imported |
| 846 | Gene.GEN1\|NM_001130009\|6931\|233-266 | 34 | imported |
| 847 | Gene.GEN1\|NM_001130009\|6931\|799-755 | 45 | imported |
| 848 | Gene.GEN1\|NM_001130009\|6931\|755-799 | 45 | imported |
| 849 | Gene.GEN1\|NM_001130009\|6931\|5613-5569 | 45 | imported |
| 850 | Gene.GEN1\|NM_001130009\|6931\|5569-5613 | 45 | imported |
| 851 | Gene.GLI2\|NM_005270\|6780\|1170-1141 | 30 | imported |
| 852 | Gene.GLI2\|NM_005270\|6780\|1141-1170 | 30 | imported |
| 853 | Gene.GLI2\|NM_005270\|6780\|5055-5017 | 39 | imported |
| 854 | Gene.GLI2\|NM_005270\|6780\|5017-5055 | 39 | imported |
| 855 | Gene.GLI2\|NM_005270\|6780\|6020-5986 | 35 | imported |
| 856 | Gene.GLI2\|NM_005270\|6780\|5986-6020 | 35 | imported |
| 857 | Gene.GLO1\|NM_006708\|2071\|954-919 | 36 | imported |
| 858 | Gene.GLO1\|NM_006708\|2071\|919-954 | 36 | imported |
| 859 | Gene.GLO1\|NM_006708\|2071\|1309-1276 | 34 | imported |
| 860 | Gene.GLO1\|NM_006708\|2071\|1276-1309 | 34 | imported |
| 861 | Gene.GLO1\|NM_006708\|2071\|1363-1327 | 37 | imported |
| 862 | Gene.GLO1\|NM_006708\|2071\|1327-1363 | 37 | imported |
| 863 | Gene.GLS\|NM_001256310\|4509\|1522-1483 | 40 | imported |
| 864 | Gene.GLS\|NM_001256310\|4509\|1483-1522 | 40 | imported |
| 865 | Gene.GLS\|NM_001256310\|4509\|1822-1787 | 36 | imported |
| 866 | Gene.GLS\|NM_001256310\|4509\|1787-1822 | 36 | imported |
| 867 | Gene.GLS\|NM_014905\|4850\|1796-1764 | 33 | imported |
| 868 | Gene.GLS\|NM_014905\|4850\|1764-1796 | 33 | imported |
| 869 | Gene.GLS2\|NM_001280796\|2721\|1042-1013 | 30 | imported |
| 870 | Gene.GLS2\|NM_001280796\|2721\|1013-1042 | 30 | imported |
| 871 | Gene.GLS2\|NM_001280796\|2721\|1926-1887 | 40 | imported |
| 872 | Gene.GLS2\|NM_001280796\|2721\|1887-1926 | 40 | imported |
| 873 | Gene.GLS2\|NM_001280796\|2721\|2542-2508 | 35 | imported |
| 874 | Gene.GLS2\|NM_001280796\|2721\|2508-2542 | 35 | imported |
| 875 | Gene.GLUD1\|NM_001318904\|3741\|3547-3504 | 44 | imported |
| 876 | Gene.GLUD1\|NM_001318904\|3741\|3504-3547 | 44 | imported |
| 877 | Gene.GLUD1\|NM_005271\|3514\|2157-2118 | 40 | imported |
| 878 | Gene.GLUD1\|NM_005271\|3514\|2118-2157 | 40 | imported |
| 879 | Gene.GLUD1\|NM_001318905\|3867\|2590-2561 | 30 | imported |
| 880 | Gene.GLUD1\|NM_001318905\|3867\|2561-2590 | 30 | imported |
| 881 | Gene.GLUL\|NM_001033044\|7981\|1302-1274 | 29 | imported |
| 882 | Gene.GLUL\|NM_001033044\|7981\|1274-1302 | 29 | imported |
| 883 | Gene.GLUL\|NM_001033044\|7981\|1572-1542 | 31 | imported |
| 884 | Gene.GLUL\|NM_001033044\|7981\|1542-1572 | 31 | imported |
| 885 | Gene.GLUL\|NM_001033044\|7981\|3314-3284 | 31 | imported |
| 886 | Gene.GLUL\|NM_001033044\|7981\|3284-3314 | 31 | imported |
| 887 | Gene.GMPS\|NM_003875\|2457\|838-799 | 40 | imported |
| 888 | Gene.GMPS\|NM_003875\|2457\|799-838 | 40 | imported |
| 889 | Gene.GMPS\|NM_003875\|2457\|988-946 | 43 | imported |
| 890 | Gene.GMPS\|NM_003875\|2457\|946-988 | 43 | imported |
| 891 | Gene.GMPS\|NM_003875\|2457\|1466-1429 | 38 | imported |
| 892 | Gene.GMPS\|NM_003875\|2457\|1429-1466 | 38 | imported |
| 893 | Gene.H2AFX\|NM_002105\|1651\|59-29 | 31 | imported |
| 894 | Gene.H2AFX\|NM_002105\|1651\|29-59 | 31 | imported |
| 895 | Gene.H2AFX\|NM_002105\|1651\|83-57 | 27 | imported |
| 896 | Gene.H2AFX\|NM_002105\|1651\|57-83 | 27 | imported |
| 897 | Gene.H2AFX\|NM_002105\|1651\|42-15 | 28 | imported |
| 898 | Gene.H2AFX\|NM_002105\|1651\|15-42 | 28 | imported |
| 899 | Gene.HGF\|NM_000601\|6002\|183-151 | 33 | imported |
| 900 | Gene.HGF\|NM_000601\|6002\|151-183 | 33 | imported |
| 901 | Gene.HGF\|NM_001010931\|1369\|200-169 | 32 | imported |
| 902 | Gene.HGF\|NM_001010931\|1369\|169-200 | 32 | imported |
| 903 | Gene.HGF\|NM_001010933\|1354\|213-188 | 26 | imported |
| 904 | Gene.HGF\|NM_001010933\|1354\|188-213 | 26 | imported |
| 905 | Gene.HIF1A\|NM_001243084\|3979\|1429-1387 | 43 | imported |
| 906 | Gene.HIF1A\|NM_001243084\|3979\|1387-1429 | 43 | imported |
| 907 | Gene.HIF1A\|NM_001243084\|3979\|1857-1816 | 42 | imported |
| 908 | Gene.HIF1A\|NM_001243084\|3979\|1816-1857 | 42 | imported |
| 909 | Gene.HIF1A\|NM_001530\|4082\|3649-3605 | 45 | imported |
| 910 | Gene.HIF1A\|NM_001530\|4082\|3605-3649 | 45 | imported |
| 911 | Gene.HMGA2\|NM_003483\|4150\|624-596 | 29 | imported |
| 912 | Gene.HMGA2\|NM_003483\|4150\|596-624 | 29 | imported |
| 913 | Gene.HMGA2\|NM_003483\|4150\|900-876 | 25 | imported |
| 914 | Gene.HMGA2\|NM_003483\|4150\|876-900 | 25 | imported |
| 915 | Gene.HMGA2\|NM_003484\|1539\|650-625 | 26 | imported |
| 916 | Gene.HMGA2\|NM_003484\|1539\|625-650 | 26 | imported |
| 917 | Gene.HMGCR\|NM_000859\|4589\|864-837 | 28 | imported |
| 918 | Gene.HMGCR\|NM_000859\|4589\|837-864 | 28 | imported |
| 919 | Gene.HMGCR\|NM_000859\|4589\|3042-3003 | 40 | imported |
| 920 | Gene.HMGCR\|NM_000859\|4589\|3003-3042 | 40 | imported |
| 921 | Gene.HMGCR\|NM_000859\|4589\|4101-4067 | 35 | imported |
| 922 | Gene.HMGCR\|NM_000859\|4589\|4067-4101 | 35 | imported |
| 923 | Gene.HSP90AA1\|NM_001017963\|3887\|3728-3697 | 32 | imported |
| 924 | Gene.HSP90AA1\|NM_001017963\|3887\|3697-3728 | 32 | imported |
| 925 | Gene.HSP90AA1\|NM_001017963\|3887\|3759-3730 | 30 | imported |
| 926 | Gene.HSP90AA1\|NM_001017963\|3887\|3730-3759 | 30 | imported |
| 927 | Gene.HSP90AA1\|NM_005348\|3366\|3348-3305 | 44 | imported |
| 928 | Gene.HSP90AA1\|NM_005348\|3366\|3305-3348 | 44 | imported |
| 929 | Gene.HSP90B1\|NM_003299\|2879\|73-49 | 25 | imported |
| 930 | Gene.HSP90B1\|NM_003299\|2879\|49-73 | 25 | imported |
| 931 | Gene.HSP90B1\|NM_003299\|2879\|2118-2089 | 30 | imported |
| 932 | Gene.HSP90B1\|NM_003299\|2879\|2089-2118 | 30 | imported |
| 933 | Gene.HSP90B1\|NM_003299\|2879\|2436-2401 | 36 | imported |
| 934 | Gene.HSP90B1\|NM_003299\|2879\|2401-2436 | 36 | imported |
| 935 | Gene.HSPA5\|NM_005347\|3973\|820-793 | 28 | imported |
| 936 | Gene.HSPA5\|NM_005347\|3973\|793-820 | 28 | imported |
| 937 | Gene.HSPA5\|NM_005347\|3973\|865-826 | 40 | imported |
| 938 | Gene.HSPA5\|NM_005347\|3973\|826-865 | 40 | imported |
| 939 | Gene.HSPA5\|NM_005347\|3973\|3704-3664 | 41 | imported |
| 940 | Gene.HSPA5\|NM_005347\|3973\|3664-3704 | 41 | imported |
| 941 | Gene.HSPA8\|NM_006597\|2473\|431-400 | 32 | imported |
| 942 | Gene.HSPA8\|NM_006597\|2473\|400-431 | 32 | imported |
| 943 | Gene.HSPA8\|NM_006597\|2473\|1523-1492 | 32 | imported |
| 944 | Gene.HSPA8\|NM_006597\|2473\|1492-1523 | 32 | imported |
| 945 | Gene.HSPA8\|NM_153201\|2014\|762-732 | 31 | imported |
| 946 | Gene.HSPA8\|NM_153201\|2014\|732-762 | 31 | imported |
| 947 | Gene.HUS1\|NM_004507\|3033\|236-201 | 36 | imported |
| 948 | Gene.HUS1\|NM_004507\|3033\|201-236 | 36 | imported |
| 949 | Gene.HUS1\|NM_004507\|3033\|815-776 | 40 | imported |
| 950 | Gene.HUS1\|NM_004507\|3033\|776-815 | 40 | imported |
| 951 | Gene.HUS1\|NM_004507\|3033\|1052-1026 | 27 | imported |
| 952 | Gene.HUS1\|NM_004507\|3033\|1026-1052 | 27 | imported |
| 953 | Gene.CHEK1\|NM_001114121\|2699\|1377-1335 | 43 | imported |
| 954 | Gene.CHEK1\|NM_001114121\|2699\|1335-1377 | 43 | imported |
| 955 | Gene.CHEK1\|NM_001114121\|2699\|1546-1519 | 28 | imported |
| 956 | Gene.CHEK1\|NM_001114121\|2699\|1519-1546 | 28 | imported |
| 957 | Gene.CHEK1\|NM_001114121\|2699\|1898-1864 | 35 | imported |
| 958 | Gene.CHEK1\|NM_001114121\|2699\|1864-1898 | 35 | imported |
| 959 | Gene.CHEK2\|NM_001005735\|1991\|114-86 | 29 | imported |
| 960 | Gene.CHEK2\|NM_001005735\|1991\|86-114 | 29 | imported |
| 961 | Gene.CHEK2\|NM_001005735\|1991\|721-681 | 41 | imported |
| 962 | Gene.CHEK2\|NM_001005735\|1991\|681-721 | 41 | imported |
| 963 | Gene.CHEK2\|NM_001257387\|1976\|1140-1106 | 35 | imported |
| 964 | Gene.CHEK2\|NM_001257387\|1976\|1106-1140 | 35 | imported |
| 965 | Gene.ICAM1\|NM_000201\|3249\|520-487 | 34 | imported |
| 966 | Gene.ICAM1\|NM_000201\|3249\|487-520 | 34 | imported |
| 967 | Gene.ICAM1\|NM_000201\|3249\|1107-1081 | 27 | imported |
| 968 | Gene.ICAM1\|NM_000201\|3249\|1081-1107 | 27 | imported |
| 969 | Gene.ICAM1\|NM_000201\|3249\|2141-2107 | 35 | imported |
| 970 | Gene.ICAM1\|NM_000201\|3249\|2107-2141 | 35 | imported |
| 971 | Gene.IDH3A\|NM_005530\|2701\|948-921 | 28 | imported |
| 972 | Gene.IDH3A\|NM_005530\|2701\|921-948 | 28 | imported |
| 973 | Gene.IDH3A\|NM_005530\|2701\|995-967 | 29 | imported |
| 974 | Gene.IDH3A\|NM_005530\|2701\|967-995 | 29 | imported |
| 975 | Gene.IDH3A\|NM_005530\|2701\|1018-990 | 29 | imported |
| 976 | Gene.IDH3A\|NM_005530\|2701\|990-1018 | 29 | imported |
| 977 | Gene.IDO1\|NM_002164\|1944\|97-65 | 33 | imported |
| 978 | Gene.IDO1\|NM_002164\|1944\|65-97 | 33 | imported |
| 979 | Gene.IDO1\|NM_002164\|1944\|187-145 | 43 | imported |
| 980 | Gene.IDO1\|NM_002164\|1944\|145-187 | 43 | imported |
| 981 | Gene.IDO1\|NM_002164\|1944\|1263-1233 | 31 | imported |
| 982 | Gene.IDO1\|NM_002164\|1944\|1233-1263 | 31 | imported |
| 983 | Gene.IFNG\|NM_000619\|1240\|580-551 | 30 | imported |
| 984 | Gene.IFNG\|NM_000619\|1240\|551-580 | 30 | imported |
| 985 | Gene.IFNG\|NM_000619\|1240\|604-571 | 34 | imported |
| 986 | Gene.IFNG\|NM_000619\|1240\|571-604 | 34 | imported |
| 987 | Gene.IFNG\|NM_000619\|1240\|622-591 | 32 | imported |
| 988 | Gene.IFNG\|NM_000619\|1240\|591-622 | 32 | imported |
| 989 | Gene.IGF1\|NM_000618\|7366\|2136-2109 | 28 | imported |
| 990 | Gene.IGF1\|NM_000618\|7366\|2109-2136 | 28 | imported |
| 991 | Gene.IGF1\|NM_001111284\|7204\|334-301 | 34 | imported |
| 992 | Gene.IGF1\|NM_001111284\|7204\|301-334 | 34 | imported |
| 993 | Gene.IGF1\|NM_001111285\|1188\|517-491 | 27 | imported |
| 994 | Gene.IGF1\|NM_001111285\|1188\|491-517 | 27 | imported |
| 995 | Gene.IGF1R\|NM_000875\|12262\|2784-2755 | 30 | imported |
| 996 | Gene.IGF1R\|NM_000875\|12262\|2755-2784 | 30 | imported |
| 997 | Gene.IGF1R\|NM_000875\|12262\|4010-3979 | 32 | imported |
| 998 | Gene.IGF1R\|NM_000875\|12262\|3979-4010 | 32 | imported |
| 999 | Gene.IGF1R\|NM_000875\|12262\|10134-10099 | 36 | imported |
| 1000 | Gene.IGF1R\|NM_000875\|12262\|10099-10134 | 36 | imported |
| 1001 | Gene.IGF2\|NM_000612\|5188\|3078-3054 | 25 | imported |
| 1002 | Gene.IGF2\|NM_000612\|5188\|3054-3078 | 25 | imported |
| 1003 | Gene.IGF2\|NM_000612\|5188\|4067-4043 | 25 | imported |
| 1004 | Gene.IGF2\|NM_000612\|5188\|4043-4067 | 25 | imported |
| 1005 | Gene.IGF2\|NM_001007139\|5162\|928-904 | 25 | imported |
| 1006 | Gene.IGF2\|NM_001007139\|5162\|904-928 | 25 | imported |
| 1007 | Gene.IGFBP4\|NM_001552\|2246\|139-115 | 25 | imported |
| 1008 | Gene.IGFBP4\|NM_001552\|2246\|115-139 | 25 | imported |
| 1009 | Gene.IGFBP4\|NM_001552\|2246\|1630-1597 | 34 | imported |
| 1010 | Gene.IGFBP4\|NM_001552\|2246\|1597-1630 | 34 | imported |
| 1011 | Gene.IGFBP4\|NM_001552\|2246\|1857-1825 | 33 | imported |
| 1012 | Gene.IGFBP4\|NM_001552\|2246\|1825-1857 | 33 | imported |
| 1013 | Gene.IL6\|NM_000600\|1197\|142-111 | 32 | imported |
| 1014 | Gene.IL6\|NM_000600\|1197\|111-142 | 32 | imported |
| 1015 | Gene.IL6\|NM_000600\|1197\|380-351 | 30 | imported |
| 1016 | Gene.IL6\|NM_000600\|1197\|351-380 | 30 | imported |
| 1017 | Gene.IL6\|NM_000600\|1197\|456-421 | 36 | imported |
| 1018 | Gene.IL6\|NM_000600\|1197\|421-456 | 36 | imported |
| 1019 | Gene.IL6R\|NM_000565\|5928\|1084-1051 | 34 | imported |
| 1020 | Gene.IL6R\|NM_000565\|5928\|1051-1084 | 34 | imported |
| 1021 | Gene.IL6R\|NM_001206866\|2058\|902-868 | 35 | imported |
| 1022 | Gene.IL6R\|NM_001206866\|2058\|868-902 | 35 | imported |
| 1023 | Gene.IL6R\|NM_001206866\|2058\|1148-1123 | 26 | imported |
| 1024 | Gene.IL6R\|NM_001206866\|2058\|1123-1148 | 26 | imported |
| 1025 | Gene.ILK\|NM_001014794\|1797\|1036-1006 | 31 | imported |
| 1026 | Gene.ILK\|NM_001014794\|1797\|1006-1036 | 31 | imported |
| 1027 | Gene.ILK\|NM_001014795\|2098\|1094-1063 | 32 | imported |
| 1028 | Gene.ILK\|NM_001014795\|2098\|1063-1094 | 32 | imported |
| 1029 | Gene.ILK\|NM_001014795\|2098\|1556-1531 | 26 | imported |
| 1030 | Gene.ILK\|NM_001014795\|2098\|1531-1556 | 26 | imported |
| 1031 | Gene.IRGM\|NM_001145805\|1659\|792-757 | 36 | imported |
| 1032 | Gene.IRGM\|NM_001145805\|1659\|757-792 | 36 | imported |
| 1033 | Gene.IRGM\|NM_001145805\|1659\|1264-1233 | 32 | imported |
| 1034 | Gene.IRGM\|NM_001145805\|1659\|1233-1264 | 32 | imported |
| 1035 | Gene.IRGM\|NM_001145805\|1659\|1431-1401 | 31 | imported |
| 1036 | Gene.IRGM\|NM_001145805\|1659\|1401-1431 | 31 | imported |
| 1037 | Gene.ITGA1\|NM_181501\|4811\|3235-3201 | 35 | imported |
| 1038 | Gene.ITGA1\|NM_181501\|4811\|3201-3235 | 35 | imported |
| 1039 | Gene.ITGA1\|NM_181501\|4811\|3510-3481 | 30 | imported |
| 1040 | Gene.ITGA1\|NM_181501\|4811\|3481-3510 | 30 | imported |
| 1041 | Gene.ITGA1\|NM_181501\|4811\|4437-4401 | 37 | imported |
| 1042 | Gene.ITGA1\|NM_181501\|4811\|4401-4437 | 37 | imported |
| 1043 | Gene.ITGB3\|NM_000212\|4894\|519-493 | 27 | imported |
| 1044 | Gene.ITGB3\|NM_000212\|4894\|493-519 | 27 | imported |
| 1045 | Gene.ITGB3\|NM_000212\|4894\|2870-2830 | 41 | imported |
| 1046 | Gene.ITGB3\|NM_000212\|4894\|2830-2870 | 41 | imported |
| 1047 | Gene.ITGB3\|NM_000212\|4894\|3070-3035 | 36 | imported |
| 1048 | Gene.ITGB3\|NM_000212\|4894\|3035-3070 | 36 | imported |
| 1049 | Gene.JAG1\|NM_000214\|5988\|2039-2001 | 39 | imported |
| 1050 | Gene.JAG1\|NM_000214\|5988\|2001-2039 | 39 | imported |
| 1051 | Gene.JAG1\|NM_000214\|5988\|2826-2801 | 26 | imported |
| 1052 | Gene.JAG1\|NM_000214\|5988\|2801-2826 | 26 | imported |
| 1053 | Gene.JAG1\|NM_000214\|5988\|5783-5751 | 33 | imported |
| 1054 | Gene.JAG1\|NM_000214\|5988\|5751-5783 | 33 | imported |
| 1055 | Gene.KDR\|NM_002253\|6055\|902-868 | 35 | imported |
| 1056 | Gene.KDR\|NM_002253\|6055\|868-902 | 35 | imported |
| 1057 | Gene.KDR\|NM_002253\|6055\|2323-2296 | 28 | imported |
| 1058 | Gene.KDR\|NM_002253\|6055\|2296-2323 | 28 | imported |
| 1059 | Gene.KDR\|NM_002253\|6055\|5130-5101 | 30 | imported |
| 1060 | Gene.KDR\|NM_002253\|6055\|5101-5130 | 30 | imported |
| 1061 | Gene.KLF4\|NM_004235\|2903\|145-121 | 25 | imported |
| 1062 | Gene.KLF4\|NM_004235\|2903\|121-145 | 25 | imported |
| 1063 | Gene.KLF4\|NM_004235\|2903\|1784-1753 | 32 | imported |
| 1064 | Gene.KLF4\|NM_004235\|2903\|1753-1784 | 32 | imported |
| 1065 | Gene.KLF4\|NM_004235\|2903\|1805-1777 | 29 | imported |
| 1066 | Gene.KLF4\|NM_004235\|2903\|1777-1805 | 29 | imported |
| 1067 | Gene.KLRC3\|NM_002261\|1042\|677-641 | 37 | imported |
| 1068 | Gene.KLRC3\|NM_002261\|1042\|641-677 | 37 | imported |
| 1069 | Gene.KLRC3\|NM_002261\|1042\|700-661 | 40 | imported |
| 1070 | Gene.KLRC3\|NM_002261\|1042\|661-700 | 40 | imported |
| 1071 | Gene.KLRC3\|NM_002261\|1042\|380-351 | 30 | imported |
| 1072 | Gene.KLRC3\|NM_002261\|1042\|351-380 | 30 | imported |
| 1073 | Gene.KRAS\|NM_004985\|5765\|573-529 | 45 | imported |
| 1074 | Gene.KRAS\|NM_004985\|5765\|529-573 | 45 | imported |
| 1075 | Gene.KRAS\|NM_004985\|5765\|1760-1729 | 32 | imported |
| 1076 | Gene.KRAS\|NM_004985\|5765\|1729-1760 | 32 | imported |
| 1077 | Gene.KRAS\|NM_033360\|5889\|1758-1716 | 43 | imported |
| 1078 | Gene.KRAS\|NM_033360\|5889\|1716-1758 | 43 | imported |
| 1079 | Gene.KRT14\|NM_000526\|1653\|1430-1401 | 30 | imported |
| 1080 | Gene.KRT14\|NM_000526\|1653\|1401-1430 | 30 | imported |
| 1081 | Gene.KRT14\|NM_000526\|1653\|1454-1429 | 26 | imported |
| 1082 | Gene.KRT14\|NM_000526\|1653\|1429-1454 | 26 | imported |
| 1083 | Gene.KRT14\|NM_000526\|1653\|1485-1457 | 29 | imported |
| 1084 | Gene.KRT14\|NM_000526\|1653\|1457-1485 | 29 | imported |
| 1085 | Gene.KRT7\|NM_005556\|1753\|828-796 | 33 | imported |
| 1086 | Gene.KRT7\|NM_005556\|1753\|796-828 | 33 | imported |
| 1087 | Gene.KRT7\|NM_005556\|1753\|1347-1321 | 27 | imported |
| 1088 | Gene.KRT7\|NM_005556\|1753\|1321-1347 | 27 | imported |
| 1089 | Gene.KRT7\|NM_005556\|1753\|1687-1651 | 37 | imported |
| 1090 | Gene.KRT7\|NM_005556\|1753\|1651-1687 | 37 | imported |
| 1091 | Gene.LCN2\|NM_005564\|863\|220-191 | 30 | imported |
| 1092 | Gene.LCN2\|NM_005564\|863\|191-220 | 30 | imported |
| 1093 | Gene.LCN2\|NM_005564\|863\|127-101 | 27 | imported |
| 1094 | Gene.LCN2\|NM_005564\|863\|101-127 | 27 | imported |
| 1095 | Gene.LCN2\|NM_005564\|863\|95-71 | 25 | imported |
| 1096 | Gene.LCN2\|NM_005564\|863\|71-95 | 25 | imported |
| 1097 | Gene.LIG4\|NM_001098268\|4077\|2343-2313 | 31 | imported |
| 1098 | Gene.LIG4\|NM_001098268\|4077\|2313-2343 | 31 | imported |
| 1099 | Gene.LIG4\|NM_001098268\|4077\|2454-2415 | 40 | imported |
| 1100 | Gene.LIG4\|NM_001098268\|4077\|2415-2454 | 40 | imported |
| 1101 | Gene.LIG4\|NM_001098268\|4077\|2789-2755 | 35 | imported |
| 1102 | Gene.LIG4\|NM_001098268\|4077\|2755-2789 | 35 | imported |
| 1103 | Gene.LIN28A\|NM_024674\|4024\|268-239 | 30 | imported |
| 1104 | Gene.LIN28A\|NM_024674\|4024\|239-268 | 30 | imported |
| 1105 | Gene.LIN28A\|NM_024674\|4024\|643-613 | 31 | imported |
| 1106 | Gene.LIN28A\|NM_024674\|4024\|613-643 | 31 | imported |
| 1107 | Gene.LIN28A\|NM_024674\|4024\|2082-2041 | 42 | imported |
| 1108 | Gene.LIN28A\|NM_024674\|4024\|2041-2082 | 42 | imported |
| 1109 | Gene.LTF\|NM_001199149\|2537\|2067-2038 | 30 | imported |
| 1110 | Gene.LTF\|NM_001199149\|2537\|2038-2067 | 30 | imported |
| 1111 | Gene.LTF\|NM_001199149\|2537\|2156-2122 | 35 | imported |
| 1112 | Gene.LTF\|NM_001199149\|2537\|2122-2156 | 35 | imported |
| 1113 | Gene.LTF\|NM_002343\|2648\|1684-1651 | 34 | imported |
| 1114 | Gene.LTF\|NM_002343\|2648\|1651-1684 | 34 | imported |
| 1115 | Gene.MAP1LC3A\|NM_032514\|1048\|250-221 | 30 | imported |
| 1116 | Gene.MAP1LC3A\|NM_032514\|1048\|221-250 | 30 | imported |
| 1117 | Gene.MAP1LC3A\|NM_032514\|1048\|280-251 | 30 | imported |
| 1118 | Gene.MAP1LC3A\|NM_032514\|1048\|251-280 | 30 | imported |
| 1119 | Gene.MAP1LC3A\|NM_032514\|1048\|385-361 | 25 | imported |
| 1120 | Gene.MAP1LC3A\|NM_032514\|1048\|361-385 | 25 | imported |
| 1121 | Gene.MAP1LC3B\|NM_022818\|2304\|1211-1179 | 33 | imported |
| 1122 | Gene.MAP1LC3B\|NM_022818\|2304\|1179-1211 | 33 | imported |
| 1123 | Gene.MAP1LC3B\|NM_022818\|2304\|1422-1388 | 35 | imported |
| 1124 | Gene.MAP1LC3B\|NM_022818\|2304\|1388-1422 | 35 | imported |
| 1125 | Gene.MAP1LC3B\|NM_022818\|2304\|1993-1958 | 36 | imported |
| 1126 | Gene.MAP1LC3B\|NM_022818\|2304\|1958-1993 | 36 | imported |
| 1127 | Gene.MDC1\|NM_014641\|7385\|4002-3969 | 34 | imported |
| 1128 | Gene.MDC1\|NM_014641\|7385\|3969-4002 | 34 | imported |
| 1129 | Gene.MDC1\|NM_014641\|7385\|5369-5333 | 37 | imported |
| 1130 | Gene.MDC1\|NM_014641\|7385\|5333-5369 | 37 | imported |
| 1131 | Gene.MDC1\|NM_014641\|7385\|6543-6511 | 33 | imported |
| 1132 | Gene.MDC1\|NM_014641\|7385\|6511-6543 | 33 | imported |
| 1133 | Gene.MDK\|NM_001012333\|969\|206-181 | 26 | imported |
| 1134 | Gene.MDK\|NM_001012333\|969\|181-206 | 26 | imported |
| 1135 | Gene.MDK\|NM_001012333\|969\|746-711 | 36 | imported |
| 1136 | Gene.MDK\|NM_001012333\|969\|711-746 | 36 | imported |
| 1137 | Gene.MDK\|NM_001012334\|1015\|838-811 | 28 | imported |
| 1138 | Gene.MDK\|NM_001012334\|1015\|811-838 | 28 | imported |
| 1139 | Gene.MIF\|NM_002415\|561\|131-101 | 31 | imported |
| 1140 | Gene.MIF\|NM_002415\|561\|101-131 | 31 | imported |
| 1141 | Gene.MIF\|NM_002415\|561\|255-231 | 25 | imported |
| 1142 | Gene.MIF\|NM_002415\|561\|231-255 | 25 | imported |
| 1143 | Gene.MIF\|NM_002415\|561\|440-411 | 30 | imported |
| 1144 | Gene.MIF\|NM_002415\|561\|411-440 | 30 | imported |
| 1145 | Gene.MKI67\|NM_001145966\|11427\|3546-3516 | 31 | imported |
| 1146 | Gene.MKI67\|NM_001145966\|11427\|3516-3546 | 31 | imported |
| 1147 | Gene.MKI67\|NM_001145966\|11427\|10679-10641 | 39 | imported |
| 1148 | Gene.MKI67\|NM_001145966\|11427\|10641-10679 | 39 | imported |
| 1149 | Gene.MKI67\|NM_002417\|12507\|10433-10401 | 33 | imported |
| 1150 | Gene.MKI67\|NM_002417\|12507\|10401-10433 | 33 | imported |
| 1151 | Gene.MMP2\|NM_001127891\|3416\|1882-1857 | 26 | imported |
| 1152 | Gene.MMP2\|NM_001127891\|3416\|1857-1882 | 26 | imported |
| 1153 | Gene.MMP2\|NM_001127891\|3416\|2354-2321 | 34 | imported |
| 1154 | Gene.MMP2\|NM_001127891\|3416\|2321-2354 | 34 | imported |
| 1155 | Gene.MMP2\|NM_001127891\|3416\|3171-3133 | 39 | imported |
| 1156 | Gene.MMP2\|NM_001127891\|3416\|3133-3171 | 39 | imported |
| 1157 | Gene.MMP9\|NM_004994\|2387\|506-481 | 26 | imported |
| 1158 | Gene.MMP9\|NM_004994\|2387\|481-506 | 26 | imported |
| 1159 | Gene.MMP9\|NM_004994\|2387\|1174-1141 | 34 | imported |
| 1160 | Gene.MMP9\|NM_004994\|2387\|1141-1174 | 34 | imported |
| 1161 | Gene.MMP9\|NM_004994\|2387\|1386-1361 | 26 | imported |
| 1162 | Gene.MMP9\|NM_004994\|2387\|1361-1386 | 26 | imported |
| 1163 | Gene.MRE11A\|NM_005590\|5164\|4216-4172 | 45 | imported |
| 1164 | Gene.MRE11A\|NM_005590\|5164\|4172-4216 | 45 | imported |
| 1165 | Gene.MRE11A\|NM_005590\|5164\|4515-4473 | 43 | imported |
| 1166 | Gene.MRE11A\|NM_005590\|5164\|4473-4515 | 43 | imported |
| 1167 | Gene.MRE11A\|NM_005591\|5141\|2099-2065 | 35 | imported |
| 1168 | Gene.MRE11A\|NM_005591\|5141\|2065-2099 | 35 | imported |
| 1169 | Gene.MST1R\|NM_001244937\|4402\|1544-1518 | 27 | imported |
| 1170 | Gene.MST1R\|NM_001244937\|4402\|1518-1544 | 27 | imported |
| 1171 | Gene.MST1R\|NM_001244937\|4402\|2100-2073 | 28 | imported |
| 1172 | Gene.MST1R\|NM_001244937\|4402\|2073-2100 | 28 | imported |
| 1173 | Gene.MST1R\|NM_001244937\|4402\|3578-3553 | 26 | imported |
| 1174 | Gene.MST1R\|NM_001244937\|4402\|3553-3578 | 26 | imported |
| 1175 | Gene.MT1A\|NM_005946\|468\|308-271 | 38 | imported |
| 1176 | Gene.MT1A\|NM_005946\|468\|271-308 | 38 | imported |
| 1177 | Gene.MT1A\|NM_005946\|468\|42-11 | 32 | imported |
| 1178 | Gene.MT1A\|NM_005946\|468\|11-42 | 32 | imported |
| 1179 | Gene.MT1A\|NM_005946\|468\|67-41 | 27 | imported |
| 1180 | Gene.MT1A\|NM_005946\|468\|41-67 | 27 | imported |
| 1181 | Gene.MT1G\|NM_001301267\|423\|25-1 | 25 | imported |
| 1182 | Gene.MT1G\|NM_001301267\|423\|1-25 | 25 | imported |
| 1183 | Gene.MT1G\|NM_001301267\|423\|115-91 | 25 | imported |
| 1184 | Gene.MT1G\|NM_001301267\|423\|91-115 | 25 | imported |
| 1185 | Gene.MT1G\|NM_001301267\|423\|69-41 | 29 | imported |
| 1186 | Gene.MT1G\|NM_001301267\|423\|41-69 | 29 | imported |
| 1187 | Gene.MT2A\|NM_005953\|466\|76-51 | 26 | imported |
| 1188 | Gene.MT2A\|NM_005953\|466\|51-76 | 26 | imported |
| 1189 | Gene.MT2A\|NM_005953\|466\|108-81 | 28 | imported |
| 1190 | Gene.MT2A\|NM_005953\|466\|81-108 | 28 | imported |
| 1191 | Gene.MT2A\|NM_005953\|466\|95-71 | 25 | imported |
| 1192 | Gene.MT2A\|NM_005953\|466\|71-95 | 25 | imported |
| 1193 | Gene.MT3\|NM_005954\|599\|216-191 | 26 | imported |
| 1194 | Gene.MT3\|NM_005954\|599\|191-216 | 26 | imported |
| 1195 | Gene.MT3\|NM_005954\|599\|318-291 | 28 | imported |
| 1196 | Gene.MT3\|NM_005954\|599\|291-318 | 28 | imported |
| 1197 | Gene.MT3\|NM_005954\|599\|397-371 | 27 | imported |
| 1198 | Gene.MT3\|NM_005954\|599\|371-397 | 27 | imported |
| 1199 | Gene.MTF1\|NM_005955\|7986\|4794-4758 | 37 | imported |
| 1200 | Gene.MTF1\|NM_005955\|7986\|4758-4794 | 37 | imported |
| 1201 | Gene.MTF1\|NM_005955\|7986\|7071-7036 | 36 | imported |
| 1202 | Gene.MTF1\|NM_005955\|7986\|7036-7071 | 36 | imported |
| 1203 | Gene.MTF1\|NM_005955\|7986\|7399-7371 | 29 | imported |
| 1204 | Gene.MTF1\|NM_005955\|7986\|7371-7399 | 29 | imported |
| 1205 | Gene.MTHFD2\|NM_006636\|2208\|223-191 | 33 | imported |
| 1206 | Gene.MTHFD2\|NM_006636\|2208\|191-223 | 33 | imported |
| 1207 | Gene.MTHFD2\|NM_006636\|2208\|734-704 | 31 | imported |
| 1208 | Gene.MTHFD2\|NM_006636\|2208\|704-734 | 31 | imported |
| 1209 | Gene.MTHFD2\|NM_006636\|2208\|1306-1274 | 33 | imported |
| 1210 | Gene.MTHFD2\|NM_006636\|2208\|1274-1306 | 33 | imported |
| 1211 | Gene.MUS81\|NM_025128\|2406\|425-401 | 25 | imported |
| 1212 | Gene.MUS81\|NM_025128\|2406\|401-425 | 25 | imported |
| 1213 | Gene.MUS81\|NM_025128\|2406\|808-781 | 28 | imported |
| 1214 | Gene.MUS81\|NM_025128\|2406\|781-808 | 28 | imported |
| 1215 | Gene.MUS81\|NM_025128\|2406\|2218-2181 | 38 | imported |
| 1216 | Gene.MUS81\|NM_025128\|2406\|2181-2218 | 38 | imported |
| 1217 | Gene.MYCN\|NM_001293228\|3046\|1982-1951 | 32 | imported |
| 1218 | Gene.MYCN\|NM_001293228\|3046\|1951-1982 | 32 | imported |
| 1219 | Gene.MYCN\|NM_001293228\|3046\|2035-2003 | 33 | imported |
| 1220 | Gene.MYCN\|NM_001293228\|3046\|2003-2035 | 33 | imported |
| 1221 | Gene.MYCN\|NM_001293228\|3046\|2315-2289 | 27 | imported |
| 1222 | Gene.MYCN\|NM_001293228\|3046\|2289-2315 | 27 | imported |
| 1223 | Gene.MYH10\|NM_001256012\|7778\|3611-3576 | 36 | imported |
| 1224 | Gene.MYH10\|NM_001256012\|7778\|3576-3611 | 36 | imported |
| 1225 | Gene.MYH10\|NM_001256012\|7778\|5168-5136 | 33 | imported |
| 1226 | Gene.MYH10\|NM_001256012\|7778\|5136-5168 | 33 | imported |
| 1227 | Gene.MYH10\|NM_001256095\|7712\|1064-1025 | 40 | imported |
| 1228 | Gene.MYH10\|NM_001256095\|7712\|1025-1064 | 40 | imported |
| 1229 | Gene.MYH9\|NM_002473\|7554\|665-631 | 35 | imported |
| 1230 | Gene.MYH9\|NM_002473\|7554\|631-665 | 35 | imported |
| 1231 | Gene.MYH9\|NM_002473\|7554\|2550-2521 | 30 | imported |
| 1232 | Gene.MYH9\|NM_002473\|7554\|2521-2550 | 30 | imported |
| 1233 | Gene.MYH9\|NM_002473\|7554\|6648-6616 | 33 | imported |
| 1234 | Gene.MYH9\|NM_002473\|7554\|6616-6648 | 33 | imported |
| 1235 | Gene.MYLK\|NM_053025\|7852\|5841-5809 | 33 | imported |
| 1236 | Gene.MYLK\|NM_053025\|7852\|5809-5841 | 33 | imported |
| 1237 | Gene.MYLK\|NM_053026\|7645\|6445-6401 | 45 | imported |
| 1238 | Gene.MYLK\|NM_053026\|7645\|6401-6445 | 45 | imported |
| 1239 | Gene.MYLK\|NM_053031\|2600\|736-705 | 32 | imported |
| 1240 | Gene.MYLK\|NM_053031\|2600\|705-736 | 32 | imported |
| 1241 | Gene.MYO10\|NM_012334\|11436\|2309-2281 | 29 | imported |
| 1242 | Gene.MYO10\|NM_012334\|11436\|2281-2309 | 29 | imported |
| 1243 | Gene.MYO10\|NM_012334\|11436\|4971-4941 | 31 | imported |
| 1244 | Gene.MYO10\|NM_012334\|11436\|4941-4971 | 31 | imported |
| 1245 | Gene.MYO10\|NM_012334\|11436\|6883-6841 | 43 | imported |
| 1246 | Gene.MYO10\|NM_012334\|11436\|6841-6883 | 43 | imported |
| 1247 | Gene.MYO18A\|NM_078471\|7591\|469-442 | 28 | imported |
| 1248 | Gene.MYO18A\|NM_078471\|7591\|442-469 | 28 | imported |
| 1249 | Gene.MYO18A\|NM_078471\|7591\|2172-2143 | 30 | imported |
| 1250 | Gene.MYO18A\|NM_078471\|7591\|2143-2172 | 30 | imported |
| 1251 | Gene.MYO18A\|NM_078471\|7591\|4760-4726 | 35 | imported |
| 1252 | Gene.MYO18A\|NM_078471\|7591\|4726-4760 | 35 | imported |
| 1253 | Gene.MYO1B\|NM_001130158\|5218\|1535-1497 | 39 | imported |
| 1254 | Gene.MYO1B\|NM_001130158\|5218\|1497-1535 | 39 | imported |
| 1255 | Gene.MYO1B\|NM_001130158\|5218\|2716-2685 | 32 | imported |
| 1256 | Gene.MYO1B\|NM_001130158\|5218\|2685-2716 | 32 | imported |
| 1257 | Gene.MYO1B\|NM_001161819\|5203\|903-861 | 43 | imported |
| 1258 | Gene.MYO1B\|NM_001161819\|5203\|861-903 | 43 | imported |
| 1259 | Gene.MYO9B\|NM_001130065\|7652\|1886-1857 | 30 | imported |
| 1260 | Gene.MYO9B\|NM_001130065\|7652\|1857-1886 | 30 | imported |
| 1261 | Gene.MYO9B\|NM_001130065\|7652\|5274-5249 | 26 | imported |
| 1262 | Gene.MYO9B\|NM_001130065\|7652\|5249-5274 | 26 | imported |
| 1263 | Gene.MYO9B\|NM_001130065\|7652\|7321-7297 | 25 | imported |
| 1264 | Gene.MYO9B\|NM_001130065\|7652\|7297-7321 | 25 | imported |
| 1265 | Gene.NABP2\|NM_024068\|1404\|256-229 | 28 | imported |
| 1266 | Gene.NABP2\|NM_024068\|1404\|229-256 | 28 | imported |
| 1267 | Gene.NABP2\|NM_024068\|1404\|332-301 | 32 | imported |
| 1268 | Gene.NABP2\|NM_024068\|1404\|301-332 | 32 | imported |
| 1269 | Gene.NABP2\|NM_024068\|1404\|374-337 | 38 | imported |
| 1270 | Gene.NABP2\|NM_024068\|1404\|337-374 | 38 | imported |
| 1271 | Gene.NANOG\|NM_024865\|2103\|53-19 | 35 | imported |
| 1272 | Gene.NANOG\|NM_024865\|2103\|19-53 | 35 | imported |
| 1273 | Gene.NANOG\|NM_024865\|2103\|292-253 | 40 | imported |
| 1274 | Gene.NANOG\|NM_024865\|2103\|253-292 | 40 | imported |
| 1275 | Gene.NANOG\|NM_024865\|2103\|952-919 | 34 | imported |
| 1276 | Gene.NANOG\|NM_024865\|2103\|919-952 | 34 | imported |
| 1277 | Gene.NBN\|NM_002485\|4639\|851-820 | 32 | imported |
| 1278 | Gene.NBN\|NM_002485\|4639\|820-851 | 32 | imported |
| 1279 | Gene.NBN\|NM_002485\|4639\|1095-1054 | 42 | imported |
| 1280 | Gene.NBN\|NM_002485\|4639\|1054-1095 | 42 | imported |
| 1281 | Gene.NBN\|NM_002485\|4639\|3282-3238 | 45 | imported |
| 1282 | Gene.NBN\|NM_002485\|4639\|3238-3282 | 45 | imported |
| 1283 | Gene.NDRG1\|NM_001258433\|3017\|1075-1051 | 25 | imported |
| 1284 | Gene.NDRG1\|NM_001258433\|3017\|1051-1075 | 25 | imported |
| 1285 | Gene.NDRG1\|NM_001258433\|3017\|1276-1251 | 26 | imported |
| 1286 | Gene.NDRG1\|NM_001258433\|3017\|1251-1276 | 26 | imported |
| 1287 | Gene.NDRG1\|NM_001258433\|3017\|1653-1626 | 28 | imported |
| 1288 | Gene.NDRG1\|NM_001258433\|3017\|1626-1653 | 28 | imported |
| 1289 | Gene.NFE2L2\|NM_001145412\|2988\|1739-1701 | 39 | imported |
| 1290 | Gene.NFE2L2\|NM_001145412\|2988\|1701-1739 | 39 | imported |
| 1291 | Gene.NFE2L2\|NM_001313900\|2862\|2100-2065 | 36 | imported |
| 1292 | Gene.NFE2L2\|NM_001313900\|2862\|2065-2100 | 36 | imported |
| 1293 | Gene.NFE2L2\|NM_001313904\|2917\|2684-2641 | 44 | imported |
| 1294 | Gene.NFE2L2\|NM_001313904\|2917\|2641-2684 | 44 | imported |
| 1295 | Gene.NFKB1\|NM_001165412\|4090\|3738-3707 | 32 | imported |
| 1296 | Gene.NFKB1\|NM_001165412\|4090\|3707-3738 | 32 | imported |
| 1297 | Gene.NFKB1\|NM_003998\|4093\|749-715 | 35 | imported |
| 1298 | Gene.NFKB1\|NM_003998\|4093\|715-749 | 35 | imported |
| 1299 | Gene.NFKB1\|NM_003998\|4093\|2186-2143 | 44 | imported |
| 1300 | Gene.NFKB1\|NM_003998\|4093\|2143-2186 | 44 | imported |
| 1301 | Gene.NFKB2\|NM_001077494\|3125\|684-651 | 34 | imported |
| 1302 | Gene.NFKB2\|NM_001077494\|3125\|651-684 | 34 | imported |
| 1303 | Gene.NFKB2\|NM_001077494\|3125\|1806-1769 | 38 | imported |
| 1304 | Gene.NFKB2\|NM_001077494\|3125\|1769-1806 | 38 | imported |
| 1305 | Gene.NFKB2\|NM_001077494\|3125\|1848-1821 | 28 | imported |
| 1306 | Gene.NFKB2\|NM_001077494\|3125\|1821-1848 | 28 | imported |
| 1307 | Gene.NHEJ1\|NM_024782\|2119\|1061-1027 | 35 | imported |
| 1308 | Gene.NHEJ1\|NM_024782\|2119\|1027-1061 | 35 | imported |
| 1309 | Gene.NHEJ1\|NM_024782\|2119\|1477-1441 | 37 | imported |
| 1310 | Gene.NHEJ1\|NM_024782\|2119\|1441-1477 | 37 | imported |
| 1311 | Gene.NHEJ1\|NM_024782\|2119\|2058-2017 | 42 | imported |
| 1312 | Gene.NHEJ1\|NM_024782\|2119\|2017-2058 | 42 | imported |
| 1313 | Gene.NLRP2\|NM_001174081\|3560\|2518-2491 | 28 | imported |
| 1314 | Gene.NLRP2\|NM_001174081\|3560\|2491-2518 | 28 | imported |
| 1315 | Gene.NLRP2\|NM_001174082\|3524\|607-581 | 27 | imported |
| 1316 | Gene.NLRP2\|NM_001174082\|3524\|581-607 | 27 | imported |
| 1317 | Gene.NLRP2\|NM_001174082\|3524\|2466-2437 | 30 | imported |
| 1318 | Gene.NLRP2\|NM_001174082\|3524\|2437-2466 | 30 | imported |
| 1319 | Gene.NOS3\|NM_000603\|4345\|965-937 | 29 | imported |
| 1320 | Gene.NOS3\|NM_000603\|4345\|937-965 | 29 | imported |
| 1321 | Gene.NOS3\|NM_001160109\|2040\|27-1 | 27 | imported |
| 1322 | Gene.NOS3\|NM_001160109\|2040\|1-27 | 27 | imported |
| 1323 | Gene.NOS3\|NM_001160109\|2040\|1498-1463 | 36 | imported |
| 1324 | Gene.NOS3\|NM_001160109\|2040\|1463-1498 | 36 | imported |
| 1325 | Gene.NOTCH1\|NM_017617\|9322\|1824-1795 | 30 | imported |
| 1326 | Gene.NOTCH1\|NM_017617\|9322\|1795-1824 | 30 | imported |
| 1327 | Gene.NOTCH1\|NM_017617\|9322\|4786-4759 | 28 | imported |
| 1328 | Gene.NOTCH1\|NM_017617\|9322\|4759-4786 | 28 | imported |
| 1329 | Gene.NOTCH1\|NM_017617\|9322\|5176-5149 | 28 | imported |
| 1330 | Gene.NOTCH1\|NM_017617\|9322\|5149-5176 | 28 | imported |
| 1331 | Gene.NOTCH4\|NM_004557\|6762\|1315-1289 | 27 | imported |
| 1332 | Gene.NOTCH4\|NM_004557\|6762\|1289-1315 | 27 | imported |
| 1333 | Gene.NOTCH4\|NM_004557\|6762\|3441-3417 | 25 | imported |
| 1334 | Gene.NOTCH4\|NM_004557\|6762\|3417-3441 | 25 | imported |
| 1335 | Gene.NOTCH4\|NM_004557\|6762\|5236-5209 | 28 | imported |
| 1336 | Gene.NOTCH4\|NM_004557\|6762\|5209-5236 | 28 | imported |
| 1337 | Gene.NRP1\|NM_001024628\|2478\|1537-1513 | 25 | imported |
| 1338 | Gene.NRP1\|NM_001024628\|2478\|1513-1537 | 25 | imported |
| 1339 | Gene.NRP1\|NM_001024628\|2478\|1756-1723 | 34 | imported |
| 1340 | Gene.NRP1\|NM_001024628\|2478\|1723-1756 | 34 | imported |
| 1341 | Gene.NRP1\|NM_001024628\|2478\|2194-2164 | 31 | imported |
| 1342 | Gene.NRP1\|NM_001024628\|2478\|2164-2194 | 31 | imported |
| 1343 | Gene.NRP2\|NM_003872\|6656\|491-449 | 43 | imported |
| 1344 | Gene.NRP2\|NM_003872\|6656\|449-491 | 43 | imported |
| 1345 | Gene.NRP2\|NM_018534\|4163\|2341-2311 | 31 | imported |
| 1346 | Gene.NRP2\|NM_018534\|4163\|2311-2341 | 31 | imported |
| 1347 | Gene.NRP2\|NM_201264\|2601\|2361-2333 | 29 | imported |
| 1348 | Gene.NRP2\|NM_201264\|2601\|2333-2361 | 29 | imported |
| 1349 | Gene.OGT\|NM_181672\|5497\|816-783 | 34 | imported |
| 1350 | Gene.OGT\|NM_181672\|5497\|783-816 | 34 | imported |
| 1351 | Gene.OGT\|NM_181672\|5497\|2704-2669 | 36 | imported |
| 1352 | Gene.OGT\|NM_181672\|5497\|2669-2704 | 36 | imported |
| 1353 | Gene.OGT\|NM_181673\|5467\|1874-1841 | 34 | imported |
| 1354 | Gene.OGT\|NM_181673\|5467\|1841-1874 | 34 | imported |
| 1355 | Gene.ORAOV1\|NM_153451\|2544\|657-631 | 27 | imported |
| 1356 | Gene.ORAOV1\|NM_153451\|2544\|631-657 | 27 | imported |
| 1357 | Gene.ORAOV1\|NM_153451\|2544\|762-736 | 27 | imported |
| 1358 | Gene.ORAOV1\|NM_153451\|2544\|736-762 | 27 | imported |
| 1359 | Gene.ORAOV1\|NM_153451\|2544\|804-778 | 27 | imported |
| 1360 | Gene.ORAOV1\|NM_153451\|2544\|778-804 | 27 | imported |
| 1361 | Gene.PARP1\|NM_001618\|4001\|2008-1981 | 28 | imported |
| 1362 | Gene.PARP1\|NM_001618\|4001\|1981-2008 | 28 | imported |
| 1363 | Gene.PARP1\|NM_001618\|4001\|2741-2707 | 35 | imported |
| 1364 | Gene.PARP1\|NM_001618\|4001\|2707-2741 | 35 | imported |
| 1365 | Gene.PARP1\|NM_001618\|4001\|2803-2773 | 31 | imported |
| 1366 | Gene.PARP1\|NM_001618\|4001\|2773-2803 | 31 | imported |
| 1367 | Gene.PC\|NM_001040716\|4192\|4015-3991 | 25 | imported |
| 1368 | Gene.PC\|NM_001040716\|4192\|3991-4015 | 25 | imported |
| 1369 | Gene.PC\|NM_022172\|3959\|289-265 | 25 | imported |
| 1370 | Gene.PC\|NM_022172\|3959\|265-289 | 25 | imported |
| 1371 | Gene.PC\|NM_022172\|3959\|1283-1255 | 29 | imported |
| 1372 | Gene.PC\|NM_022172\|3959\|1255-1283 | 29 | imported |
| 1373 | Gene.PDGFA\|NM_002607\|2809\|1575-1537 | 39 | imported |
| 1374 | Gene.PDGFA\|NM_002607\|2809\|1537-1575 | 39 | imported |
| 1375 | Gene.PDGFA\|NM_002607\|2809\|1831-1801 | 31 | imported |
| 1376 | Gene.PDGFA\|NM_002607\|2809\|1801-1831 | 31 | imported |
| 1377 | Gene.PDGFA\|NM_033023\|2740\|1823-1795 | 29 | imported |
| 1378 | Gene.PDGFA\|NM_033023\|2740\|1795-1823 | 29 | imported |
| 1379 | Gene.PDGFRA\|NM_006206\|6574\|1082-1046 | 37 | imported |
| 1380 | Gene.PDGFRA\|NM_006206\|6574\|1046-1082 | 37 | imported |
| 1381 | Gene.PDGFRA\|NM_006206\|6574\|5981-5941 | 41 | imported |
| 1382 | Gene.PDGFRA\|NM_006206\|6574\|5941-5981 | 41 | imported |
| 1383 | Gene.PDGFRA\|NM_006206\|6574\|6361-6326 | 36 | imported |
| 1384 | Gene.PDGFRA\|NM_006206\|6574\|6326-6361 | 36 | imported |
| 1385 | Gene.PDGFRB\|NM_002609\|5718\|2534-2497 | 38 | imported |
| 1386 | Gene.PDGFRB\|NM_002609\|5718\|2497-2534 | 38 | imported |
| 1387 | Gene.PDGFRB\|NM_002609\|5718\|3055-3025 | 31 | imported |
| 1388 | Gene.PDGFRB\|NM_002609\|5718\|3025-3055 | 31 | imported |
| 1389 | Gene.PDGFRB\|NM_002609\|5718\|3539-3505 | 35 | imported |
| 1390 | Gene.PDGFRB\|NM_002609\|5718\|3505-3539 | 35 | imported |
| 1391 | Gene.PDL1\|NM_001267706\|3349\|545-505 | 41 | imported |
| 1392 | Gene.PDL1\|NM_001267706\|3349\|505-545 | 41 | imported |
| 1393 | Gene.PDL1\|NM_001267706\|3349\|932-897 | 36 | imported |
| 1394 | Gene.PDL1\|NM_001267706\|3349\|897-932 | 36 | imported |
| 1395 | Gene.PDL1\|NM_001267706\|3349\|1575-1541 | 35 | imported |
| 1396 | Gene.PDL1\|NM_001267706\|3349\|1541-1575 | 35 | imported |
| 1397 | Gene.PGF\|NM_001207012\|1848\|938-913 | 26 | imported |
| 1398 | Gene.PGF\|NM_001207012\|1848\|913-938 | 26 | imported |
| 1399 | Gene.PGF\|NM_001207012\|1848\|217-193 | 25 | imported |
| 1400 | Gene.PGF\|NM_001207012\|1848\|193-217 | 25 | imported |
| 1401 | Gene.PGF\|NM_001207012\|1848\|382-353 | 30 | imported |
| 1402 | Gene.PGF\|NM_001207012\|1848\|353-382 | 30 | imported |
| 1403 | Gene.PHGDH\|NM_006623\|2021\|488-460 | 29 | imported |
| 1404 | Gene.PHGDH\|NM_006623\|2021\|460-488 | 29 | imported |
| 1405 | Gene.PHGDH\|NM_006623\|2021\|503-477 | 27 | imported |
| 1406 | Gene.PHGDH\|NM_006623\|2021\|477-503 | 27 | imported |
| 1407 | Gene.PHGDH\|NM_006623\|2021\|876-851 | 26 | imported |
| 1408 | Gene.PHGDH\|NM_006623\|2021\|851-876 | 26 | imported |
| 1409 | Gene.PIK3CA\|NM_006218\|9104\|185-153 | 33 | imported |
| 1410 | Gene.PIK3CA\|NM_006218\|9104\|153-185 | 33 | imported |
| 1411 | Gene.PIK3CA\|NM_006218\|9104\|1401-1369 | 33 | imported |
| 1412 | Gene.PIK3CA\|NM_006218\|9104\|1369-1401 | 33 | imported |
| 1413 | Gene.PIK3CA\|NM_006218\|9104\|1489-1445 | 45 | imported |
| 1414 | Gene.PIK3CA\|NM_006218\|9104\|1445-1489 | 45 | imported |
| 1415 | Gene.PINK1\|NM_032409\|2680\|645-617 | 29 | imported |
| 1416 | Gene.PINK1\|NM_032409\|2680\|617-645 | 29 | imported |
| 1417 | Gene.PINK1\|NM_032409\|2680\|710-683 | 28 | imported |
| 1418 | Gene.PINK1\|NM_032409\|2680\|683-710 | 28 | imported |
| 1419 | Gene.PINK1\|NM_032409\|2680\|781-749 | 33 | imported |
| 1420 | Gene.PINK1\|NM_032409\|2680\|749-781 | 33 | imported |
| 1421 | Gene.PLAU\|NM_001145031\|2683\|979-947 | 33 | imported |
| 1422 | Gene.PLAU\|NM_001145031\|2683\|947-979 | 33 | imported |
| 1423 | Gene.PLAU\|NM_001145031\|2683\|1286-1255 | 32 | imported |
| 1424 | Gene.PLAU\|NM_001145031\|2683\|1255-1286 | 32 | imported |
| 1425 | Gene.PLAU\|NM_002658\|2398\|1555-1521 | 35 | imported |
| 1426 | Gene.PLAU\|NM_002658\|2398\|1521-1555 | 35 | imported |
| 1427 | Gene.PLAUR\|NM_001005376\|1455\|583-553 | 31 | imported |
| 1428 | Gene.PLAUR\|NM_001005376\|1455\|553-583 | 31 | imported |
| 1429 | Gene.PLAUR\|NM_002659\|1570\|390-365 | 26 | imported |
| 1430 | Gene.PLAUR\|NM_002659\|1570\|365-390 | 26 | imported |
| 1431 | Gene.PLAUR\|NM_002659\|1570\|615-586 | 30 | imported |
| 1432 | Gene.PLAUR\|NM_002659\|1570\|586-615 | 30 | imported |
| 1433 | Gene.PLD1\|NM_001130081\|5911\|2975-2941 | 35 | imported |
| 1434 | Gene.PLD1\|NM_001130081\|5911\|2941-2975 | 35 | imported |
| 1435 | Gene.PLD1\|NM_001130081\|5911\|4641-4607 | 35 | imported |
| 1436 | Gene.PLD1\|NM_001130081\|5911\|4607-4641 | 35 | imported |
| 1437 | Gene.PLD1\|NM_001130081\|5911\|4798-4754 | 45 | imported |
| 1438 | Gene.PLD1\|NM_001130081\|5911\|4754-4798 | 45 | imported |
| 1439 | Gene.PMAIP1\|NM_021127\|1954\|27-1 | 27 | imported |
| 1440 | Gene.PMAIP1\|NM_021127\|1954\|1-27 | 27 | imported |
| 1441 | Gene.PMAIP1\|NM_021127\|1954\|737-705 | 33 | imported |
| 1442 | Gene.PMAIP1\|NM_021127\|1954\|705-737 | 33 | imported |
| 1443 | Gene.PMAIP1\|NM_021127\|1954\|779-753 | 27 | imported |
| 1444 | Gene.PMAIP1\|NM_021127\|1954\|753-779 | 27 | imported |
| 1445 | Gene.POSTN\|NM_001135934\|3219\|1600-1567 | 34 | imported |
| 1446 | Gene.POSTN\|NM_001135934\|3219\|1567-1600 | 34 | imported |
| 1447 | Gene.POSTN\|NM_001135935\|3225\|2551-2512 | 40 | imported |
| 1448 | Gene.POSTN\|NM_001135935\|3225\|2512-2551 | 40 | imported |
| 1449 | Gene.POSTN\|NM_006475\|3390\|822-785 | 38 | imported |
| 1450 | Gene.POSTN\|NM_006475\|3390\|785-822 | 38 | imported |
| 1451 | Gene.POU5F1\|NM_002701\|1430\|1234-1201 | 34 | imported |
| 1452 | Gene.POU5F1\|NM_002701\|1430\|1201-1234 | 34 | imported |
| 1453 | Gene.POU5F1\|NM_001173531\|1589\|1578-1535 | 44 | imported |
| 1454 | Gene.POU5F1\|NM_001173531\|1589\|1535-1578 | 44 | imported |
| 1455 | Gene.POU5F1\|NM_203289\|2075\|1830-1803 | 28 | imported |
| 1456 | Gene.POU5F1\|NM_203289\|2075\|1803-1830 | 28 | imported |
| 1457 | Gene.PRKCA\|NM_002737\|8787\|1124-1096 | 29 | imported |
| 1458 | Gene.PRKCA\|NM_002737\|8787\|1096-1124 | 29 | imported |
| 1459 | Gene.PRKCA\|NM_002737\|8787\|3970-3943 | 28 | imported |
| 1460 | Gene.PRKCA\|NM_002737\|8787\|3943-3970 | 28 | imported |
| 1461 | Gene.PRKCA\|NM_002737\|8787\|4845-4819 | 27 | imported |
| 1462 | Gene.PRKCA\|NM_002737\|8787\|4819-4845 | 27 | imported |
| 1463 | Gene.PRKDC\|NM_001081640\|13416\|4067-4033 | 35 | imported |
| 1464 | Gene.PRKDC\|NM_001081640\|13416\|4033-4067 | 35 | imported |
| 1465 | Gene.PRKDC\|NM_006904\|13509\|2868-2826 | 43 | imported |
| 1466 | Gene.PRKDC\|NM_006904\|13509\|2826-2868 | 43 | imported |
| 1467 | Gene.PRKDC\|NM_006904\|13509\|4553-4521 | 33 | imported |
| 1468 | Gene.PRKDC\|NM_006904\|13509\|4521-4553 | 33 | imported |
| 1469 | Gene.PRODH\|XM_017030168\|2048\|689-664 | 26 | imported |
| 1470 | Gene.PRODH\|XM_017030168\|2048\|664-689 | 26 | imported |
| 1471 | Gene.PRODH\|XM_006724935\|2230\|639-609 | 31 | imported |
| 1472 | Gene.PRODH\|XM_006724935\|2230\|609-639 | 31 | imported |
| 1473 | Gene.PRODH\|XM_006724935\|2230\|921-894 | 28 | imported |
| 1474 | Gene.PRODH\|XM_006724935\|2230\|894-921 | 28 | imported |
| 1475 | Gene.PROM1\|NM_001145847\|4257\|1329-1297 | 33 | imported |
| 1476 | Gene.PROM1\|NM_001145847\|4257\|1297-1329 | 33 | imported |
| 1477 | Gene.PROM1\|NM_001145848\|3973\|1218-1189 | 30 | imported |
| 1478 | Gene.PROM1\|NM_001145848\|3973\|1189-1218 | 30 | imported |
| 1479 | Gene.PROM1\|NM_001145848\|3973\|1332-1288 | 45 | imported |
| 1480 | Gene.PROM1\|NM_001145848\|3973\|1288-1332 | 45 | imported |
| 1481 | Gene.PSAT1\|NM_021154\|2132\|1068-1027 | 42 | imported |
| 1482 | Gene.PSAT1\|NM_021154\|2132\|1027-1068 | 42 | imported |
| 1483 | Gene.PSAT1\|NM_021154\|2132\|1565-1531 | 35 | imported |
| 1484 | Gene.PSAT1\|NM_021154\|2132\|1531-1565 | 35 | imported |
| 1485 | Gene.PSAT1\|NM_058179\|2270\|1727-1692 | 36 | imported |
| 1486 | Gene.PSAT1\|NM_058179\|2270\|1692-1727 | 36 | imported |
| 1487 | Gene.PSMB2\|NM_002794\|4759\|713-681 | 33 | imported |
| 1488 | Gene.PSMB2\|NM_002794\|4759\|681-713 | 33 | imported |
| 1489 | Gene.PSMB2\|NM_002794\|4759\|1307-1281 | 27 | imported |
| 1490 | Gene.PSMB2\|NM_002794\|4759\|1281-1307 | 27 | imported |
| 1491 | Gene.PSMB2\|NM_002794\|4759\|3549-3521 | 29 | imported |
| 1492 | Gene.PSMB2\|NM_002794\|4759\|3521-3549 | 29 | imported |
| 1493 | Gene.PSMD14\|NM_005805\|1734\|1380-1336 | 45 | imported |
| 1494 | Gene.PSMD14\|NM_005805\|1734\|1336-1380 | 45 | imported |
| 1495 | Gene.PSMD14\|NM_005805\|1734\|1509-1471 | 39 | imported |
| 1496 | Gene.PSMD14\|NM_005805\|1734\|1471-1509 | 39 | imported |
| 1497 | Gene.PSMD14\|NM_005805\|1734\|1620-1576 | 45 | imported |
| 1498 | Gene.PSMD14\|NM_005805\|1734\|1576-1620 | 45 | imported |
| 1499 | Gene.PTEN\|NM_000314\|8718\|2739-2702 | 38 | imported |
| 1500 | Gene.PTEN\|NM_000314\|8718\|2702-2739 | 38 | imported |
| 1501 | Gene.PTEN\|NM_001304718\|8833\|5144-5107 | 38 | imported |
| 1502 | Gene.PTEN\|NM_001304718\|8833\|5107-5144 | 38 | imported |
| 1503 | Gene.PTEN\|NM_000314\|8718\|249-220 | 30 | imported |
| 1504 | Gene.PTEN\|NM_000314\|8718\|220-249 | 30 | imported |
| 1505 | Gene.PTGS1\|NM_000962\|5045\|1797-1765 | 33 | imported |
| 1506 | Gene.PTGS1\|NM_000962\|5045\|1765-1797 | 33 | imported |
| 1507 | Gene.PTGS1\|NM_001271166\|5160\|4249-4215 | 35 | imported |
| 1508 | Gene.PTGS1\|NM_001271166\|5160\|4215-4249 | 35 | imported |
| 1509 | Gene.PTGS1\|NM_001271368\|5382\|3410-3376 | 35 | imported |
| 1510 | Gene.PTGS1\|NM_001271368\|5382\|3376-3410 | 35 | imported |
| 1511 | Gene.PTGS2\|NM_000963\|4507\|756-723 | 34 | imported |
| 1512 | Gene.PTGS2\|NM_000963\|4507\|723-756 | 34 | imported |
| 1513 | Gene.PTGS2\|NM_000963\|4507\|830-799 | 32 | imported |
| 1514 | Gene.PTGS2\|NM_000963\|4507\|799-830 | 32 | imported |
| 1515 | Gene.PTGS2\|NM_000963\|4507\|1398-1369 | 30 | imported |
| 1516 | Gene.PTGS2\|NM_000963\|4507\|1369-1398 | 30 | imported |
| 1517 | Gene.PYCR1\|NM_001282279\|1811\|451-421 | 31 | imported |
| 1518 | Gene.PYCR1\|NM_001282279\|1811\|421-451 | 31 | imported |
| 1519 | Gene.PYCR1\|NM_001282279\|1811\|535-511 | 25 | imported |
| 1520 | Gene.PYCR1\|NM_001282279\|1811\|511-535 | 25 | imported |
| 1521 | Gene.PYCR1\|NM_006907\|2233\|1870-1844 | 27 | imported |
| 1522 | Gene.PYCR1\|NM_006907\|2233\|1844-1870 | 27 | imported |
| 1523 | Gene.RAC1\|NM_006908\|2341\|70-41 | 30 | imported |
| 1524 | Gene.RAC1\|NM_006908\|2341\|41-70 | 30 | imported |
| 1525 | Gene.RAC1\|NM_006908\|2341\|205-181 | 25 | imported |
| 1526 | Gene.RAC1\|NM_006908\|2341\|181-205 | 25 | imported |
| 1527 | Gene.RAC1\|NM_006908\|2341\|1332-1301 | 32 | imported |
| 1528 | Gene.RAC1\|NM_006908\|2341\|1301-1332 | 32 | imported |
| 1529 | Gene.RAC2\|NM_002872\|1538\|103-79 | 25 | imported |
| 1530 | Gene.RAC2\|NM_002872\|1538\|79-103 | 25 | imported |
| 1531 | Gene.RAC2\|NM_002872\|1538\|144-118 | 27 | imported |
| 1532 | Gene.RAC2\|NM_002872\|1538\|118-144 | 27 | imported |
| 1533 | Gene.RAC2\|NM_002872\|1538\|238-209 | 30 | imported |
| 1534 | Gene.RAC2\|NM_002872\|1538\|209-238 | 30 | imported |
| 1535 | Gene.RAD1\|NM_002853\|4683\|435-391 | 45 | imported |
| 1536 | Gene.RAD1\|NM_002853\|4683\|391-435 | 45 | imported |
| 1537 | Gene.RAD1\|NM_002853\|4683\|825-781 | 45 | imported |
| 1538 | Gene.RAD1\|NM_002853\|4683\|781-825 | 45 | imported |
| 1539 | Gene.RAD1\|NM_002853\|4683\|902-859 | 44 | imported |
| 1540 | Gene.RAD1\|NM_002853\|4683\|859-902 | 44 | imported |
| 1541 | Gene.RAD50\|NM_005732\|6597\|3063-3026 | 38 | imported |
| 1542 | Gene.RAD50\|NM_005732\|6597\|3026-3063 | 38 | imported |
| 1543 | Gene.RAD50\|NM_005732\|6597\|3993-3961 | 33 | imported |
| 1544 | Gene.RAD50\|NM_005732\|6597\|3961-3993 | 33 | imported |
| 1545 | Gene.RAD50\|NM_005732\|6597\|5151-5116 | 36 | imported |
| 1546 | Gene.RAD50\|NM_005732\|6597\|5116-5151 | 36 | imported |
| 1547 | Gene.RAD51\|NM_001164269\|2147\|1318-1279 | 40 | imported |
| 1548 | Gene.RAD51\|NM_001164269\|2147\|1279-1318 | 40 | imported |
| 1549 | Gene.RAD51\|NM_001164269\|2147\|1800-1765 | 36 | imported |
| 1550 | Gene.RAD51\|NM_001164269\|2147\|1765-1800 | 36 | imported |
| 1551 | Gene.RAD51\|NM_002875\|2299\|1073-1046 | 28 | imported |
| 1552 | Gene.RAD51\|NM_002875\|2299\|1046-1073 | 28 | imported |
| 1553 | Gene.RAD51C\|NM_002876\|666\|111-81 | 31 | imported |
| 1554 | Gene.RAD51C\|NM_002876\|666\|81-111 | 31 | imported |
| 1555 | Gene.RAD51C\|NM_002876\|666\|138-111 | 28 | imported |
| 1556 | Gene.RAD51C\|NM_002876\|666\|111-138 | 28 | imported |
| 1557 | Gene.RAD51C\|NM_002876\|666\|176-151 | 26 | imported |
| 1558 | Gene.RAD51C\|NM_002876\|666\|151-176 | 26 | imported |
| 1559 | Gene.RAD52\|NM_134424\|3051\|454-417 | 38 | imported |
| 1560 | Gene.RAD52\|NM_134424\|3051\|417-454 | 38 | imported |
| 1561 | Gene.RAD52\|NM_134424\|3051\|1493-1457 | 37 | imported |
| 1562 | Gene.RAD52\|NM_134424\|3051\|1457-1493 | 37 | imported |
| 1563 | Gene.RAD52\|NM_134424\|3051\|1598-1561 | 38 | imported |
| 1564 | Gene.RAD52\|NM_134424\|3051\|1561-1598 | 38 | imported |
| 1565 | Gene.RAD54L\|NM_001142548\|2567\|208-177 | 32 | imported |
| 1566 | Gene.RAD54L\|NM_001142548\|2567\|177-208 | 32 | imported |
| 1567 | Gene.RAD54L\|NM_001142548\|2567\|1237-1211 | 27 | imported |
| 1568 | Gene.RAD54L\|NM_001142548\|2567\|1211-1237 | 27 | imported |
| 1569 | Gene.RAD54L\|NM_001142548\|2567\|1899-1871 | 29 | imported |
| 1570 | Gene.RAD54L\|NM_001142548\|2567\|1871-1899 | 29 | imported |
| 1571 | Gene.RAD9A\|NM_001243224\|1992\|1560-1531 | 30 | imported |
| 1572 | Gene.RAD9A\|NM_001243224\|1992\|1531-1560 | 30 | imported |
| 1573 | Gene.RAD9A\|NM_001243224\|1992\|1648-1616 | 33 | imported |
| 1574 | Gene.RAD9A\|NM_001243224\|1992\|1616-1648 | 33 | imported |
| 1575 | Gene.RAD9A\|NM_001243224\|1992\|1727-1701 | 27 | imported |
| 1576 | Gene.RAD9A\|NM_001243224\|1992\|1701-1727 | 27 | imported |
| 1577 | Gene.RAG1\|NM_000448\|6582\|3330-3301 | 30 | imported |
| 1578 | Gene.RAG1\|NM_000448\|6582\|3301-3330 | 30 | imported |
| 1579 | Gene.RAG1\|NM_000448\|6582\|5489-5446 | 44 | imported |
| 1580 | Gene.RAG1\|NM_000448\|6582\|5446-5489 | 44 | imported |
| 1581 | Gene.RAG1\|NM_000448\|6582\|5761-5721 | 41 | imported |
| 1582 | Gene.RAG1\|NM_000448\|6582\|5721-5761 | 41 | imported |
| 1583 | Gene.RB1\|NM_000321\|4772\|2194-2161 | 34 | imported |
| 1584 | Gene.RB1\|NM_000321\|4772\|2161-2194 | 34 | imported |
| 1585 | Gene.RB1\|NM_000321\|4772\|2231-2201 | 31 | imported |
| 1586 | Gene.RB1\|NM_000321\|4772\|2201-2231 | 31 | imported |
| 1587 | Gene.RB1\|NM_000321\|4772\|2485-2441 | 45 | imported |
| 1588 | Gene.RB1\|NM_000321\|4772\|2441-2485 | 45 | imported |
| 1589 | Gene.RBBP8\|NM_002894\|3279\|931-892 | 40 | imported |
| 1590 | Gene.RBBP8\|NM_002894\|3279\|892-931 | 40 | imported |
| 1591 | Gene.RBBP8\|NM_002894\|3279\|1656-1621 | 36 | imported |
| 1592 | Gene.RBBP8\|NM_002894\|3279\|1621-1656 | 36 | imported |
| 1593 | Gene.RBBP8\|NM_002894\|3279\|1974-1945 | 30 | imported |
| 1594 | Gene.RBBP8\|NM_002894\|3279\|1945-1974 | 30 | imported |
| 1595 | Gene.REC8\|NM_001048205\|2354\|586-561 | 26 | imported |
| 1596 | Gene.REC8\|NM_001048205\|2354\|561-586 | 26 | imported |
| 1597 | Gene.REC8\|NM_001048205\|2354\|2198-2161 | 38 | imported |
| 1598 | Gene.REC8\|NM_001048205\|2354\|2161-2198 | 38 | imported |
| 1599 | Gene.REC8\|NM_005132\|2253\|1150-1122 | 29 | imported |
| 1600 | Gene.REC8\|NM_005132\|2253\|1122-1150 | 29 | imported |
| 1601 | Gene.RECQL4\|NM_004260\|3840\|448-417 | 32 | imported |
| 1602 | Gene.RECQL4\|NM_004260\|3840\|417-448 | 32 | imported |
| 1603 | Gene.RECQL4\|NM_004260\|3840\|1726-1697 | 30 | imported |
| 1604 | Gene.RECQL4\|NM_004260\|3840\|1697-1726 | 30 | imported |
| 1605 | Gene.RECQL4\|NM_004260\|3840\|3739-3713 | 27 | imported |
| 1606 | Gene.RECQL4\|NM_004260\|3840\|3713-3739 | 27 | imported |
| 1607 | Gene.REEP5\|NM_005669\|3116\|548-521 | 28 | imported |
| 1608 | Gene.REEP5\|NM_005669\|3116\|521-548 | 28 | imported |
| 1609 | Gene.REEP5\|NM_005669\|3116\|1991-1951 | 41 | imported |
| 1610 | Gene.REEP5\|NM_005669\|3116\|1951-1991 | 41 | imported |
| 1611 | Gene.REEP5\|NM_005669\|3116\|2619-2575 | 45 | imported |
| 1612 | Gene.REEP5\|NM_005669\|3116\|2575-2619 | 45 | imported |
| 1613 | Gene.RELA\|NM_021975\|2595\|514-485 | 30 | imported |
| 1614 | Gene.RELA\|NM_021975\|2595\|485-514 | 30 | imported |
| 1615 | Gene.RELA\|NM_021975\|2595\|807-771 | 37 | imported |
| 1616 | Gene.RELA\|NM_021975\|2595\|771-807 | 37 | imported |
| 1617 | Gene.RELA\|NM_021975\|2595\|1524-1497 | 28 | imported |
| 1618 | Gene.RELA\|NM_021975\|2595\|1497-1524 | 28 | imported |
| 1619 | Gene.RHOA\|NM_001664\|1943\|385-353 | 33 | imported |
| 1620 | Gene.RHOA\|NM_001664\|1943\|353-385 | 33 | imported |
| 1621 | Gene.RHOA\|NM_001664\|1943\|539-497 | 43 | imported |
| 1622 | Gene.RHOA\|NM_001664\|1943\|497-539 | 43 | imported |
| 1623 | Gene.RHOA\|NM_001664\|1943\|1735-1697 | 39 | imported |
| 1624 | Gene.RHOA\|NM_001664\|1943\|1697-1735 | 39 | imported |
| 1625 | Gene.RHOB\|NM_004040\|2387\|707-681 | 27 | imported |
| 1626 | Gene.RHOB\|NM_004040\|2387\|681-707 | 27 | imported |
| 1627 | Gene.RHOB\|NM_004040\|2387\|1225-1201 | 25 | imported |
| 1628 | Gene.RHOB\|NM_004040\|2387\|1201-1225 | 25 | imported |
| 1629 | Gene.RHOB\|NM_004040\|2387\|1271-1241 | 31 | imported |
| 1630 | Gene.RHOB\|NM_004040\|2387\|1241-1271 | 31 | imported |
| 1631 | Gene.RHOC\|NM_001042678\|1346\|896-870 | 27 | imported |
| 1632 | Gene.RHOC\|NM_001042678\|1346\|870-896 | 27 | imported |
| 1633 | Gene.RHOC\|NM_001042678\|1346\|1240-1211 | 30 | imported |
| 1634 | Gene.RHOC\|NM_001042678\|1346\|1211-1240 | 30 | imported |
| 1635 | Gene.RHOC\|NM_001042678\|1346\|1283-1255 | 29 | imported |
| 1636 | Gene.RHOC\|NM_001042678\|1346\|1255-1283 | 29 | imported |
| 1637 | Gene.RNF168\|NM_152617\|5365\|391-361 | 31 | imported |
| 1638 | Gene.RNF168\|NM_152617\|5365\|361-391 | 31 | imported |
| 1639 | Gene.RNF168\|NM_152617\|5365\|935-901 | 35 | imported |
| 1640 | Gene.RNF168\|NM_152617\|5365\|901-935 | 35 | imported |
| 1641 | Gene.RNF168\|NM_152617\|5365\|1612-1576 | 37 | imported |
| 1642 | Gene.RNF168\|NM_152617\|5365\|1576-1612 | 37 | imported |
| 1643 | Gene.RNF8\|NM_003958\|5639\|977-941 | 37 | imported |
| 1644 | Gene.RNF8\|NM_003958\|5639\|941-977 | 37 | imported |
| 1645 | Gene.RNF8\|NM_003958\|5639\|4928-4889 | 40 | imported |
| 1646 | Gene.RNF8\|NM_003958\|5639\|4889-4928 | 40 | imported |
| 1647 | Gene.RNF8\|NM_183078\|5434\|5387-5356 | 32 | imported |
| 1648 | Gene.RNF8\|NM_183078\|5434\|5356-5387 | 32 | imported |
| 1649 | Gene.ROCK1\|NM_005406\|6650\|1986-1961 | 26 | imported |
| 1650 | Gene.ROCK1\|NM_005406\|6650\|1961-1986 | 26 | imported |
| 1651 | Gene.ROCK1\|NM_005406\|6650\|3516-3473 | 44 | imported |
| 1652 | Gene.ROCK1\|NM_005406\|6650\|3473-3516 | 44 | imported |
| 1653 | Gene.ROCK1\|NM_005406\|6650\|4181-4145 | 37 | imported |
| 1654 | Gene.ROCK1\|NM_005406\|6650\|4145-4181 | 37 | imported |
| 1655 | Gene.ROCK2\|NM_004850\|8310\|791-760 | 32 | imported |
| 1656 | Gene.ROCK2\|NM_004850\|8310\|760-791 | 32 | imported |
| 1657 | Gene.ROCK2\|NM_004850\|8310\|1350-1312 | 39 | imported |
| 1658 | Gene.ROCK2\|NM_004850\|8310\|1312-1350 | 39 | imported |
| 1659 | Gene.ROCK2\|NM_004850\|8310\|3072-3037 | 36 | imported |
| 1660 | Gene.ROCK2\|NM_004850\|8310\|3037-3072 | 36 | imported |
| 1661 | Gene.RPA1\|NM_002945\|4345\|366-325 | 42 | imported |
| 1662 | Gene.RPA1\|NM_002945\|4345\|325-366 | 42 | imported |
| 1663 | Gene.RPA1\|NM_002945\|4345\|678-649 | 30 | imported |
| 1664 | Gene.RPA1\|NM_002945\|4345\|649-678 | 30 | imported |
| 1665 | Gene.RPA1\|NM_002945\|4345\|904-865 | 40 | imported |
| 1666 | Gene.RPA1\|NM_002945\|4345\|865-904 | 40 | imported |
| 1667 | Gene.RPA2\|NM_002946\|1819\|800-766 | 35 | imported |
| 1668 | Gene.RPA2\|NM_002946\|1819\|766-800 | 35 | imported |
| 1669 | Gene.RPA2\|NM_002946\|1819\|834-796 | 39 | imported |
| 1670 | Gene.RPA2\|NM_002946\|1819\|796-834 | 39 | imported |
| 1671 | Gene.RPA2\|NM_002946\|1819\|1214-1171 | 44 | imported |
| 1672 | Gene.RPA2\|NM_002946\|1819\|1171-1214 | 44 | imported |
| 1673 | Gene.RPA3\|NM_002947\|1975\|1202-1174 | 29 | imported |
| 1674 | Gene.RPA3\|NM_002947\|1975\|1174-1202 | 29 | imported |
| 1675 | Gene.RPA3\|NM_002947\|1975\|1259-1225 | 35 | imported |
| 1676 | Gene.RPA3\|NM_002947\|1975\|1225-1259 | 35 | imported |
| 1677 | Gene.RPA3\|NM_002947\|1975\|1507-1463 | 45 | imported |
| 1678 | Gene.RPA3\|NM_002947\|1975\|1463-1507 | 45 | imported |
| 1679 | Gene.S100A4\|NM_019554\|564\|235-201 | 35 | imported |
| 1680 | Gene.S100A4\|NM_019554\|564\|201-235 | 35 | imported |
| 1681 | Gene.S100A4\|NM_002961\|512\|154-121 | 34 | imported |
| 1682 | Gene.S100A4\|NM_002961\|512\|121-154 | 34 | imported |
| 1683 | Gene.S100A4\|NM_002961\|512\|119-91 | 29 | imported |
| 1684 | Gene.S100A4\|NM_002961\|512\|91-119 | 29 | imported |
| 1685 | Gene.SDHA\|NM_001294332\|2659\|1346-1321 | 26 | imported |
| 1686 | Gene.SDHA\|NM_001294332\|2659\|1321-1346 | 26 | imported |
| 1687 | Gene.SDHA\|NM_001294332\|2659\|1744-1717 | 28 | imported |
| 1688 | Gene.SDHA\|NM_001294332\|2659\|1717-1744 | 28 | imported |
| 1689 | Gene.SDHA\|NM_001294332\|2659\|2139-2113 | 27 | imported |
| 1690 | Gene.SDHA\|NM_001294332\|2659\|2113-2139 | 27 | imported |
| 1691 | Gene.SERPINE1\|NM_000602\|3207\|28-1 | 28 | imported |
| 1692 | Gene.SERPINE1\|NM_000602\|3207\|1-28 | 28 | imported |
| 1693 | Gene.SERPINE1\|NM_000602\|3207\|1409-1378 | 32 | imported |
| 1694 | Gene.SERPINE1\|NM_000602\|3207\|1378-1409 | 32 | imported |
| 1695 | Gene.SERPINE1\|NM_000602\|3207\|1629-1594 | 36 | imported |
| 1696 | Gene.SERPINE1\|NM_000602\|3207\|1594-1629 | 36 | imported |
| 1697 | Gene.SHFM1\|NM_006304\|509\|321-281 | 41 | imported |
| 1698 | Gene.SHFM1\|NM_006304\|509\|281-321 | 41 | imported |
| 1699 | Gene.SHFM1\|NM_006304\|509\|404-361 | 44 | imported |
| 1700 | Gene.SHFM1\|NM_006304\|509\|361-404 | 44 | imported |
| 1701 | Gene.SHFM1\|NM_006304\|509\|423-391 | 33 | imported |
| 1702 | Gene.SHFM1\|NM_006304\|509\|391-423 | 33 | imported |
| 1703 | Gene.SIRT1\|NM_001142498\|3604\|369-331 | 39 | imported |
| 1704 | Gene.SIRT1\|NM_001142498\|3604\|331-369 | 39 | imported |
| 1705 | Gene.SIRT1\|NM_001142498\|3604\|395-361 | 35 | imported |
| 1706 | Gene.SIRT1\|NM_001142498\|3604\|361-395 | 35 | imported |
| 1707 | Gene.SIRT1\|NM_001142498\|3604\|750-721 | 30 | imported |
| 1708 | Gene.SIRT1\|NM_001142498\|3604\|721-750 | 30 | imported |
| 1709 | Gene.SIRT3\|NM_001017524\|2773\|27-1 | 27 | imported |
| 1710 | Gene.SIRT3\|NM_001017524\|2773\|1-27 | 27 | imported |
| 1711 | Gene.SIRT3\|NM_001017524\|2773\|516-484 | 33 | imported |
| 1712 | Gene.SIRT3\|NM_001017524\|2773\|484-516 | 33 | imported |
| 1713 | Gene.SIRT3\|NM_001017524\|2773\|855-829 | 27 | imported |
| 1714 | Gene.SIRT3\|NM_001017524\|2773\|829-855 | 27 | imported |
| 1715 | Gene.SIRT4\|NM_012240\|1213\|781-751 | 31 | imported |
| 1716 | Gene.SIRT4\|NM_012240\|1213\|751-781 | 31 | imported |
| 1717 | Gene.SIRT4\|NM_012240\|1213\|1048-1011 | 38 | imported |
| 1718 | Gene.SIRT4\|NM_012240\|1213\|1011-1048 | 38 | imported |
| 1719 | Gene.SIRT4\|NM_012240\|1213\|1117-1081 | 37 | imported |
| 1720 | Gene.SIRT4\|NM_012240\|1213\|1081-1117 | 37 | imported |
| 1721 | Gene.SIRT5\|NM_001193267\|4462\|855-815 | 41 | imported |
| 1722 | Gene.SIRT5\|NM_001193267\|4462\|815-855 | 41 | imported |
| 1723 | Gene.SIRT5\|NM_012241\|4538\|975-951 | 25 | imported |
| 1724 | Gene.SIRT5\|NM_012241\|4538\|951-975 | 25 | imported |
| 1725 | Gene.SIRT5\|NM_031244\|2426\|993-961 | 33 | imported |
| 1726 | Gene.SIRT5\|NM_031244\|2426\|961-993 | 33 | imported |
| 1727 | Gene.SLC16A1\|NM_001166496\|4390\|1284-1259 | 26 | imported |
| 1728 | Gene.SLC16A1\|NM_001166496\|4390\|1259-1284 | 26 | imported |
| 1729 | Gene.SLC16A1\|NM_001166496\|4390\|2291-2258 | 34 | imported |
| 1730 | Gene.SLC16A1\|NM_001166496\|4390\|2258-2291 | 34 | imported |
| 1731 | Gene.SLC16A1\|NM_001166496\|4390\|3372-3331 | 42 | imported |
| 1732 | Gene.SLC16A1\|NM_001166496\|4390\|3331-3372 | 42 | imported |
| 1733 | Gene.SLC16A4\|NM_001201546\|2485\|1250-1219 | 32 | imported |
| 1734 | Gene.SLC16A4\|NM_001201546\|2485\|1219-1250 | 32 | imported |
| 1735 | Gene.SLC16A4\|NM_001201546\|2485\|1274-1240 | 35 | imported |
| 1736 | Gene.SLC16A4\|NM_001201546\|2485\|1240-1274 | 35 | imported |
| 1737 | Gene.SLC16A4\|NM_001201546\|2485\|1317-1282 | 36 | imported |
| 1738 | Gene.SLC16A4\|NM_001201546\|2485\|1282-1317 | 36 | imported |
| 1739 | Gene.SLC1A5\|NM_001145144\|1737\|197-166 | 32 | imported |
| 1740 | Gene.SLC1A5\|NM_001145144\|1737\|166-197 | 32 | imported |
| 1741 | Gene.SLC1A5\|NM_001145144\|1737\|290-256 | 35 | imported |
| 1742 | Gene.SLC1A5\|NM_001145144\|1737\|256-290 | 35 | imported |
| 1743 | Gene.SLC1A5\|NM_001145145\|1927\|857-833 | 25 | imported |
| 1744 | Gene.SLC1A5\|NM_001145145\|1927\|833-857 | 25 | imported |
| 1745 | Gene.SLC2A1\|NM_006516\|3687\|305-280 | 26 | imported |
| 1746 | Gene.SLC2A1\|NM_006516\|3687\|280-305 | 26 | imported |
| 1747 | Gene.SLC2A1\|NM_006516\|3687\|1114-1086 | 29 | imported |
| 1748 | Gene.SLC2A1\|NM_006516\|3687\|1086-1114 | 29 | imported |
| 1749 | Gene.SLC2A1\|NM_006516\|3687\|1920-1892 | 29 | imported |
| 1750 | Gene.SLC2A1\|NM_006516\|3687\|1892-1920 | 29 | imported |
| 1751 | Gene.SLC30A1\|NM_021194\|2034\|1418-1378 | 41 | imported |
| 1752 | Gene.SLC30A1\|NM_021194\|2034\|1378-1418 | 41 | imported |
| 1753 | Gene.SLC30A1\|NM_021194\|2034\|1461-1429 | 33 | imported |
| 1754 | Gene.SLC30A1\|NM_021194\|2034\|1429-1461 | 33 | imported |
| 1755 | Gene.SLC30A1\|NM_021194\|2034\|1894-1854 | 41 | imported |
| 1756 | Gene.SLC30A1\|NM_021194\|2034\|1854-1894 | 41 | imported |
| 1757 | Gene.SLC30A4\|NM_013309\|7173\|992-961 | 32 | imported |
| 1758 | Gene.SLC30A4\|NM_013309\|7173\|961-992 | 32 | imported |
| 1759 | Gene.SLC30A4\|NM_013309\|7173\|1599-1561 | 39 | imported |
| 1760 | Gene.SLC30A4\|NM_013309\|7173\|1561-1599 | 39 | imported |
| 1761 | Gene.SLC30A4\|NM_013309\|7173\|1780-1741 | 40 | imported |
| 1762 | Gene.SLC30A4\|NM_013309\|7173\|1741-1780 | 40 | imported |
| 1763 | Gene.SLC30A5\|NM_022902\|4078\|587-545 | 43 | imported |
| 1764 | Gene.SLC30A5\|NM_022902\|4078\|545-587 | 43 | imported |
| 1765 | Gene.SLC30A5\|NM_024055\|1379\|74-49 | 26 | imported |
| 1766 | Gene.SLC30A5\|NM_024055\|1379\|49-74 | 26 | imported |
| 1767 | Gene.SLC30A5\|NM_024055\|1379\|85-61 | 25 | imported |
| 1768 | Gene.SLC30A5\|NM_024055\|1379\|61-85 | 25 | imported |
| 1769 | Gene.SLC30A7\|NM_001144884\|7918\|162-133 | 30 | imported |
| 1770 | Gene.SLC30A7\|NM_001144884\|7918\|133-162 | 30 | imported |
| 1771 | Gene.SLC30A7\|NM_001144884\|7918\|3343-3301 | 43 | imported |
| 1772 | Gene.SLC30A7\|NM_001144884\|7918\|3301-3343 | 43 | imported |
| 1773 | Gene.SLC30A7\|NM_133496\|8232\|1080-1036 | 45 | imported |
| 1774 | Gene.SLC30A7\|NM_133496\|8232\|1036-1080 | 45 | imported |
| 1775 | Gene.SLC30A9\|NM_006345\|3272\|52-28 | 25 | imported |
| 1776 | Gene.SLC30A9\|NM_006345\|3272\|28-52 | 25 | imported |
| 1777 | Gene.SLC30A9\|NM_006345\|3272\|1141-1108 | 34 | imported |
| 1778 | Gene.SLC30A9\|NM_006345\|3272\|1108-1141 | 34 | imported |
| 1779 | Gene.SLC30A9\|NM_006345\|3272\|1463-1432 | 32 | imported |
| 1780 | Gene.SLC30A9\|NM_006345\|3272\|1432-1463 | 32 | imported |
| 1781 | Gene.SLC31A1\|NM_001859\|4797\|795-761 | 35 | imported |
| 1782 | Gene.SLC31A1\|NM_001859\|4797\|761-795 | 35 | imported |
| 1783 | Gene.SLC31A1\|NM_001859\|4797\|2877-2841 | 37 | imported |
| 1784 | Gene.SLC31A1\|NM_001859\|4797\|2841-2877 | 37 | imported |
| 1785 | Gene.SLC31A1\|NM_001859\|4797\|3995-3961 | 35 | imported |
| 1786 | Gene.SLC31A1\|NM_001859\|4797\|3961-3995 | 35 | imported |
| 1787 | Gene.SLC38A5\|NM_033518\|1993\|320-290 | 31 | imported |
| 1788 | Gene.SLC38A5\|NM_033518\|1993\|290-320 | 31 | imported |
| 1789 | Gene.SLC38A5\|NM_033518\|1993\|868-834 | 35 | imported |
| 1790 | Gene.SLC38A5\|NM_033518\|1993\|834-868 | 35 | imported |
| 1791 | Gene.SLC38A5\|NM_033518\|1993\|1167-1140 | 28 | imported |
| 1792 | Gene.SLC38A5\|NM_033518\|1993\|1140-1167 | 28 | imported |
| 1793 | Gene.SLC39A1\|NM_001271957\|2318\|1967-1939 | 29 | imported |
| 1794 | Gene.SLC39A1\|NM_001271957\|2318\|1939-1967 | 29 | imported |
| 1795 | Gene.SLC39A1\|NM_001271958\|2155\|1503-1477 | 27 | imported |
| 1796 | Gene.SLC39A1\|NM_001271958\|2155\|1477-1503 | 27 | imported |
| 1797 | Gene.SLC39A1\|NM_001271959\|2324\|1267-1236 | 32 | imported |
| 1798 | Gene.SLC39A1\|NM_001271959\|2324\|1236-1267 | 32 | imported |
| 1799 | Gene.SLC39A2\|NM_001256588\|1413\|147-109 | 39 | imported |
| 1800 | Gene.SLC39A2\|NM_001256588\|1413\|109-147 | 39 | imported |
| 1801 | Gene.SLC39A2\|NM_001256588\|1413\|1002-973 | 30 | imported |
| 1802 | Gene.SLC39A2\|NM_001256588\|1413\|973-1002 | 30 | imported |
| 1803 | Gene.SLC39A2\|NM_001256588\|1413\|1137-1105 | 33 | imported |
| 1804 | Gene.SLC39A2\|NM_001256588\|1413\|1105-1137 | 33 | imported |
| 1805 | Gene.SLC39A3\|NM_144564\|1441\|206-181 | 26 | imported |
| 1806 | Gene.SLC39A3\|NM_144564\|1441\|181-206 | 26 | imported |
| 1807 | Gene.SLC39A3\|NM_144564\|1441\|232-205 | 28 | imported |
| 1808 | Gene.SLC39A3\|NM_144564\|1441\|205-232 | 28 | imported |
| 1809 | Gene.SLC39A3\|NM_144564\|1441\|384-349 | 36 | imported |
| 1810 | Gene.SLC39A3\|NM_144564\|1441\|349-384 | 36 | imported |
| 1811 | Gene.SLC39A4\|NM_001280557\|697\|125-101 | 25 | imported |
| 1812 | Gene.SLC39A4\|NM_001280557\|697\|101-125 | 25 | imported |
| 1813 | Gene.SLC39A4\|NM_001280557\|697\|407-381 | 27 | imported |
| 1814 | Gene.SLC39A4\|NM_001280557\|697\|381-407 | 27 | imported |
| 1815 | Gene.SLC39A4\|NM_001280557\|697\|448-421 | 28 | imported |
| 1816 | Gene.SLC39A4\|NM_001280557\|697\|421-448 | 28 | imported |
| 1817 | Gene.SLC39A6\|NM_001099406\|1681\|1014-981 | 34 | imported |
| 1818 | Gene.SLC39A6\|NM_001099406\|1681\|981-1014 | 34 | imported |
| 1819 | Gene.SLC39A6\|NM_001099406\|1681\|1091-1051 | 41 | imported |
| 1820 | Gene.SLC39A6\|NM_001099406\|1681\|1051-1091 | 41 | imported |
| 1821 | Gene.SLC39A6\|NM_001099406\|1681\|1346-1317 | 30 | imported |
| 1822 | Gene.SLC39A6\|NM_001099406\|1681\|1317-1346 | 30 | imported |
| 1823 | Gene.SLC39A7\|NM_001077516\|2172\|66-37 | 30 | imported |
| 1824 | Gene.SLC39A7\|NM_001077516\|2172\|37-66 | 30 | imported |
| 1825 | Gene.SLC39A7\|NM_001077516\|2172\|101-73 | 29 | imported |
| 1826 | Gene.SLC39A7\|NM_001077516\|2172\|73-101 | 29 | imported |
| 1827 | Gene.SLC39A7\|NM_001077516\|2172\|1630-1603 | 28 | imported |
| 1828 | Gene.SLC39A7\|NM_001077516\|2172\|1603-1630 | 28 | imported |
| 1829 | Gene.SLC39A8\|NM_001135146\|3187\|1172-1135 | 38 | imported |
| 1830 | Gene.SLC39A8\|NM_001135146\|3187\|1135-1172 | 38 | imported |
| 1831 | Gene.SLC39A8\|NM_001135146\|3187\|1302-1270 | 33 | imported |
| 1832 | Gene.SLC39A8\|NM_001135146\|3187\|1270-1302 | 33 | imported |
| 1833 | Gene.SLC39A8\|NM_001135148\|2624\|1050-1013 | 38 | imported |
| 1834 | Gene.SLC39A8\|NM_001135148\|2624\|1013-1050 | 38 | imported |
| 1835 | Gene.SLC3A2\|NM_001012662\|2350\|713-681 | 33 | imported |
| 1836 | Gene.SLC3A2\|NM_001012662\|2350\|681-713 | 33 | imported |
| 1837 | Gene.SLC3A2\|NM_001012662\|2350\|1034-1001 | 34 | imported |
| 1838 | Gene.SLC3A2\|NM_001012662\|2350\|1001-1034 | 34 | imported |
| 1839 | Gene.SLC3A2\|NM_001013251\|1938\|347-321 | 27 | imported |
| 1840 | Gene.SLC3A2\|NM_001013251\|1938\|321-347 | 27 | imported |
| 1841 | Gene.SLC5A8\|NM_145913\|3286\|613-589 | 25 | imported |
| 1842 | Gene.SLC5A8\|NM_145913\|3286\|589-613 | 25 | imported |
| 1843 | Gene.SLC5A8\|NM_145913\|3286\|1047-1009 | 39 | imported |
| 1844 | Gene.SLC5A8\|NM_145913\|3286\|1009-1047 | 39 | imported |
| 1845 | Gene.SLC5A8\|NM_145913\|3286\|1180-1149 | 32 | imported |
| 1846 | Gene.SLC5A8\|NM_145913\|3286\|1149-1180 | 32 | imported |
| 1847 | Gene.SLC7A11\|NM_014331\|9648\|1658-1621 | 38 | imported |
| 1848 | Gene.SLC7A11\|NM_014331\|9648\|1621-1658 | 38 | imported |
| 1849 | Gene.SLC7A11\|NM_014331\|9648\|5310-5266 | 45 | imported |
| 1850 | Gene.SLC7A11\|NM_014331\|9648\|5266-5310 | 45 | imported |
| 1851 | Gene.SLC7A11\|NM_014331\|9648\|7414-7372 | 43 | imported |
| 1852 | Gene.SLC7A11\|NM_014331\|9648\|7372-7414 | 43 | imported |
| 1853 | Gene.SMAD2\|NM_001003652\|10551\|1622-1585 | 38 | imported |
| 1854 | Gene.SMAD2\|NM_001003652\|10551\|1585-1622 | 38 | imported |
| 1855 | Gene.SMAD2\|NM_001003652\|10551\|8578-8537 | 42 | imported |
| 1856 | Gene.SMAD2\|NM_001003652\|10551\|8537-8578 | 42 | imported |
| 1857 | Gene.SMAD2\|NM_001135937\|10461\|5600-5569 | 32 | imported |
| 1858 | Gene.SMAD2\|NM_001135937\|10461\|5569-5600 | 32 | imported |
| 1859 | Gene.SMC6\|NM_001142286\|5275\|1044-1013 | 32 | imported |
| 1860 | Gene.SMC6\|NM_001142286\|5275\|1013-1044 | 32 | imported |
| 1861 | Gene.SMC6\|NM_001142286\|5275\|1753-1717 | 37 | imported |
| 1862 | Gene.SMC6\|NM_001142286\|5275\|1717-1753 | 37 | imported |
| 1863 | Gene.SMC6\|NM_001142286\|5275\|3081-3037 | 45 | imported |
| 1864 | Gene.SMC6\|NM_001142286\|5275\|3037-3081 | 45 | imported |
| 1865 | Gene.SNAI1\|NM_005985\|1722\|67-43 | 25 | imported |
| 1866 | Gene.SNAI1\|NM_005985\|1722\|43-67 | 25 | imported |
| 1867 | Gene.SNAI1\|NM_005985\|1722\|855-827 | 29 | imported |
| 1868 | Gene.SNAI1\|NM_005985\|1722\|827-855 | 29 | imported |
| 1869 | Gene.SNAI1\|NM_005985\|1722\|867-841 | 27 | imported |
| 1870 | Gene.SNAI1\|NM_005985\|1722\|841-867 | 27 | imported |
| 1871 | Gene.SNAI2\|NM_003068\|2112\|617-577 | 41 | imported |
| 1872 | Gene.SNAI2\|NM_003068\|2112\|577-617 | 41 | imported |
| 1873 | Gene.SNAI2\|NM_003068\|2112\|766-739 | 28 | imported |
| 1874 | Gene.SNAI2\|NM_003068\|2112\|739-766 | 28 | imported |
| 1875 | Gene.SNAI2\|NM_003068\|2112\|792-757 | 36 | imported |
| 1876 | Gene.SNAI2\|NM_003068\|2112\|757-792 | 36 | imported |
| 1877 | Gene.SNAI3\|NM_178310\|1713\|307-281 | 27 | imported |
| 1878 | Gene.SNAI3\|NM_178310\|1713\|281-307 | 27 | imported |
| 1879 | Gene.SNAI3\|NM_178310\|1713\|392-365 | 28 | imported |
| 1880 | Gene.SNAI3\|NM_178310\|1713\|365-392 | 28 | imported |
| 1881 | Gene.SNAI3\|NM_178310\|1713\|648-617 | 32 | imported |
| 1882 | Gene.SNAI3\|NM_178310\|1713\|617-648 | 32 | imported |
| 1883 | Gene.SNRPD3\|NM_004175\|3808\|451-417 | 35 | imported |
| 1884 | Gene.SNRPD3\|NM_004175\|3808\|417-451 | 35 | imported |
| 1885 | Gene.SNRPD3\|NM_004175\|3808\|638-609 | 30 | imported |
| 1886 | Gene.SNRPD3\|NM_004175\|3808\|609-638 | 30 | imported |
| 1887 | Gene.SNRPD3\|NM_004175\|3808\|677-641 | 37 | imported |
| 1888 | Gene.SNRPD3\|NM_004175\|3808\|641-677 | 37 | imported |
| 1889 | Gene.SOCS1\|NM_003745\|1216\|347-321 | 27 | imported |
| 1890 | Gene.SOCS1\|NM_003745\|1216\|321-347 | 27 | imported |
| 1891 | Gene.SOCS1\|NM_003745\|1216\|513-481 | 33 | imported |
| 1892 | Gene.SOCS1\|NM_003745\|1216\|481-513 | 33 | imported |
| 1893 | Gene.SOCS1\|NM_003745\|1216\|979-951 | 29 | imported |
| 1894 | Gene.SOCS1\|NM_003745\|1216\|951-979 | 29 | imported |
| 1895 | Gene.SOCS3\|NM_003955\|2737\|578-553 | 26 | imported |
| 1896 | Gene.SOCS3\|NM_003955\|2737\|553-578 | 26 | imported |
| 1897 | Gene.SOCS3\|NM_003955\|2737\|1639-1611 | 29 | imported |
| 1898 | Gene.SOCS3\|NM_003955\|2737\|1611-1639 | 29 | imported |
| 1899 | Gene.SOCS3\|NM_003955\|2737\|1915-1887 | 29 | imported |
| 1900 | Gene.SOCS3\|NM_003955\|2737\|1887-1915 | 29 | imported |
| 1901 | Gene.SOD1\|NM_000454\|981\|156-131 | 26 | imported |
| 1902 | Gene.SOD1\|NM_000454\|981\|131-156 | 26 | imported |
| 1903 | Gene.SOD1\|NM_000454\|981\|166-141 | 26 | imported |
| 1904 | Gene.SOD1\|NM_000454\|981\|141-166 | 26 | imported |
| 1905 | Gene.SOD1\|NM_000454\|981\|257-221 | 37 | imported |
| 1906 | Gene.SOD1\|NM_000454\|981\|221-257 | 37 | imported |
| 1907 | Gene.SOX10\|NM_006941\|2882\|219-193 | 27 | imported |
| 1908 | Gene.SOX10\|NM_006941\|2882\|193-219 | 27 | imported |
| 1909 | Gene.SOX10\|NM_006941\|2882\|747-721 | 27 | imported |
| 1910 | Gene.SOX10\|NM_006941\|2882\|721-747 | 27 | imported |
| 1911 | Gene.SOX10\|NM_006941\|2882\|1684-1657 | 28 | imported |
| 1912 | Gene.SOX10\|NM_006941\|2882\|1657-1684 | 28 | imported |
| 1913 | Gene.SOX2\|NM_003106\|2520\|931-904 | 28 | imported |
| 1914 | Gene.SOX2\|NM_003106\|2520\|904-931 | 28 | imported |
| 1915 | Gene.SOX2\|NM_003106\|2520\|973-946 | 28 | imported |
| 1916 | Gene.SOX2\|NM_003106\|2520\|946-973 | 28 | imported |
| 1917 | Gene.SOX2\|NM_003106\|2520\|1603-1576 | 28 | imported |
| 1918 | Gene.SOX2\|NM_003106\|2520\|1576-1603 | 28 | imported |
| 1919 | Gene.SPP1\|NM_000582\|1616\|691-659 | 33 | imported |
| 1920 | Gene.SPP1\|NM_000582\|1616\|659-691 | 33 | imported |
| 1921 | Gene.SPP1\|NM_000582\|1616\|782-757 | 26 | imported |
| 1922 | Gene.SPP1\|NM_000582\|1616\|757-782 | 26 | imported |
| 1923 | Gene.SPP1\|NM_001251829\|1511\|415-378 | 38 | imported |
| 1924 | Gene.SPP1\|NM_001251829\|1511\|378-415 | 38 | imported |
| 1925 | Gene.STAT1\|NM_007315\|4326\|1189-1153 | 37 | imported |
| 1926 | Gene.STAT1\|NM_007315\|4326\|1153-1189 | 37 | imported |
| 1927 | Gene.STAT1\|NM_007315\|4326\|2378-2341 | 38 | imported |
| 1928 | Gene.STAT1\|NM_007315\|4326\|2341-2378 | 38 | imported |
| 1929 | Gene.STAT1\|NM_007315\|4326\|2481-2449 | 33 | imported |
| 1930 | Gene.STAT1\|NM_007315\|4326\|2449-2481 | 33 | imported |
| 1931 | Gene.STAT3\|NM_003150\|4953\|2455-2420 | 36 | imported |
| 1932 | Gene.STAT3\|NM_003150\|4953\|2420-2455 | 36 | imported |
| 1933 | Gene.STAT3\|NM_139276\|4978\|1840-1807 | 34 | imported |
| 1934 | Gene.STAT3\|NM_139276\|4978\|1807-1840 | 34 | imported |
| 1935 | Gene.STAT3\|NM_213662\|4819\|156-121 | 36 | imported |
| 1936 | Gene.STAT3\|NM_213662\|4819\|121-156 | 36 | imported |
| 1937 | Gene.STAT5A\|NM_003152\|4314\|3015-2989 | 27 | imported |
| 1938 | Gene.STAT5A\|NM_003152\|4314\|2989-3015 | 27 | imported |
| 1939 | Gene.STAT5A\|NM_003152\|4314\|3126-3097 | 30 | imported |
| 1940 | Gene.STAT5A\|NM_003152\|4314\|3097-3126 | 30 | imported |
| 1941 | Gene.STAT5A\|NM_003152\|4314\|3304-3277 | 28 | imported |
| 1942 | Gene.STAT5A\|NM_003152\|4314\|3277-3304 | 28 | imported |
| 1943 | Gene.STEAP1\|NM_012449\|1330\|160-133 | 28 | imported |
| 1944 | Gene.STEAP1\|NM_012449\|1330\|133-160 | 28 | imported |
| 1945 | Gene.STEAP1\|NM_012449\|1330\|172-144 | 29 | imported |
| 1946 | Gene.STEAP1\|NM_012449\|1330\|144-172 | 29 | imported |
| 1947 | Gene.STEAP1\|NM_012449\|1330\|450-408 | 43 | imported |
| 1948 | Gene.STEAP1\|NM_012449\|1330\|408-450 | 43 | imported |
| 1949 | Gene.SWI5\|NM_001040011\|1004\|672-641 | 32 | imported |
| 1950 | Gene.SWI5\|NM_001040011\|1004\|641-672 | 32 | imported |
| 1951 | Gene.SWI5\|NM_001040011\|1004\|728-701 | 28 | imported |
| 1952 | Gene.SWI5\|NM_001040011\|1004\|701-728 | 28 | imported |
| 1953 | Gene.SWI5\|NM_001040011\|1004\|784-751 | 34 | imported |
| 1954 | Gene.SWI5\|NM_001040011\|1004\|751-784 | 34 | imported |
| 1955 | Gene.TALDO1\|NM_006755\|1319\|297-265 | 33 | imported |
| 1956 | Gene.TALDO1\|NM_006755\|1319\|265-297 | 33 | imported |
| 1957 | Gene.TALDO1\|NM_006755\|1319\|374-342 | 33 | imported |
| 1958 | Gene.TALDO1\|NM_006755\|1319\|342-374 | 33 | imported |
| 1959 | Gene.TALDO1\|NM_006755\|1319\|461-430 | 32 | imported |
| 1960 | Gene.TALDO1\|NM_006755\|1319\|430-461 | 32 | imported |
| 1961 | Gene.TDO2\|NM_005651\|1703\|438-407 | 32 | imported |
| 1962 | Gene.TDO2\|NM_005651\|1703\|407-438 | 32 | imported |
| 1963 | Gene.TDO2\|NM_005651\|1703\|1147-1107 | 41 | imported |
| 1964 | Gene.TDO2\|NM_005651\|1703\|1107-1147 | 41 | imported |
| 1965 | Gene.TDO2\|NM_005651\|1703\|1202-1163 | 40 | imported |
| 1966 | Gene.TDO2\|NM_005651\|1703\|1163-1202 | 40 | imported |
| 1967 | Gene.TDP1\|NM_001008744\|3540\|1062-1021 | 42 | imported |
| 1968 | Gene.TDP1\|NM_001008744\|3540\|1021-1062 | 42 | imported |
| 1969 | Gene.TDP1\|NM_001008744\|3540\|2795-2761 | 35 | imported |
| 1970 | Gene.TDP1\|NM_001008744\|3540\|2761-2795 | 35 | imported |
| 1971 | Gene.TDP1\|NM_018319\|3763\|56-32 | 25 | imported |
| 1972 | Gene.TDP1\|NM_018319\|3763\|32-56 | 25 | imported |
| 1973 | Gene.TDP2\|NM_016614\|1940\|710-673 | 38 | imported |
| 1974 | Gene.TDP2\|NM_016614\|1940\|673-710 | 38 | imported |
| 1975 | Gene.TDP2\|NM_016614\|1940\|852-817 | 36 | imported |
| 1976 | Gene.TDP2\|NM_016614\|1940\|817-852 | 36 | imported |
| 1977 | Gene.TDP2\|NM_016614\|1940\|1378-1345 | 34 | imported |
| 1978 | Gene.TDP2\|NM_016614\|1940\|1345-1378 | 34 | imported |
| 1979 | Gene.TEK\|NM_000459\|4787\|1873-1841 | 33 | imported |
| 1980 | Gene.TEK\|NM_000459\|4787\|1841-1873 | 33 | imported |
| 1981 | Gene.TEK\|NM_000459\|4787\|2074-2041 | 34 | imported |
| 1982 | Gene.TEK\|NM_000459\|4787\|2041-2074 | 34 | imported |
| 1983 | Gene.TEK\|NM_000459\|4787\|3832-3801 | 32 | imported |
| 1984 | Gene.TEK\|NM_000459\|4787\|3801-3832 | 32 | imported |
| 1985 | Gene.TFPI2\|NM_001271003\|2411\|112-81 | 32 | imported |
| 1986 | Gene.TFPI2\|NM_001271003\|2411\|81-112 | 32 | imported |
| 1987 | Gene.TFPI2\|NM_001271003\|2411\|2197-2161 | 37 | imported |
| 1988 | Gene.TFPI2\|NM_001271003\|2411\|2161-2197 | 37 | imported |
| 1989 | Gene.TFPI2\|NM_006528\|2444\|2362-2321 | 42 | imported |
| 1990 | Gene.TFPI2\|NM_006528\|2444\|2321-2362 | 42 | imported |
| 1991 | Gene.TGFA\|NM_001099691\|4323\|2727-2701 | 27 | imported |
| 1992 | Gene.TGFA\|NM_001099691\|4323\|2701-2727 | 27 | imported |
| 1993 | Gene.TGFA\|NM_003236\|4326\|565-541 | 25 | imported |
| 1994 | Gene.TGFA\|NM_003236\|4326\|541-565 | 25 | imported |
| 1995 | Gene.TGFA\|NM_003236\|4326\|3635-3601 | 35 | imported |
| 1996 | Gene.TGFA\|NM_003236\|4326\|3601-3635 | 35 | imported |
| 1997 | Gene.TGFB1\|NM_000660\|2741\|1391-1358 | 34 | imported |
| 1998 | Gene.TGFB1\|NM_000660\|2741\|1358-1391 | 34 | imported |
| 1999 | Gene.TGFB1\|NM_000660\|2741\|1568-1542 | 27 | imported |
| 2000 | Gene.TGFB1\|NM_000660\|2741\|1542-1568 | 27 | imported |
| 2001 | Gene.TGFB1\|NM_000660\|2741\|2284-2255 | 30 | imported |
| 2002 | Gene.TGFB1\|NM_000660\|2741\|2255-2284 | 30 | imported |
| 2003 | Gene.TGFB2\|NM_001135599\|6016\|1183-1151 | 33 | imported |
| 2004 | Gene.TGFB2\|NM_001135599\|6016\|1151-1183 | 33 | imported |
| 2005 | Gene.TGFB2\|NM_001135599\|6016\|2189-2151 | 39 | imported |
| 2006 | Gene.TGFB2\|NM_001135599\|6016\|2151-2189 | 39 | imported |
| 2007 | Gene.TGFB2\|NM_003238\|5932\|4592-4551 | 42 | imported |
| 2008 | Gene.TGFB2\|NM_003238\|5932\|4551-4592 | 42 | imported |
| 2009 | Gene.TGFB3\|NM_003239\|3510\|700-668 | 33 | imported |
| 2010 | Gene.TGFB3\|NM_003239\|3510\|668-700 | 33 | imported |
| 2011 | Gene.TGFB3\|NM_003239\|3510\|2234-2205 | 30 | imported |
| 2012 | Gene.TGFB3\|NM_003239\|3510\|2205-2234 | 30 | imported |
| 2013 | Gene.TGFB3\|NM_003239\|3510\|2260-2234 | 27 | imported |
| 2014 | Gene.TGFB3\|NM_003239\|3510\|2234-2260 | 27 | imported |
| 2015 | Gene.TGFBR1\|NM_001130916\|6285\|2687-2653 | 35 | imported |
| 2016 | Gene.TGFBR1\|NM_001130916\|6285\|2653-2687 | 35 | imported |
| 2017 | Gene.TGFBR1\|NM_001130916\|6285\|4453-4421 | 33 | imported |
| 2018 | Gene.TGFBR1\|NM_001130916\|6285\|4421-4453 | 33 | imported |
| 2019 | Gene.TGFBR1\|NM_001130916\|6285\|5713-5669 | 45 | imported |
| 2020 | Gene.TGFBR1\|NM_001130916\|6285\|5669-5713 | 45 | imported |
| 2021 | Gene.THBS2\|NM_003247\|5898\|1057-1030 | 28 | imported |
| 2022 | Gene.THBS2\|NM_003247\|5898\|1030-1057 | 28 | imported |
| 2023 | Gene.THBS2\|NM_003247\|5898\|2628-2598 | 31 | imported |
| 2024 | Gene.THBS2\|NM_003247\|5898\|2598-2628 | 31 | imported |
| 2025 | Gene.THBS2\|NM_003247\|5898\|2969-2941 | 29 | imported |
| 2026 | Gene.THBS2\|NM_003247\|5898\|2941-2969 | 29 | imported |
| 2027 | Gene.THY1\|NM_001311160\|2944\|1200-1176 | 25 | imported |
| 2028 | Gene.THY1\|NM_001311160\|2944\|1176-1200 | 25 | imported |
| 2029 | Gene.THY1\|NM_001311162\|2080\|607-579 | 29 | imported |
| 2030 | Gene.THY1\|NM_001311162\|2080\|579-607 | 29 | imported |
| 2031 | Gene.THY1\|NM_006288\|3008\|1377-1351 | 27 | imported |
| 2032 | Gene.THY1\|NM_006288\|3008\|1351-1377 | 27 | imported |
| 2033 | Gene.TIE1\|NM_001253357\|3917\|3208-3169 | 40 | imported |
| 2034 | Gene.TIE1\|NM_001253357\|3917\|3169-3208 | 40 | imported |
| 2035 | Gene.TIE1\|NM_001253357\|3917\|3466-3433 | 34 | imported |
| 2036 | Gene.TIE1\|NM_001253357\|3917\|3433-3466 | 34 | imported |
| 2037 | Gene.TIE1\|NM_005424\|4000\|2305-2278 | 28 | imported |
| 2038 | Gene.TIE1\|NM_005424\|4000\|2278-2305 | 28 | imported |
| 2039 | Gene.TIPRL\|NM_001031800\|954\|135-111 | 25 | imported |
| 2040 | Gene.TIPRL\|NM_001031800\|954\|111-135 | 25 | imported |
| 2041 | Gene.TIPRL\|NM_001031800\|954\|521-491 | 31 | imported |
| 2042 | Gene.TIPRL\|NM_001031800\|954\|491-521 | 31 | imported |
| 2043 | Gene.TIPRL\|NM_152902\|3120\|1318-1275 | 44 | imported |
| 2044 | Gene.TIPRL\|NM_152902\|3120\|1275-1318 | 44 | imported |
| 2045 | Gene.TK1\|NM_003258\|1616\|336-309 | 28 | imported |
| 2046 | Gene.TK1\|NM_003258\|1616\|309-336 | 28 | imported |
| 2047 | Gene.TK1\|NM_003258\|1616\|349-323 | 27 | imported |
| 2048 | Gene.TK1\|NM_003258\|1616\|323-349 | 27 | imported |
| 2049 | Gene.TK1\|NM_003258\|1616\|502-477 | 26 | imported |
| 2050 | Gene.TK1\|NM_003258\|1616\|477-502 | 26 | imported |
| 2051 | Gene.TKTL1\|NM_001145933\|2634\|1007-969 | 39 | imported |
| 2052 | Gene.TKTL1\|NM_001145933\|2634\|969-1007 | 39 | imported |
| 2053 | Gene.TKTL1\|NM_001145934\|2579\|424-397 | 28 | imported |
| 2054 | Gene.TKTL1\|NM_001145934\|2579\|397-424 | 28 | imported |
| 2055 | Gene.TKTL1\|NM_001145934\|2579\|1679-1651 | 29 | imported |
| 2056 | Gene.TKTL1\|NM_001145934\|2579\|1651-1679 | 29 | imported |
| 2057 | Gene.TNC\|NM_002160\|8605\|102-73 | 30 | imported |
| 2058 | Gene.TNC\|NM_002160\|8605\|73-102 | 30 | imported |
| 2059 | Gene.TNC\|NM_002160\|8605\|2626-2593 | 34 | imported |
| 2060 | Gene.TNC\|NM_002160\|8605\|2593-2626 | 34 | imported |
| 2061 | Gene.TNC\|NM_002160\|8605\|4495-4465 | 31 | imported |
| 2062 | Gene.TNC\|NM_002160\|8605\|4465-4495 | 31 | imported |
| 2063 | Gene.TNF\|NM_000594\|1686\|478-449 | 30 | imported |
| 2064 | Gene.TNF\|NM_000594\|1686\|449-478 | 30 | imported |
| 2065 | Gene.TNF\|NM_000594\|1686\|573-547 | 27 | imported |
| 2066 | Gene.TNF\|NM_000594\|1686\|547-573 | 27 | imported |
| 2067 | Gene.TNF\|NM_000594\|1686\|815-785 | 31 | imported |
| 2068 | Gene.TNF\|NM_000594\|1686\|785-815 | 31 | imported |
| 2069 | Gene.TNFRSF10A\|NM_003844\|1764\|405-376 | 30 | imported |
| 2070 | Gene.TNFRSF10A\|NM_003844\|1764\|376-405 | 30 | imported |
| 2071 | Gene.TNFRSF10A\|NM_003844\|1764\|497-466 | 32 | imported |
| 2072 | Gene.TNFRSF10A\|NM_003844\|1764\|466-497 | 32 | imported |
| 2073 | Gene.TNFRSF10A\|NM_003844\|1764\|1008-976 | 33 | imported |
| 2074 | Gene.TNFRSF10A\|NM_003844\|1764\|976-1008 | 33 | imported |
| 2075 | Gene.TNFRSF10B\|NM_003842\|4154\|517-491 | 27 | imported |
| 2076 | Gene.TNFRSF10B\|NM_003842\|4154\|491-517 | 27 | imported |
| 2077 | Gene.TNFRSF10B\|NM_003842\|4154\|1401-1366 | 36 | imported |
| 2078 | Gene.TNFRSF10B\|NM_003842\|4154\|1366-1401 | 36 | imported |
| 2079 | Gene.TNFRSF10B\|NM_147187\|4067\|680-647 | 34 | imported |
| 2080 | Gene.TNFRSF10B\|NM_147187\|4067\|647-680 | 34 | imported |
| 2081 | Gene.TNFRSF1A\|NM_001065\|2258\|165-134 | 32 | imported |
| 2082 | Gene.TNFRSF1A\|NM_001065\|2258\|134-165 | 32 | imported |
| 2083 | Gene.TNFRSF1A\|NM_001065\|2258\|311-286 | 26 | imported |
| 2084 | Gene.TNFRSF1A\|NM_001065\|2258\|286-311 | 26 | imported |
| 2085 | Gene.TNFRSF1A\|NM_001065\|2258\|1263-1236 | 28 | imported |
| 2086 | Gene.TNFRSF1A\|NM_001065\|2258\|1236-1263 | 28 | imported |
| 2087 | Gene.TOPBP1\|NM_007027\|5378\|1483-1441 | 43 | imported |
| 2088 | Gene.TOPBP1\|NM_007027\|5378\|1441-1483 | 43 | imported |
| 2089 | Gene.TOPBP1\|NM_007027\|5378\|2684-2656 | 29 | imported |
| 2090 | Gene.TOPBP1\|NM_007027\|5378\|2656-2684 | 29 | imported |
| 2091 | Gene.TOPBP1\|NM_007027\|5378\|3144-3106 | 39 | imported |
| 2092 | Gene.TOPBP1\|NM_007027\|5378\|3106-3144 | 39 | imported |
| 2093 | Gene.TP53\|NM_000546\|2591\|1126-1101 | 26 | imported |
| 2094 | Gene.TP53\|NM_000546\|2591\|1101-1126 | 26 | imported |
| 2095 | Gene.TP53\|NM_000546\|2591\|1920-1893 | 28 | imported |
| 2096 | Gene.TP53\|NM_000546\|2591\|1893-1920 | 28 | imported |
| 2097 | Gene.TP53\|NM_000546\|2591\|2126-2091 | 36 | imported |
| 2098 | Gene.TP53\|NM_000546\|2591\|2091-2126 | 36 | imported |
| 2099 | Gene.TP63\|NM_001114979\|2870\|841-817 | 25 | imported |
| 2100 | Gene.TP63\|NM_001114979\|2870\|817-841 | 25 | imported |
| 2101 | Gene.TP63\|NM_001114979\|2870\|968-937 | 32 | imported |
| 2102 | Gene.TP63\|NM_001114979\|2870\|937-968 | 32 | imported |
| 2103 | Gene.TP63\|NM_001114979\|2870\|1131-1105 | 27 | imported |
| 2104 | Gene.TP63\|NM_001114979\|2870\|1105-1131 | 27 | imported |
| 2105 | Gene.TP73\|NM_001126242\|4982\|3725-3697 | 29 | imported |
| 2106 | Gene.TP73\|NM_001126242\|4982\|3697-3725 | 29 | imported |
| 2107 | Gene.TP73\|NM_001126242\|4982\|3931-3907 | 25 | imported |
| 2108 | Gene.TP73\|NM_001126242\|4982\|3907-3931 | 25 | imported |
| 2109 | Gene.TP73\|NM_001204184\|5060\|1159-1135 | 25 | imported |
| 2110 | Gene.TP73\|NM_001204184\|5060\|1135-1159 | 25 | imported |
| 2111 | Gene.TWIST1\|NM_000474\|1669\|42-15 | 28 | imported |
| 2112 | Gene.TWIST1\|NM_000474\|1669\|15-42 | 28 | imported |
| 2113 | Gene.TWIST1\|NM_000474\|1669\|1166-1135 | 32 | imported |
| 2114 | Gene.TWIST1\|NM_000474\|1669\|1135-1166 | 32 | imported |
| 2115 | Gene.TWIST1\|NM_000474\|1669\|1190-1163 | 28 | imported |
| 2116 | Gene.TWIST1\|NM_000474\|1669\|1163-1190 | 28 | imported |
| 2117 | Gene.UIMC1\|NM_001199297\|2840\|2194-2161 | 34 | imported |
| 2118 | Gene.UIMC1\|NM_001199297\|2840\|2161-2194 | 34 | imported |
| 2119 | Gene.UIMC1\|NM_001199297\|2840\|2336-2305 | 32 | imported |
| 2120 | Gene.UIMC1\|NM_001199297\|2840\|2305-2336 | 32 | imported |
| 2121 | Gene.UIMC1\|NM_001199298\|2615\|2194-2157 | 38 | imported |
| 2122 | Gene.UIMC1\|NM_001199298\|2615\|2157-2194 | 38 | imported |
| 2123 | Gene.ULK1\|NM_003565\|5239\|597-573 | 25 | imported |
| 2124 | Gene.ULK1\|NM_003565\|5239\|573-597 | 25 | imported |
| 2125 | Gene.ULK1\|NM_003565\|5239\|3062-3037 | 26 | imported |
| 2126 | Gene.ULK1\|NM_003565\|5239\|3037-3062 | 26 | imported |
| 2127 | Gene.ULK1\|NM_003565\|5239\|3767-3741 | 27 | imported |
| 2128 | Gene.ULK1\|NM_003565\|5239\|3741-3767 | 27 | imported |
| 2129 | Gene.ULK2\|NM_001142610\|3924\|159-133 | 27 | imported |
| 2130 | Gene.ULK2\|NM_001142610\|3924\|133-159 | 27 | imported |
| 2131 | Gene.ULK2\|NM_001142610\|3924\|1643-1618 | 26 | imported |
| 2132 | Gene.ULK2\|NM_001142610\|3924\|1618-1643 | 26 | imported |
| 2133 | Gene.ULK2\|NM_001142610\|3924\|1783-1750 | 34 | imported |
| 2134 | Gene.ULK2\|NM_001142610\|3924\|1750-1783 | 34 | imported |
| 2135 | Gene.UVRAG\|NM_003369\|5166\|550-517 | 34 | imported |
| 2136 | Gene.UVRAG\|NM_003369\|5166\|517-550 | 34 | imported |
| 2137 | Gene.UVRAG\|NM_003369\|5166\|776-732 | 45 | imported |
| 2138 | Gene.UVRAG\|NM_003369\|5166\|732-776 | 45 | imported |
| 2139 | Gene.UVRAG\|NM_003369\|5166\|2829-2796 | 34 | imported |
| 2140 | Gene.UVRAG\|NM_003369\|5166\|2796-2829 | 34 | imported |
| 2141 | Gene.VCP\|NM_007126\|3859\|1283-1249 | 35 | imported |
| 2142 | Gene.VCP\|NM_007126\|3859\|1249-1283 | 35 | imported |
| 2143 | Gene.VCP\|NM_007126\|3859\|1510-1473 | 38 | imported |
| 2144 | Gene.VCP\|NM_007126\|3859\|1473-1510 | 38 | imported |
| 2145 | Gene.VCP\|NM_007126\|3859\|1925-1889 | 37 | imported |
| 2146 | Gene.VCP\|NM_007126\|3859\|1889-1925 | 37 | imported |
| 2147 | Gene.VEGFA\|NM_001025366\|3677\|430-404 | 27 | imported |
| 2148 | Gene.VEGFA\|NM_001025366\|3677\|404-430 | 27 | imported |
| 2149 | Gene.VEGFA\|NM_001025368\|3554\|3101-3061 | 41 | imported |
| 2150 | Gene.VEGFA\|NM_001025368\|3554\|3061-3101 | 41 | imported |
| 2151 | Gene.VEGFA\|NM_001025369\|3519\|1163-1132 | 32 | imported |
| 2152 | Gene.VEGFA\|NM_001025369\|3519\|1132-1163 | 32 | imported |
| 2153 | Gene.VEGFC\|NM_005429\|2103\|783-739 | 45 | imported |
| 2154 | Gene.VEGFC\|NM_005429\|2103\|739-783 | 45 | imported |
| 2155 | Gene.VEGFC\|NM_005429\|2103\|1106-1063 | 44 | imported |
| 2156 | Gene.VEGFC\|NM_005429\|2103\|1063-1106 | 44 | imported |
| 2157 | Gene.VEGFC\|NM_005429\|2103\|1258-1225 | 34 | imported |
| 2158 | Gene.VEGFC\|NM_005429\|2103\|1225-1258 | 34 | imported |
| 2159 | Gene.VIM\|NM_003380\|2151\|1149-1117 | 33 | imported |
| 2160 | Gene.VIM\|NM_003380\|2151\|1117-1149 | 33 | imported |
| 2161 | Gene.VIM\|NM_003380\|2151\|1256-1225 | 32 | imported |
| 2162 | Gene.VIM\|NM_003380\|2151\|1225-1256 | 32 | imported |
| 2163 | Gene.VIM\|NM_003380\|2151\|1419-1387 | 33 | imported |
| 2164 | Gene.VIM\|NM_003380\|2151\|1387-1419 | 33 | imported |
| 2165 | Gene.WNT11\|NM_004626\|1927\|994-961 | 34 | imported |
| 2166 | Gene.WNT11\|NM_004626\|1927\|961-994 | 34 | imported |
| 2167 | Gene.WNT11\|NM_004626\|1927\|1298-1265 | 34 | imported |
| 2168 | Gene.WNT11\|NM_004626\|1927\|1265-1298 | 34 | imported |
| 2169 | Gene.WNT11\|NM_004626\|1927\|1519-1489 | 31 | imported |
| 2170 | Gene.WNT11\|NM_004626\|1927\|1489-1519 | 31 | imported |
| 2171 | Gene.WNT16B\|NM_016087\|2894\|680-649 | 32 | imported |
| 2172 | Gene.WNT16B\|NM_016087\|2894\|649-680 | 32 | imported |
| 2173 | Gene.WNT16B\|NM_016087\|2894\|1162-1129 | 34 | imported |
| 2174 | Gene.WNT16B\|NM_016087\|2894\|1129-1162 | 34 | imported |
| 2175 | Gene.WNT16B\|NM_057168\|3132\|522-495 | 28 | imported |
| 2176 | Gene.WNT16B\|NM_057168\|3132\|495-522 | 28 | imported |
| 2177 | Gene.WNT3A\|NM_033131\|2988\|176-151 | 26 | imported |
| 2178 | Gene.WNT3A\|NM_033131\|2988\|151-176 | 26 | imported |
| 2179 | Gene.WNT3A\|NM_033131\|2988\|1150-1126 | 25 | imported |
| 2180 | Gene.WNT3A\|NM_033131\|2988\|1126-1150 | 25 | imported |
| 2181 | Gene.WNT3A\|NM_033131\|2988\|1403-1376 | 28 | imported |
| 2182 | Gene.WNT3A\|NM_033131\|2988\|1376-1403 | 28 | imported |
| 2183 | Gene.WNT5A\|NM_001256105\|5599\|1012-988 | 25 | imported |
| 2184 | Gene.WNT5A\|NM_001256105\|5599\|988-1012 | 25 | imported |
| 2185 | Gene.WNT5A\|NM_001256105\|5599\|3095-3056 | 40 | imported |
| 2186 | Gene.WNT5A\|NM_001256105\|5599\|3056-3095 | 40 | imported |
| 2187 | Gene.WNT5A\|NM_003392\|6194\|5655-5617 | 39 | imported |
| 2188 | Gene.WNT5A\|NM_003392\|6194\|5617-5655 | 39 | imported |
| 2189 | Gene.WNT5B\|NM_030775\|2184\|1671-1639 | 33 | imported |
| 2190 | Gene.WNT5B\|NM_030775\|2184\|1639-1671 | 33 | imported |
| 2191 | Gene.WNT5B\|NM_030775\|2184\|1906-1873 | 34 | imported |
| 2192 | Gene.WNT5B\|NM_030775\|2184\|1873-1906 | 34 | imported |
| 2193 | Gene.WNT5B\|NM_030775\|2184\|1938-1909 | 30 | imported |
| 2194 | Gene.WNT5B\|NM_030775\|2184\|1909-1938 | 30 | imported |
| 2195 | Gene.WRN\|NM_000553\|5765\|1097-1057 | 41 | imported |
| 2196 | Gene.WRN\|NM_000553\|5765\|1057-1097 | 41 | imported |
| 2197 | Gene.WRN\|NM_000553\|5765\|1429-1393 | 37 | imported |
| 2198 | Gene.WRN\|NM_000553\|5765\|1393-1429 | 37 | imported |
| 2199 | Gene.WRN\|NM_000553\|5765\|4074-4033 | 42 | imported |
| 2200 | Gene.WRN\|NM_000553\|5765\|4033-4074 | 42 | imported |
| 2201 | Gene.XIAP\|NM_001167\|8460\|1095-1066 | 30 | imported |
| 2202 | Gene.XIAP\|NM_001167\|8460\|1066-1095 | 30 | imported |
| 2203 | Gene.XIAP\|NM_001167\|8460\|7627-7598 | 30 | imported |
| 2204 | Gene.XIAP\|NM_001167\|8460\|7598-7627 | 30 | imported |
| 2205 | Gene.XIAP\|NM_001204401\|8427\|4452-4411 | 42 | imported |
| 2206 | Gene.XIAP\|NM_001204401\|8427\|4411-4452 | 42 | imported |
| 2207 | Gene.XRCC1\|NM_006297\|2102\|267-235 | 33 | imported |
| 2208 | Gene.XRCC1\|NM_006297\|2102\|235-267 | 33 | imported |
| 2209 | Gene.XRCC1\|NM_006297\|2102\|334-307 | 28 | imported |
| 2210 | Gene.XRCC1\|NM_006297\|2102\|307-334 | 28 | imported |
| 2211 | Gene.XRCC1\|NM_006297\|2102\|1885-1855 | 31 | imported |
| 2212 | Gene.XRCC1\|NM_006297\|2102\|1855-1885 | 31 | imported |
| 2213 | Gene.XRCC2\|NM_005431\|3094\|302-261 | 42 | imported |
| 2214 | Gene.XRCC2\|NM_005431\|3094\|261-302 | 42 | imported |
| 2215 | Gene.XRCC2\|NM_005431\|3094\|643-599 | 45 | imported |
| 2216 | Gene.XRCC2\|NM_005431\|3094\|599-643 | 45 | imported |
| 2217 | Gene.XRCC2\|NM_005431\|3094\|816-781 | 36 | imported |
| 2218 | Gene.XRCC2\|NM_005431\|3094\|781-816 | 36 | imported |
| 2219 | Gene.XRCC3\|NM_001100118\|2563\|268-232 | 37 | imported |
| 2220 | Gene.XRCC3\|NM_001100118\|2563\|232-268 | 37 | imported |
| 2221 | Gene.XRCC3\|NM_001100118\|2563\|491-463 | 29 | imported |
| 2222 | Gene.XRCC3\|NM_001100118\|2563\|463-491 | 29 | imported |
| 2223 | Gene.XRCC3\|NM_001100118\|2563\|1687-1660 | 28 | imported |
| 2224 | Gene.XRCC3\|NM_001100118\|2563\|1660-1687 | 28 | imported |
| 2225 | Gene.XRCC4\|NM_003401\|1777\|981-946 | 36 | imported |
| 2226 | Gene.XRCC4\|NM_003401\|1777\|946-981 | 36 | imported |
| 2227 | Gene.XRCC4\|NM_003401\|1777\|1148-1111 | 38 | imported |
| 2228 | Gene.XRCC4\|NM_003401\|1777\|1111-1148 | 38 | imported |
| 2229 | Gene.XRCC4\|NM_022550\|1824\|682-646 | 37 | imported |
| 2230 | Gene.XRCC4\|NM_022550\|1824\|646-682 | 37 | imported |
| 2231 | Gene.XRCC5\|NM_021141\|3448\|2212-2176 | 37 | imported |
| 2232 | Gene.XRCC5\|NM_021141\|3448\|2176-2212 | 37 | imported |
| 2233 | Gene.XRCC5\|NM_021141\|3448\|2237-2205 | 33 | imported |
| 2234 | Gene.XRCC5\|NM_021141\|3448\|2205-2237 | 33 | imported |
| 2235 | Gene.XRCC5\|NM_021141\|3448\|1884-1857 | 28 | imported |
| 2236 | Gene.XRCC5\|NM_021141\|3448\|1857-1884 | 28 | imported |
| 2237 | Gene.XRCC6\|NM_001469\|2284\|790-761 | 30 | imported |
| 2238 | Gene.XRCC6\|NM_001469\|2284\|761-790 | 30 | imported |
| 2239 | Gene.XRCC6\|NM_001469\|2284\|856-818 | 39 | imported |
| 2240 | Gene.XRCC6\|NM_001469\|2284\|818-856 | 39 | imported |
| 2241 | Gene.XRCC6\|NM_001469\|2284\|908-875 | 34 | imported |
| 2242 | Gene.XRCC6\|NM_001469\|2284\|875-908 | 34 | imported |
| 2243 | Gene.ZEB1\|NM_001128128\|6278\|1285-1249 | 37 | imported |
| 2244 | Gene.ZEB1\|NM_001128128\|6278\|1249-1285 | 37 | imported |
| 2245 | Gene.ZEB1\|NM_001128128\|6278\|1538-1509 | 30 | imported |
| 2246 | Gene.ZEB1\|NM_001128128\|6278\|1509-1538 | 30 | imported |
| 2247 | Gene.ZEB1\|NM_001174096\|6001\|983-951 | 33 | imported |
| 2248 | Gene.ZEB1\|NM_001174096\|6001\|951-983 | 33 | imported |
| 2249 | Gene.ZEB2\|NM_001171653\|9171\|1803-1772 | 32 | imported |
| 2250 | Gene.ZEB2\|NM_001171653\|9171\|1772-1803 | 32 | imported |
| 2251 | Gene.ZEB2\|NM_001171653\|9171\|8589-8548 | 42 | imported |
| 2252 | Gene.ZEB2\|NM_001171653\|9171\|8548-8589 | 42 | imported |
| 2253 | Gene.ZEB2\|NM_014795\|9243\|6821-6777 | 45 | imported |
| 2254 | Gene.ZEB2\|NM_014795\|9243\|6777-6821 | 45 | imported |
