# Supplementary material for: Transcriptomic Landscape of Cisplatin-Resistant Neuroblastoma Cells
Source: Cells. 2019 Mar 12;8(3):235. doi: 10.3390/cells8030235 (PMC6469049; doi:10.3390/cells8030235)
Supplement: Supplementary file 1 [file cells-08-00235-s001.zip › Supplementary Table 2_R1.docx]

**Supplementary Table 2.** List of primers employed for validation of selected microarray results using q-PCR.

| **Gene** | **Symbol** | **Primer pair**  **(5´-3´)*** | **T*_m_*** |
| --- | --- | --- | --- |
| Metallothionein 3 | *MT3* | ATGGACCCTGAGACCTGCCC  TCACTGGCAGCAGCTGCACT | 60.0 |
| ZnT-Like Transporter 1 | *SLC30A5* | CTATTACCAAACACCAGTGG  TTGTCTTTGCTGGTCCTCCT | 60.0 |
| ZnT-Like Transporter 2 | *SLC30A7* | CACTTTCATTCTCATGATGGCCC  GCTATAAGAATTGAACAGATAGGA | 59.9 |
| ATP Binding Cassette Subfamily B Member 5 | *ABCB5* | AGTCTGGAGAGACAGTCGCCT  TACTGATGGTGGTCCCGAAC | 60.0 |
| Tumor Protein P53 | *TP53* | TAACAGTTCCTGCATGGGCGGC  AGGACAGGCACAAACACGCACC | 60.1 |
| Bcl-2-like protein 1 | *BCL2L1* | CTCTAACCATGGTGGGCACT  TGTCTGGTCACTTCCGACTG | 59.9 |
| Caspase 8 | *CASP8* | ACAAGACCCACGTGGAGAAG  ACTTCCTCTAGGTGGCAGCA | 60.0 |
| 18S ribosomal RNA (control) | *18S* | CGGCTACCACATCCAAGGAA  GCTGGAATTACCGCGGCTGC | 60.0 |

*Upper and lower sequences represent forward and reverse primers, respectively; T*_m_* is melting temperature of a specific product.
